# Screening of differentially expressed microRNAs and target genes in two potato varieties under nitrogen stress

**DOI:** 10.1186/s12870-022-03866-5

**Published:** 2022-10-08

**Authors:** Yue Lu, Jingying Zhang, Zhijun Han, Zhongcai Han, Shuang Li, Jiayue Zhang, Haoran Ma, Yuzhu Han

**Affiliations:** 1 College of Horticulture Research, Jilin Agricultural University, Changchun City, 130118 People’s Republic of China; 2 College of Resources and Environment, Jilin Agricultural University, Changchun City, 130118 P.R. China; 3 Jilin Provincial Research Institute of Vegetables and Flowers, Changchun City, 130052 People’s Republic of China; 4 Teaching and Research Base Management Office, Jilin Agricultural University, Changchun City, 130118 People’s Republic of China

**Keywords:** Potato, N stress, miRNAs, Target genes, Luciferase activity measurement

## Abstract

**Background:**

A reasonable supply of nitrogen (N) fertilizer is essential for obtaining high-quality, high-level, and stable potato yields, and an improvement in the N utilization efficiency can effectively reduce N fertilizer use. It is important to use accurate, straightforward, and efficient transgenic breeding techniques for the identification of genes that can improve nitrogen use efficiency, thus enabling us to achieve the ultimate goal of breeding N-efficient potato varieties. In recent years, some of the mechanisms of miRNAs have been elucidated via the analysis of the correlation between the expression levels of potato miRNA target genes and regulated genes under conditions of stress, but the role of miRNAs in the inhibition/expression of key genes regulating N metabolism under N stress is still unclear. Our study aimed to identify the role played by specific enzymes and miRNAs in the responses of plants to N stress.

**Results:**

The roots and leaves of the N-efficient potato variety, Yanshu4 ("Y"), and N-inefficient potato variety, Atlantic ("D"), were collected at the seedling and budding stages after they were exposed to different N fertilizer treatments. The miRNAs expressed differentially under the two types of N stress and their corresponding target genes were first predicted using miRNA and degradome analysis. Then, quantitative polymerase chain reaction (qRT-PCR) was performed to verify the expression of differential miRNAs that were closely related to N metabolism. Finally, the shearing relationship between *stu-miR396-5p* and its target gene *StNiR* was determined by analyzing luciferase activity levels. The results showed that NiR activity increased significantly with an increase in the applied N levels from the seedling stage to the budding stage, and *NiR* responded significantly to different N treatments. miRNA sequencing enabled us to predict 48 families with conserved miRNAs that were mainly involved in N metabolism, carbon metabolism, and amino acid biosynthesis. The differences in the expression of the following miRNAs were identified via screening (high expression levels and *P* < 0.05): *stu-miR396-5p*, *stu-miR408b-3p_R-1*, *stu-miR3627-3p*, *stu-miR482a-3p*, *stu-miR8036-3p*, *stu-miR482a-5p*, *stu-miR827-5p*, *stu-miR156a_L-1*, *stu-miR827-3p*, *stu-miR172b-5p*, *stu-miR6022-p3_7*, *stu-miR398a-5p*, and *stu-miR166c-5p_L-3*. Degradome analysis showed that most miRNAs had many-to-many relationships with target genes. The main target genes involved in N metabolism were *NiR*, *NiR1*, *NRT2.5*, and *NRT2.7*. qRT-PCR analysis showed that there were significant differences in the expression levels of *stu-miR396-5p*, *stu-miR8036-3p*, and *stu-miR482a-3p* in the leaves and roots of the Yanshu4 and Atlantic varieties at the seedling and budding stages under conditions that involved no N and excessive N application; the expression of these miRNAs was induced in response to N stress. The correlation between the differential expression of *stu-miR396-5p* and its corresponding target gene *NiR* was further verified by determining the luciferase activity level and was found to be strongly negative.

**Conclusion:**

The activity of NiR was significantly positively correlated with N application from the seedling to the budding stage. Differential miRNAs and target genes showed a many-to-many relationship with each other. The expression of *stu-miR396-5p*, *stu-miR482a-3p*, and *stu-miR8036-3p* in the roots and leaves of the Yanshu4 and Atlantic varieties at the seedling and budding stages was notably different under two types of N stress. Under two types of N stress, *stu-miR396-5p* was down-regulated in Yanshu4 in the seedling-stage and shoot-stage roots, and up-regulated in seedling-stage roots and shoot-stage leaves; *stu-miR482a-3p* was up-regulated in the seedling and shoot stages. The expression of *stu-miR8036-3p* was up-regulated in the leaves and roots at the seedling and budding stages, and down-regulated in roots under both types of N stress. The gene expressing the key enzyme involved in N metabolism, *StNiR*, and the *stu-miR396-5p* luciferase assay reporter gene had a strong regulatory relationship with each other. This study provides candidate miRNAs related to nitrogen metabolism and highlights that differential miRNAs play a key role in nitrogen stress in potato, providing insights for future research on miRNAs and their target genes in nitrogen metabolic pathways and breeding nitrogen-efficient potatoes.

**Supplementary Information:**

The online version contains supplementary material available at 10.1186/s12870-022-03866-5.

## Introduction

Potato (*Solanum tuberosum* L.) is the fourth most commonly cultivated food crop worldwide [1]. Nitrogen (N) fertilizer application is closely related to potato quality and yield. In order to ensure high and stable yields, China's fertilizer input has accounted for 31% of the world's total in recent years, and four times the world’s average [2]. Excessive N application would not only lead to the phenomenon of "high fertilizer inefficiency", but also result in a negative impact on the environment and economy [3]. For example, excessive N application in maize is found to lead to water deficit, which in turn causes several problems, such as reduced fertilizer utilization and photosynthetic rate [4]. The improvement of the N use efficiency (NUE) of crops to obtain an equal or higher yield with a lower level of N is considered an effective method for solving this problem.

Studies have shown that the NUE of potato plants could be improved only to a limited extent via the selection of high-yielding varieties or soil management, and that it was difficult to make further improvements [5]. Therefore, by regulating the N stress in potato plants, the roles of differential microRNAs (miRNAs) and corresponding target genes involved in N metabolism pathways were analyzed, and the NUE of potato plants was improved, to achieve high and stable potato yields. According to previous studies, under N stress, nitrate transporter (NRT) protein, nitrate reductase (NR), glutamine synthase (GS), glutamate dehydrogenase (GDH), and nitrite reductase (NiR) respond to different N treatments in two potato varieties [6]. NiR is the second enzyme involved in the nitrate reduction process, which directly reflects the nutritional status and N assimilation level of plants to a certain extent. The expression of *StNiR*s was found to be significantly different under different types of N treatment in potato plants, and the activity and expression of *StNiR* increased significantly with an increase in the N supply. This confirmed that *StNiR*s could be used to regulate N metabolism in potato plants during N uptake and assimilation. [7].

miRNAs are endogenous non-coding RNAs with regulatory functions that are approximately 18–25 nucleotides (nt) in length. The target gene is recognized by complementary base pairing, and the silencing complex degrades the target gene or inhibits its translation based on the degree of complementarity [8], Thus, miRNAs participate in many growth and development processes such as cell signal transduction and development, and generation of responses to biotic and abiotic stresses [9]. In barley and gramineous crops such as wheat and corn [10], miR396 is considered to be a metabolic sensor that regulates plant N balance [11]. miR169 and the target gene *NFYA* were reported to be significantly negatively regulated under N stress in maize [12]. miR174 and miR167 are involved in N signaling during root development [13], and miR156, miR166, and miR169 are up-regulated during plant growth under conditions of N deficiency [14]. The regulatory pattern of miR159-regulated target gene *MYB* and miR169-regulated target gene *NFYA* in potato drought stress has been confirmed [15]. Therefore, we speculate that the screened differential miRNAs and their target genes have regulatory relationships in N metabolism pathways, and that they work together to regulate N metabolism. In conventional crop production, 'YanShu4' is a high-absorption and high-utilization potato variety, while 'Atlantic' is a low-absorption and low-utilization potato variety [1]. Therefore, the two types of potatoes were not treated with N and were treated with excessive levels of N. The roots and leaves of the two types of potatoes at the seedling and budding stages were subjected to miRNA sequencing and degradome sequencing. In this study, a total of 1439 miRNAs were predicted; of these, 798 miRNAs had been reported previously, 349 miRNAs were novel, and 13 miRNAs that were closely related to N metabolism were mainly screened for conducting an in-depth study. The sequencing and quantitative polymerase chain reaction (qRT-PCR) results were verified, and the differences in miRNA expression levels under N stress were identified via screening. The responsiveness of the differential miRNAs under N stress as well as the differential expression in the two types of potato plants different periods and sites were clarified, in order to provide a theoretical basis for the further examination of N metabolism-related differential miRNAs and identification of their target genes. In this study, 13 candidate miRNAs related to nitrogen metabolism were screened by microRNA sequencing, and the binding site prediction and luciferase validation of NiR, a key gene in nitrogen metabolism pathway, and its corresponding miR396-5p confirmed the existence of a shearing relationship. The expression analysis revealed that the two genes are negatively regulated, which can be used as a reference for the subsequent studies.

## Results

### miRNA sequencing and analysis

The RNA sequences extracted from potato leaves and roots were sequenced, and the output raw sequencing data (Additional file 1, Table 1) were counted to obtain the unique sequence data and the copy number corresponding to each unique sequence. First, the 3' adapter sequence was excluded from the original sequencing data. Simultaneously, the sequence and target genes were compared to the Rfam (including rRNA, tRNA, snRNA, snoRNA, etc.) and Repbase databases and then filtered. The filtered data were called valid data, and they were processed further to facilitate miRNA alignment, identification, prediction, and analysis. Clear adapter trimming, quality trimming, read label unification, and mapping of data to Rfam were performed. Finally, clean reads were obtained after duplicate removing and read length filtering (size: 18–25 nt).

**Fig. 1:**
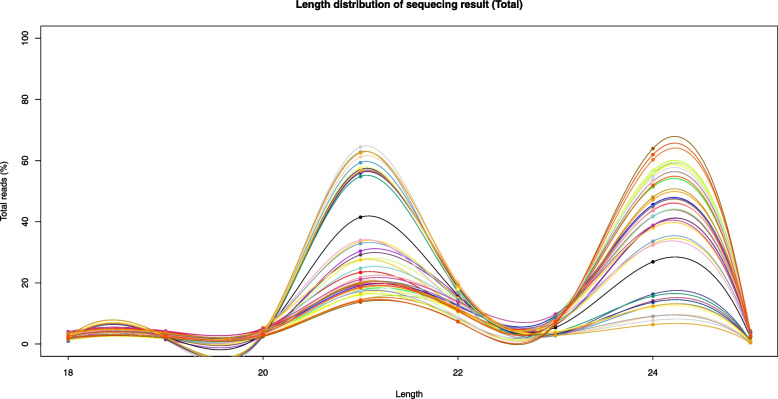
Length distribution of miRNAs

Based on the analysis and statistics of the original sequencing data, the statistical analysis of the length distribution of the clean data was performed. According to the typical characteristics of Dicer digestion, most of the data were distributed within 20–24 nt. Figure 1 shows the length distribution of miRNA clean reads obtained by miRNA sequencing under N stress; the results showed that the largest miRNAs were 25 nt in length, followed by miRNAs with a length of 24 nt.

### Differential miRNA expression analysis

T-test was used to screen genes with significant differences and differentially expressed genes, with *P* <  = 0.05 as the threshold (see Additional file 1, Table 2 for details). miRNA sequencing enabled us to predict that there were 48 conserved miRNAs in a family. Based on the results of miRNA sequencing and degradome analysis, it was found that the differentially expressed miRNAs were involved in a large number of metabolic pathways and biological processes, and many of these miRNAs had a one-to-many relationship with target genes.

The overall distribution of differentially expressed miRNAs can be visualized by constructing volcano plots. It can be seen from Fig. 1 (in Additional file 2) that there were notable differences in the expression patterns in the leaves of the two types of potatoes without treatment with N at the germination stage. We performed a comparison of seedlings and budding stage leaves under excessive N application and no N application in two kinds of potato plants. Under conditions of excessive N application, the miRNA expression levels in leaves of the two kinds of potato plants were significantly up-regulated, and when N was not applied, the miRNA expression levels in leaves of the two kinds of potatoes were significantly down-regulated.

### Statistical analysis of differentially up- and down-regulated miRNAs

**Fig. 2:**
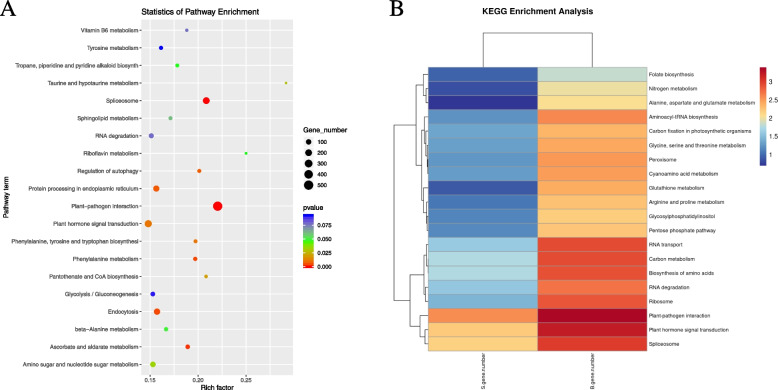
Differences miRNAs corresponding target gene KEGG enrichment analysis number is the number of genes matched to a single KEGG with significant differences, B gene number is the number of genes matched to a single KEGG

Statistics of up- and down-regulated genes can determine the number of differentially expressed miRNAs under different experimental conditions. A comparison of miRNAs in different groups showed that the numbers of up- and down-regulated miRNAs in N-fertilized leaves were 151 and 181, respectively. The numbers of up- and down-regulated miRNAs in the corresponding non-N-treated leaves of the two potato plants were 146 and 180, respectively. No miRNAs were up-regulated in the roots at the seedling and budding stages in the Yanshu4 variety under excessive N application treatment, at the seedling and budding stages of the Atlantic variety under no N treatment, and at the budding stage of the Yanshu4 variety under both N treatments. Simultaneously, there were no up-regulated miRNAs in the leaves at the budding stage in the non-N treated Atlantic variety and in the roots of two kinds of non-N treated potato plants at the budding stage, as shown in Fig. 2 in Additional file 2.

### Cluster analysis of differential miRNAs

Cluster expression models resulted in better and more intuitive cluster graphs. KEGG enrichment analysis of the target genes corresponding to differential miRNAs showed that *stu-miR396-5p*, *stu-miR482a-3p*, and *stu-miR8036-3p* were expressed in response to two types of N stress, in two potatoes, at two periods, and in two tissues. Under different N fertilization treatments, *stu-miR482a-5p* exhibited higher levels of expression in two periods and two tissues in the Atlantic variety.

The expression of *stu-miR166c-5p_L-3* and *stu-miR3627-3p* was lower under both types of N stress, in two potatoes, at two periods, and in two tissues. *stu-miR156a_L-1* was expressed at lower levels in the seedling stage of two kinds of non-N treated potatoes, in the seedling stage of the Atlantic variety under both types of N treatments, and non-N treated Atlantic roots at the budding and seedling stages. The expression of *stu-miR172b-5p* in two tissues at two periods in the Yanshu4 variety was lower under both N treatments.

### Prediction and enrichment analysis of target genes corresponding to miRNAs

#### Target gene prediction of differential miRNAs

Target genes for miRNAs with significant differences were predicted using psRobot (v1.2) [16]. A target penalty strategy based on plants helped us to predict targets (the default threshold score was ≤ 2.5). We performed target gene prediction for differential miRNAs and extracted information regarding the target genes corresponding to miRNAs and obtained annotation information for the target genes using the GO and KEGG databases.

### Target gene enrichment analysis of differential miRNAs

**Fig. 3:**
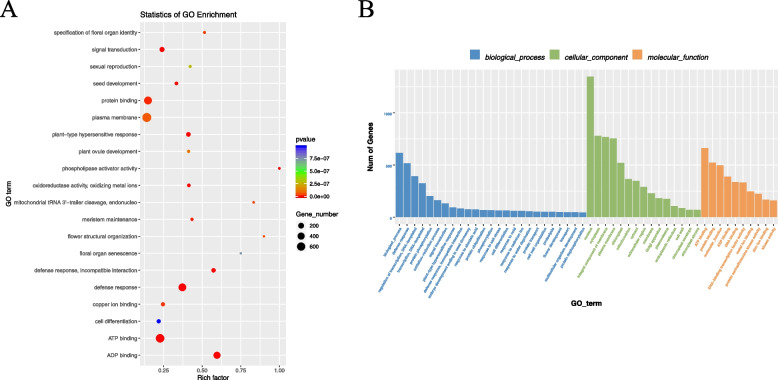
GO enrichment analysis of target genes corresponding to differential miRNAs

Enrichment analysis can be classified into two parts, i.e., GO functional annotation (Fig. 2), and KEGG pathway functional annotation (Fig. 3).

Our analysis revealed that *stu-miR396-5p* was involved in pathways associated with N metabolism, carbon metabolism, pyruvate metabolism, alanine, aspartate and glutamate metabolism, phytohormone biosynthesis, amino acid biosynthesis, and zeatin biosynthesis. Sixty-five target genes were predicted for *stu-miR396-5p*. *stu-miR482a-5p* was mainly involved in folate biosynthesis, glycerophosphate metabolism, and gluconate metabolism, and 12 target genes were predicted. The sequencing and analysis of *stu-miR156a_L-1* revealed that it was mainly involved in biological processes and pathways associated with N metabolism, endoplasmic reticulum-related processes, RNA degradation, and ubiquitin-mediated protein degradation; 50 target genes were predicted. *stu-miR172b-5p* was mainly involved in pathways associated with starch and sucrose metabolism and arginine metabolism. *stu-miR482a-5p* was mainly involved in folate biosynthesis, glycerophosphate metabolism, and gluconate metabolism, and 12 target genes were predicted. The sequencing and analysis of *stu-miR156a_L-1* revealed that it was mainly involved in biological processes and metabolic pathways associated with N metabolism, endoplasmic reticulum-related processes, and proline metabolism; 13 target genes were predicted. An analysis of *stu-miR827-3*p revealed that it was mainly involved in biological processes such as amino acid biosynthesis and phytohormone signaling; 31 target genes were predicted. An analysis of *stu-miR408b-3p_R-1* revealed that it was mainly involved in metabolic pathways and biological processes such as N metabolism, carbon metabolism, ascorbic acid, and uric acid metabolism; 9 target genes were predicted. *stu-miR3627-3p* was found to be mainly involved in RNA transport, carbon metabolism, and other pathways, and 3 target genes were predicted. *stu-miR482a-3*p analysis revealed that the metabolic pathways were mainly involved in endocytosis, plant-pathogen interactions, and ubiquitin-mediated proteolytic metabolism; 14 target genes were predicted. *stu-miR827-5p* analysis revealed that it was mainly involved in amino acid sugar and nucleotide sugar metabolism, plant-pathogen interactions, and glutathione metabolism-associated pathways; 9 target genes were predicted. *stu-miR6022-p3_7* was found to be mainly involved in metabolic pathways and biological processes such as N metabolism, amino acid biosynthesis, and thiamine metabolism; 8 target genes were predicted. An analysis of *stu-miR398a-5p* revealed that it was mainly involved in metabolic pathways such as peroxisome, homologous recombination, and photosynthesis, and 4 target genes were predicted. *stu-miR166c-5p_L-3* was mainly involved in metabolic pathways such as pyruvate metabolism, glycolysis/gluconeogenesis, and valine, leucine, and isoleucine degradation, and 11 target genes were predicted.

GO has three ontologies that describe the molecular functions, cellular components, and associated biological processes, respectively (see Additional file 1, Table 4 for details).

GO enrichment analysis was performed to identify the top 20 annotations that were closely related to N metabolism. These included GO0003674 (molecular function), GO0003700 (DNA-binding transcription factor activity), GO0004674 (protein serine/threonine kinase activity), GO0005634 (nucleus), and GO0006355 (DNA template, transcriptional regulation). The results showed that the most annotated sequences were associated with the plasma membrane (GO0005886), defense response (GO0006952), and ATP binding (GO0005524). GO enrichment analysis of target genes corresponding to differential miRNAs showed that sequences associated with the defense response were the most annotated among those associated with the 25 biological processes, followed by biological processes. Sequences associated with the nucleus were the most annotated among the 15 cellular components, followed by the plasma membrane, and the 10 most annotated sequences were associated with molecular functions such as ATP binding, followed by protein binding. The top 20 annotations were closely related to N metabolism; these included KO00910 (N metabolism), KO01200 (carbon metabolism), KO04146 (peroxisome), KO00970 (amino acid biosynthesis), KO04075 (phytohormone signaling), KO04626, KO03040 (shear bodies), and KO04075 (phytohormone signaling), and were enriched for KEGG analysis. KEGG enrichment analysis showed that target genes were mainly enriched in phytopathogenic interactions (KO04626) and phytohormone signaling (KO04075).

### Validation of differential miRNAs using qRT-PCR

#### Differential expression of miRNAs exposed to the same N treatment

**Fig. 4:**
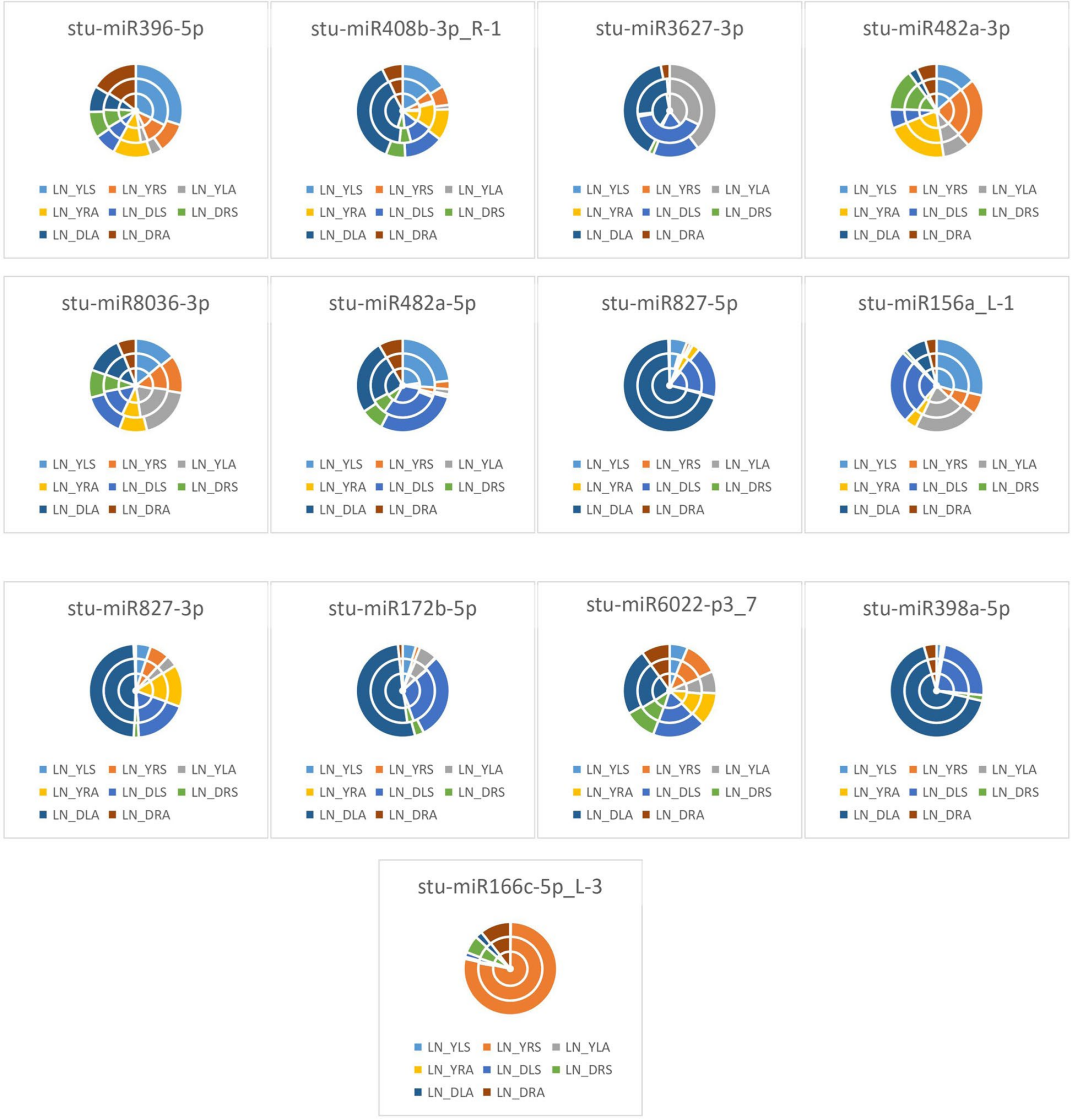
Differential miRNA expression patterns under no N treatment HN: excess N application; LN: no N application; Y: Yanshu4; D: Atlantic; S: seedling stage; A: budding stage

As seen from the expression of differential miRNAs under the unappliednon-N-treatment in Fig. 4, LN_YLS processing conditions accounted for the largest proportion of differential expression of *stu-miR396-5p* and *stu-miR156a_L-1*, indicating that *stu-miR396-5p* and *stu-miR156a_L-1* were most significantly expressed for LN_YLS under treatment; in LN_DLA, *stu-miR408b-3p_R-1*, *stu-miR3627-3p*, *stu-miR827-5p*, *stu-miR827-3p*, *stu-miR172b-5p*, *stu-miR6022-p3_7*, and *stu-miR398a-5p* accounted for the largest percentage differences, indicating that the differential expression of these miRNAs was the highest under DLA treatment. LN_YRS accounted for the largest proportion of differential expression of *stu-miR482a-3p*, indicating that *stu-miR482a-3p* was differentially expressed for LN_YRS under treatment; LN_YLA accounted for the largest proportion of differential expression of *stu-miR8036-3p*, indicating that the differential expression of *stu-miR8036-3p* was significant for LN_YLA under treatment; LN_DLS accounted for the largest share in the differential expression of *stu-miR482a-5p*, indicating that *stu-miR482a-5p* was differentially expressed in LN_DLS under treatment conditions. LN_YRS was the most represented in the differential expression of *stu-miR166c-5p_L-3*, indicating that the differential expression of *stu-miR166c-5p_L-3* was significant in LN_YRS under treatment conditions.

**Fig. 5:**
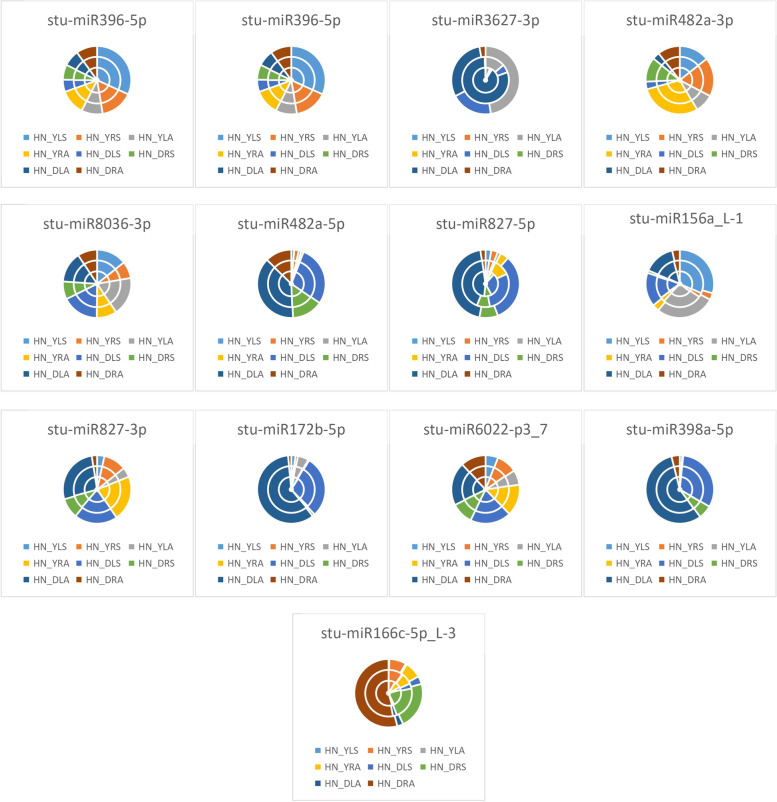
Differential miRNA expression patterns under excessive N treatment HN: excess N application; LN: no N application; Y: Yanshu4; D: Atlantic; S: seedling

As shown in Fig. 5, the expression of differential miRNAs under excessive N treatment conditions showed that HN_YLS had the largest share in the differential expression of *stu-miR396-5p*, *stu-miR408b-3p_R-1*, and *stu-miR156a_L-1*, indicating that the differential expression of *stu-miR396-5p*, *stu-miR408b-3p_R-1*, and *stu-miR156a_L-1* was most significant under HN_YLS treatment conditions; the largest proportion of differential expression of *stu-miR3627-3p*, *stu-miR482a-5p*, *stu-miR827-5p*, *stu-miR172b-5p*, and *stu-miR398a-5p* was observed under HN_DLA treatment, indicating that the differential expression of these miRNAs was most significant under HN_DLA treatment conditions; HN_YRA accounted for the largest proportion of differential expression of *stu-miR482a-3p* and *stu-miR827-3p*, indicating that *stu-miR482a-3p* and *stu-miR827-3p* were differentially expressed under HN_YRA treatment conditions; HN_YLA accounted for the significant expression of *stu-miR8036-3p*, which exhibited the largest proportion of differential expression, indicating that *stu-miR8036-3p* was differentially expressed under HN_YLA treatment conditions; HN_DLS accounted for the largest proportion of differential expression of *stu-miR6022-p3_7*, indicating that *stu-miR6022-p3_7* was differentially expressed under HN_DLS treatment conditions; HN_ DRA accounted for the highest level of differential expression of *stu-miR166c-5p_L-3*, indicating that the differential expression of *stu-miR166c-5p_L-3* was significant under HN_DRA treatment conditions.

### Expression of differential miRNAs under different N treatments

**Fig. 6:**
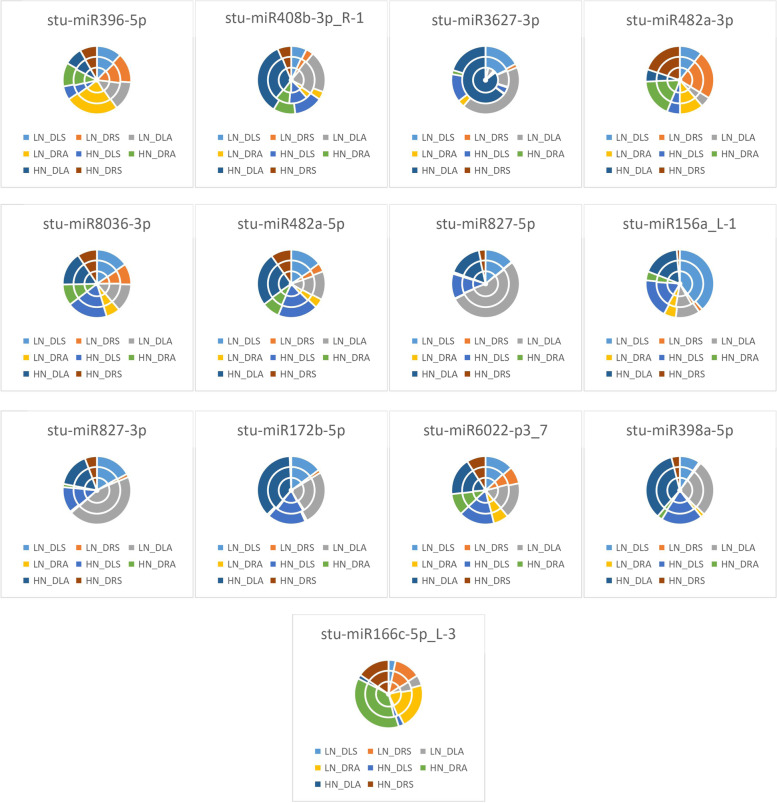
Differential miRNA expression patterns in the Atlantic variety HN: excess N application; LN: no N application; Y: Yanshu4; D: Atlantic; S: seedling stage; A: budding stage

As seen from the expression of differential miRNAs in the Atlantic variety in Fig. 6, LN_DRA accounted for the largest proportion of the differences in the expression of *stu-miR396-5p*, which indicated that the differential expression of *stu-miR396-5p* was the most significant in LN_DRA under treatment; HN_DLA accounted for the largest proportion of the differences in the expression of *stu-miR408b-3p_R-1*, *stu-miR3627-3p*, *stu-miR482a-5p*, *stu-miR172b-5p*, and *stu-miR398a-5p*, indicating that these differential miRNAs were most significantly expressed in HN_DLA under treatment; LN_DRS accounted for the largest percentage of differential expression of *stu-miR482a-3p*, indicating that *stu-miR482a- 3p* was most significantly expressed in LN_DRS under treatment; HN_DLS accounted for the largest proportion of differences in the expression of *stu-miR8036-3p*, indicating that *stu-miR8036-3p* was most significantly expressed in HN_DLS under treatment; LN_DLA had the largest proportion of differences in the expression of *stu-miR827-5p*, *stu-miR827-3p*, and *stu-miR6022-p3_7*, indicating that *stu-miR827-5p*, *stu-miR827-3p*, and *stu-miR6022-p3_7* were most significantly differentially expressed in LN_DLA under treatment; LN_DLS accounted for most significant expression of *stu-miR156a_L-1*, indicating that the differential expression of *stu-miR156a_L-1* was most significant in LN_DLS under treatment; HN_DRA accounted for the most significant differential expression of *stu-miR166c-5p_L-3*, indicating that the differential expression of *stu-miR166c-5p_L-3* was most significant in HN_DRA under treatment.

**Fig. 7:**
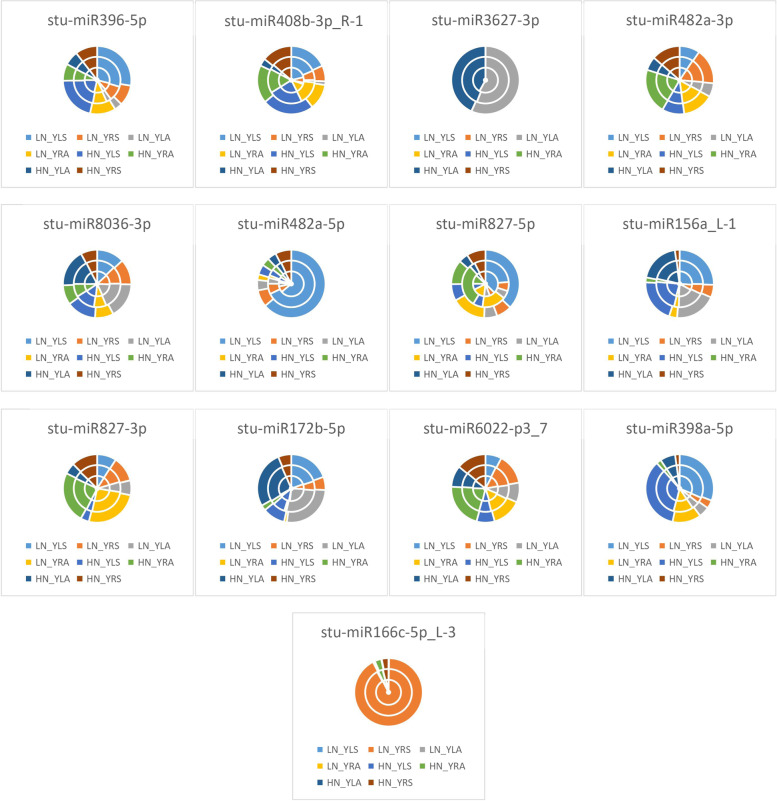
Differential miRNA expression patterns in the Yanshu4 variety HN: excess N application; LN: no N application; Y: Yanshu4; D: Atlantic; S: seedling stage; A: budding stage

As shown in Fig. 7, in Yanshu4, LN_YLS accounted for the largest differences in expression in *stu-miR396-5p*, *stu-miR482a-5p*, *stu-miR827-5p*, and *stu-miR156a_L-1*, indicating that the differential expression of *stu-miR396-5p*, *stu-miR482a-5p*, *stu-miR827-5p*, and *stu-miR156a_L-1* was most significant in LN_YLS under treatment; HN_YLS accounted for the largest proportion of differences in the expression of *stu-miR408b-3p_R-1* and *stu-miR398a-5p*, indicating that the expression of *stu-miR408b-3p_R-1* and *stu-miR398a-5p* was most significant in HN_YLS under treatment; HN_YLS accounted for the largest differences in expression of *stu-miR408b-3p_R-1* and *stu-miR398a-5p*, indicating that *stu-miR408b-3p_R-1* and *stu-miR398a-5p* were most significantly expressed in HN_YLS under treatment; LN_YLA accounted for the largest difference in expression of *stu-miR3627-3p*, indicating that *stu-miR3627-3p* was most significantly expressed in LN_YLA under treatment; HN_YRA accounted for the largest difference in expression of *stu-miR482a-3p* and *stu-miR6022-p3_7*, indicating that the differential expression of *stu-miR482a-3p* and *stu-miR6022-p3_7* was most significant in HN_YRA under treatment; HN_YLA accounted for the largest proportion of differences in expression of *stu-miR8036-3p* and *stu-miR172b-5p*, indicating that the differential expression of *stu-miR8036-3p* and *stu-miR172b-5p* by HN_YLA was most significant under treatment; LN_YRA accounted for the largest proportion of differences in the expression of *stu-miR827-3p*, indicating that the differential expression of *stu-miR827-3p* was most significant in LN_YRA under treatment.

Under both types of N stress in the Yanshu4 variety, *stu-miR396-5p* was down-regulated in the seedling-stage and budding-stage roots and up-regulated in the seedling-stage and budding-stage leaves. *stu-miR408b-3p_R-1* and *stu-miR482a-3p* were up-regulated in the seedling-stage and budding-stage roots and leaves. *stu-miR8036-3p* was up-regulated in the seedling stage and roots and up-regulated in the budding-stage leaves and roots. The expression of *stu-miR482a-5p* was up-regulated in the seedling stage and roots. *stu-miR156a_L-1* was down-regulated in the seedling stage and roots and the budding-stage roots. *stu-miR172b-5p* expression was up-regulated in the seedling-stage and budding-stage roots and down-regulated in the seedling stage. *stu-miR6022-p3_7* expression was up-regulated in the seedling stage. *stu-miR398a-5p* expression was up-regulated in the seedling-stage and budding-stage leaves and down-regulated in the seedling-stage and budding-stage roots. The expression of *stu-miR166c-5p_L-3* was down-regulated in the seedling stage and roots and up-regulated in leaves and roots in the budding-stage.

**Fig. 8:**
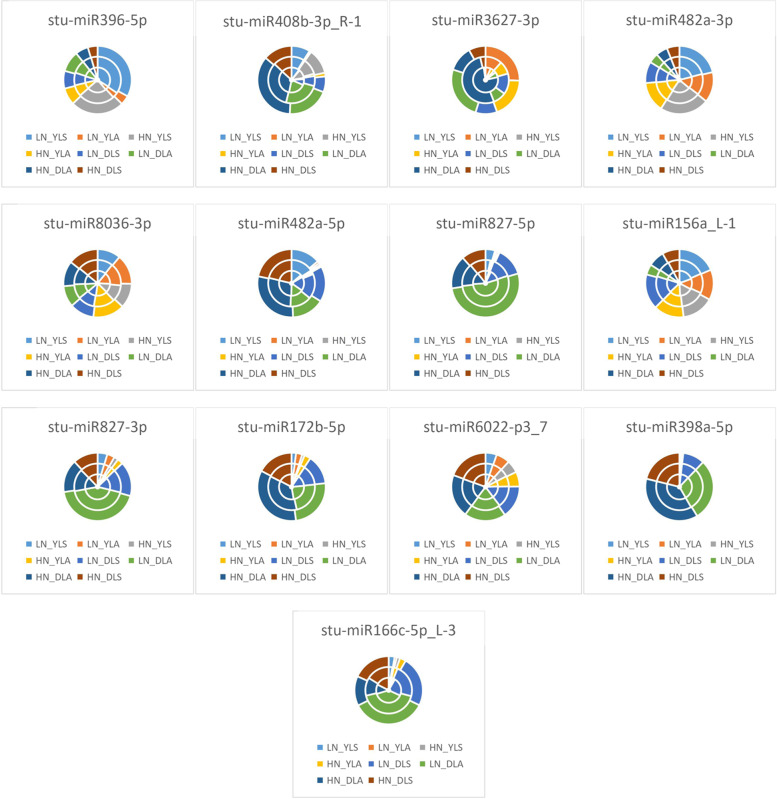
Differential miRNA expression patterns in the leaves of two potato varieties HN: excess N application; LN: no N application; Y: Yanshu4; D: Atlantic; S: seedling stage; A: budding stage

As seen from the expression of differential miRNAs in leaves of the two potatoes in Fig. 8, LN_YLS accounted for the largest proportion of differences in the expression of *stu-miR396-5p*, indicating that *stu-miR396-5p* was most significantly expressed by LN_YLS under treatment; HN_DLA accounted for the largest proportion of the differences in the expression of *stu-miR408b-3p_R-1*, *stu-miR3627-3p*, *stu-miR482a-5p*, *stu-miR172b-5p*, and *stu-miR398a-5p*, indicating that the differential expression of the above miRNAs were most significant in HN_DLA under treatment; HN_YLS accounted for the most significant differential expression of *stu-miR482a-3p*, indicating that *stu-miR482a-3p* was most significantly expressed by HN_YLS under treatment; LN_YLA accounted for the largest proportion of differences in the expression of *stu-miR8036-3p*, indicating that *stu-miR8036-3p* was most significantly expressed by LN_YLA under treatment; LN_DLA accounted for the most significant expression of *stu-miR827-5p*, *stu-miR827-3p*, *stu-miR6022-p3_7*, and *stu-miR166c-5p_L-3* accounted for the largest expression differences, indicating that the above miRNAs were most significantly expressed differentially in LN_DLA under treatment.

**Fig. 9:**
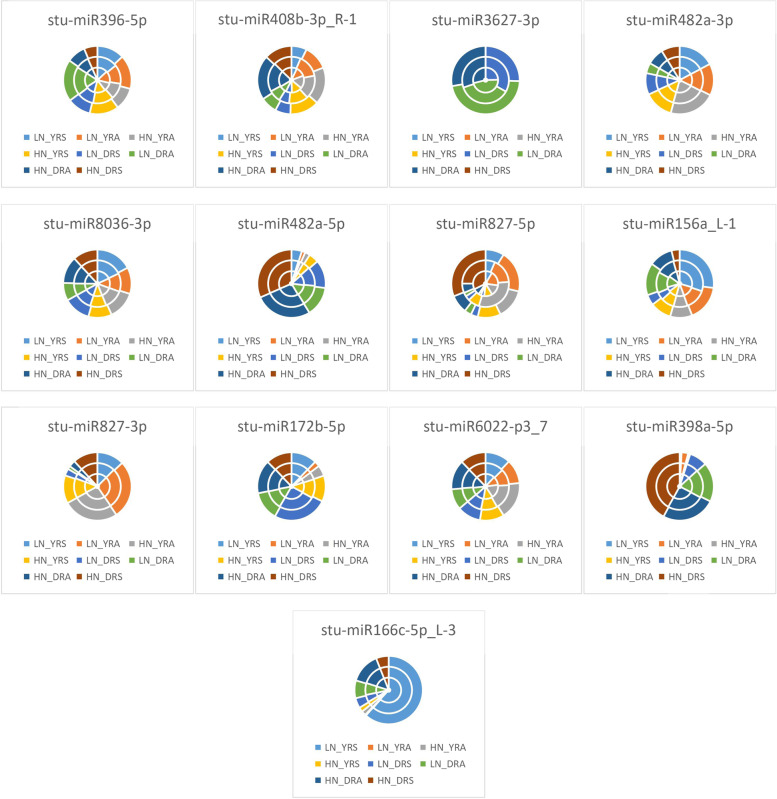
Differential miRNA expression patterns in the roots of two potato varieties HN: excess N application; LN: no N application; Y: Yanshu4; D: Atlantic; S: seedling stage; A: budding stage

As shown in Fig. 9, the expression of differential miRNAs in two potato roots showed that LN_DRA accounted for the largest proportion of the differences in the expression of *stu-miR396-5p* and *stu-miR3627-3p*, indicating that the differential expression of *stu-miR396-5p* and *stu-miR3627-3p* was the highest in LN_DRA under treatment. HN_DRA accounted for the largest percentage of differences in the expression of *stu-miR408b-3p_R-1* and *stu-miR156a_L-1*, indicating that the differential expression of *stu-miR408b-3p_R-1* and *stu-miR156a_L-1* was most significant in HN_DRA under treatment; HN_YRA was expressed differentially in *stu-miR482a-3p* and *stu-miR6022-p3_7*, indicating that the differential expression of *stu-miR482a-3p* and *stu-miR6022-p3_7* was most significantly in HN_YRA under treatment; LN_YRS accounted for the most significant differential expression of *stu-miR8036-3p* and *stu-miR166c-5p_L-3*, indicating that the differential expression of *stu-miR8036-3p* and *stu-miR166c-5p_L-3* was most significant in LN_YRS under treatment; HN_DRS accounted for the largest percentage of differences in the expression of *stu-miR482a-5p*, *stu-miR827-5p*, and *stu-miR398a-5p*, indicating that the differential expression of the above miRNAs by HN_DRS was most significant under treatment; LN_DRS accounted for the largest percentage of differences in expression of *stu-miR827-3p* and *stu-miR172b-5p*, indicating that *stu-miR827-3p* and *stu-miR172b-5p* were most significantly expressed by LN_DRS under treatment; HN_YRA accounted for the most significant differences in the expression of *stu-miR6022-p3_7*, indicating that *stu-miR6022-p3_7* was most significantly expressed differentially by HN_YRA under treatment.

**Fig. 10:**
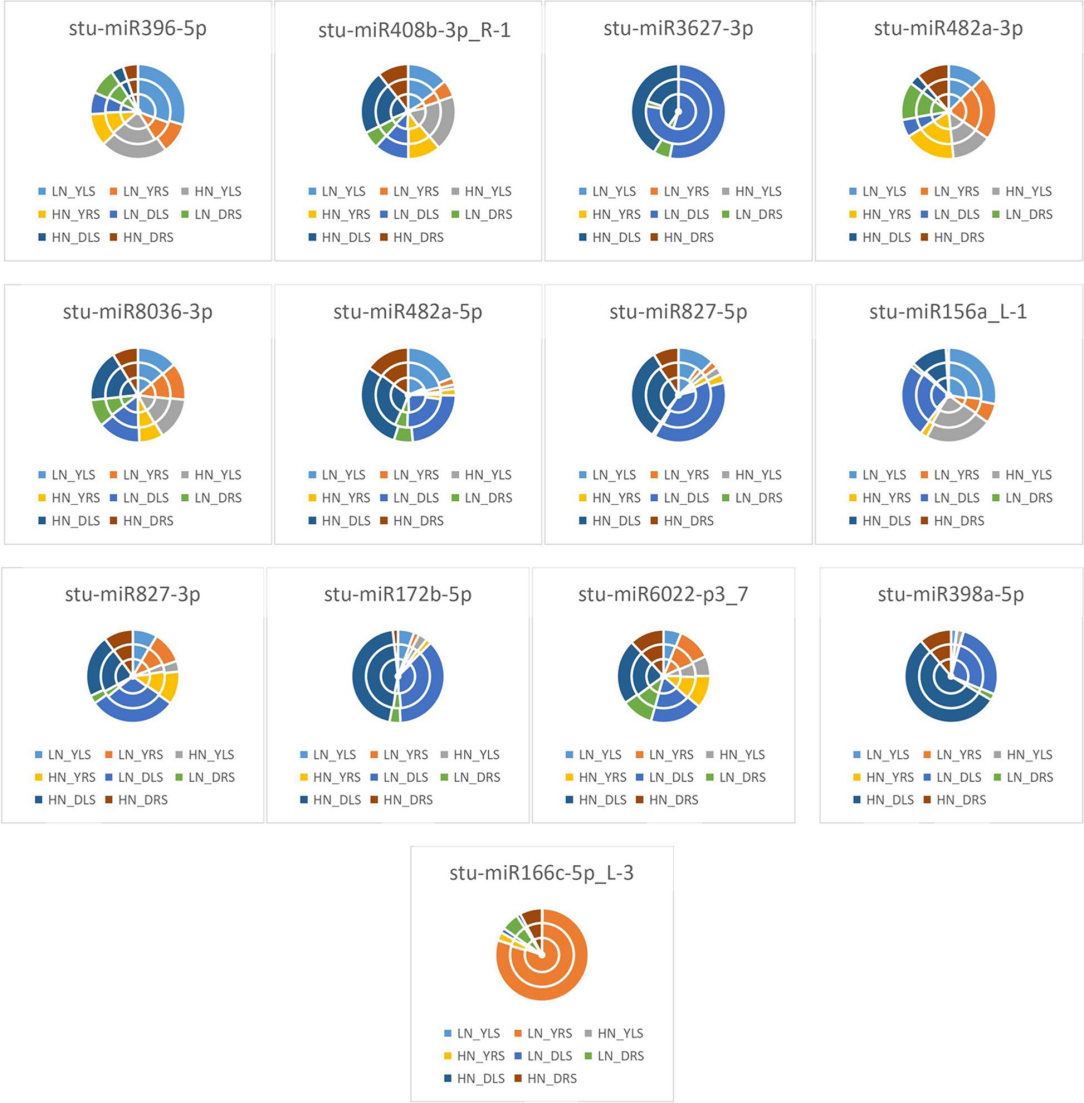
Differential miRNA expression patterns at the seedling stage in the two potato varieties HN: excess N application; LN: no N application; Y: Yanshu4; D: Atlantic; S: seedling stage; A: budding stage

As seen from the expression of differential miRNAs in the two potato seedlings in Fig. 10, LN_YLS accounted for the largest difference in the expression of *stu-miR396-5p*, indicating that the differential expression of *stu-miR396-5p* was most significant in LN_YLS under treatment; HN_DLA accounted for the differential expression of *stu-miR408b-3p_R-1*, *stu-miR8036-3p*, *stu-miR482a-5p*, *stu-miR172b-5p*, *stu-miR6022-p3_7*, *stu-miR398a-5p*, and *stu-miR166c-5p_L-3*, indicating that the above miRNAs were most significantly expressed differentially in HN_DLA under treatment; LN_DLS accounted for the most significant expression of *stu-miR3627-3p*, *stu-miR827-5p*, and *stu-miR827-3p*, indicating that *stu-miR3627-3p*, *stu-miR827-5p*, and *stu-miR827-3p* were most differentially expressed in LN_DLS under treatment; HN_YRS accounted for the most significant difference in the expression of *stu-miR482a-3p*, indicating that the differential expression of *stu-miR482a-3p* was most significant in HN_YRS under treatment.

**Fig. 11:**
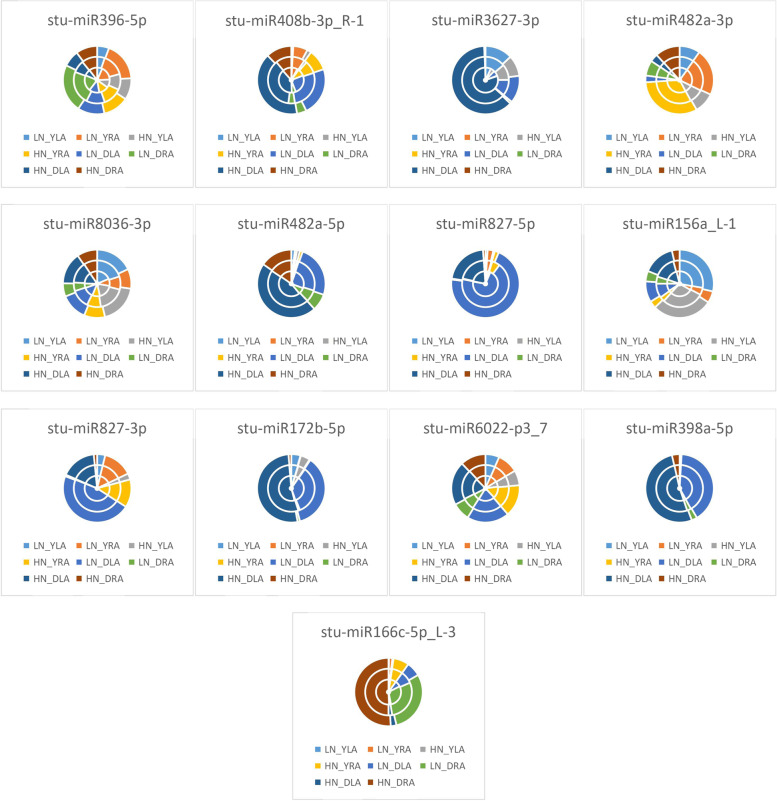
Differential miRNA expression patterns in the two potato varieties at the budding stage HN: excess N application; LN: no N application; Y: Yanshu4; D: Atlantic; S: seedling stage; A: budding stage

As seen in Fig. 11, the expression of differential miRNAs in two potato varieties at the budding stage in HN_YRS accounted for the largest percentage difference in the expression of *stu-miR396-5p*, indicating that *stu-miR396-5p* was most significantly expressed in HN_YRS under treatment; HN_DLA accounted for the largest percentage difference in the expression of *stu-miR408b-3p_R-1*, *stu-miR3627-3p*, *stu-miR482a-5p*, and *stu-miR172b-5p*, indicating that the differential expression of these miRNAs was the most significant in HN_DLA under treatment; HN_YRA accounted for the most significant expression of *stu-miR482a-3p*, indicating that *stu-miR482a-3p* was most significantly expressed by HN_YRA under treatment; HN_YLA exhibited the largest proportion of differences in the expression of *stu-miR8036-3p*, indicating that *stu-miR8036-3p* was most significantly expressed in HN_YLA under treatment; LN_DLA exhibited the largest proportion of differences in the expression of *stu-miR827-5p*, *stu-miR827-3p*, *stu-miR6022-p3_7*, and *stu-miR398a-5p*, indicating that the above miRNAs were most differentially expressed in LN_DLA under treatment; LN_YLA accounted for the most differential expression of *stu-miR156a_L-1*, indicating that the differential expression of *stu-miR156a_L-1* was the highest for LN_YLA under treatment; HN_DRA accounted for the largest proportion of differences in the expression of *stu-miR166c-5p_L-3*, indicating that the differential expression of *stu-miR166c-5p_L-3* was the most significant in HN_DRA under treatment.

### Analysis of traits, target genes, and differential miRNAs

#### Analysis of the relationship between traits and target genes

**Fig. 12:**
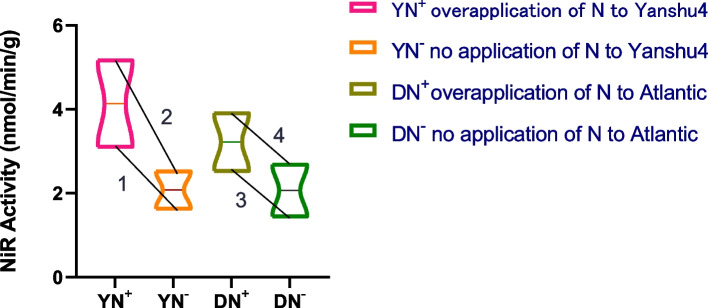
Analysis of nitrite reductase (NiR) activity of N-efficient and N-inefficient potato plants under N stress. 1 and 3, the seedling stage; 2 and 4 the budding stage

As shown in Fig. 12, the NiR activities in the Yanshu4 and Atlantic varieties showed a significant upward trend with an increase in the N levels applied from the seedling stage to the germination stage, with either excess N application or no N application. This indicated that the NiR activities in the two kinds of potatoes were positively correlated with both types of N stress, i.e., the *NiR* gene in the two kinds of potatoes was highly responsive to N stress.

**Fig. 13:**
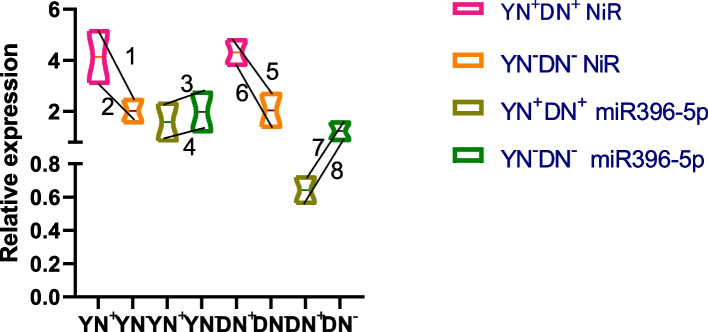
Relative expression analysis of the N-efficient Yanshu4 variety, N-inefficient, nitrite reductase-expressing Atlantic variety, and miR396-5p under N stress YN + : N over-application in the Yanshu4 variety; YN-: No N application in the Yanshu4 variety; DN + : N over-application in the Atlantic variety; DN-: No N application in the Atlantic variety; 2, 3, 5, and 7: the seedling stage; 1, 4, 6, and 8: the budding stage

The analysis of target genes and their corresponding differential miRNAs is shown in Fig. 13. In the Yanshu4 and Atlantic varieties, the relative expression of *NiR* from the seedling stage to the budding stage showed a significant upward trend from the absence of N application to excessive N application. *stu-miR396-5p* expression decreased significantly from the seedling stage to the budding stage from the absence of N application to excessive N application, especially in the Yanshu4 variety.

The relative expression of *NiR* in the two kinds of potatoes showed a significant upward trend from excessive N application to the absence of N application. The relative expression of *stu-miR396-5p* in the two potato varieties showed a significant downward trend from excessive N application to the absence of N application. The expression of *NiR* in the two potato varieties was positively correlated with N application, while the relative expression of *stu-miR396-5p* was negatively correlated with N application.

**Fig. 14:**
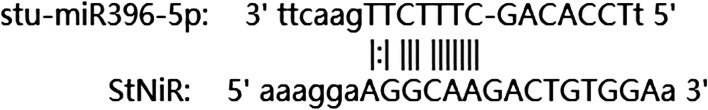
Prediction of the binding of stu-miR396-5p with the StNiR binding site

The prediction of the binding site of *stu-miR396-5p* to *StNiR* was performed using the online URL and the predicted results are shown in Fig. 14. There may be a binding site between *stu-miR396-5p* and *StNiR*, and there may be a splicing relationship between the 8^th^ base of *stu-miR396-5p* and the 8^th^ base of *StNiR* from the 5' end of *stu-miR396-5p*.

**Fig. 15:**
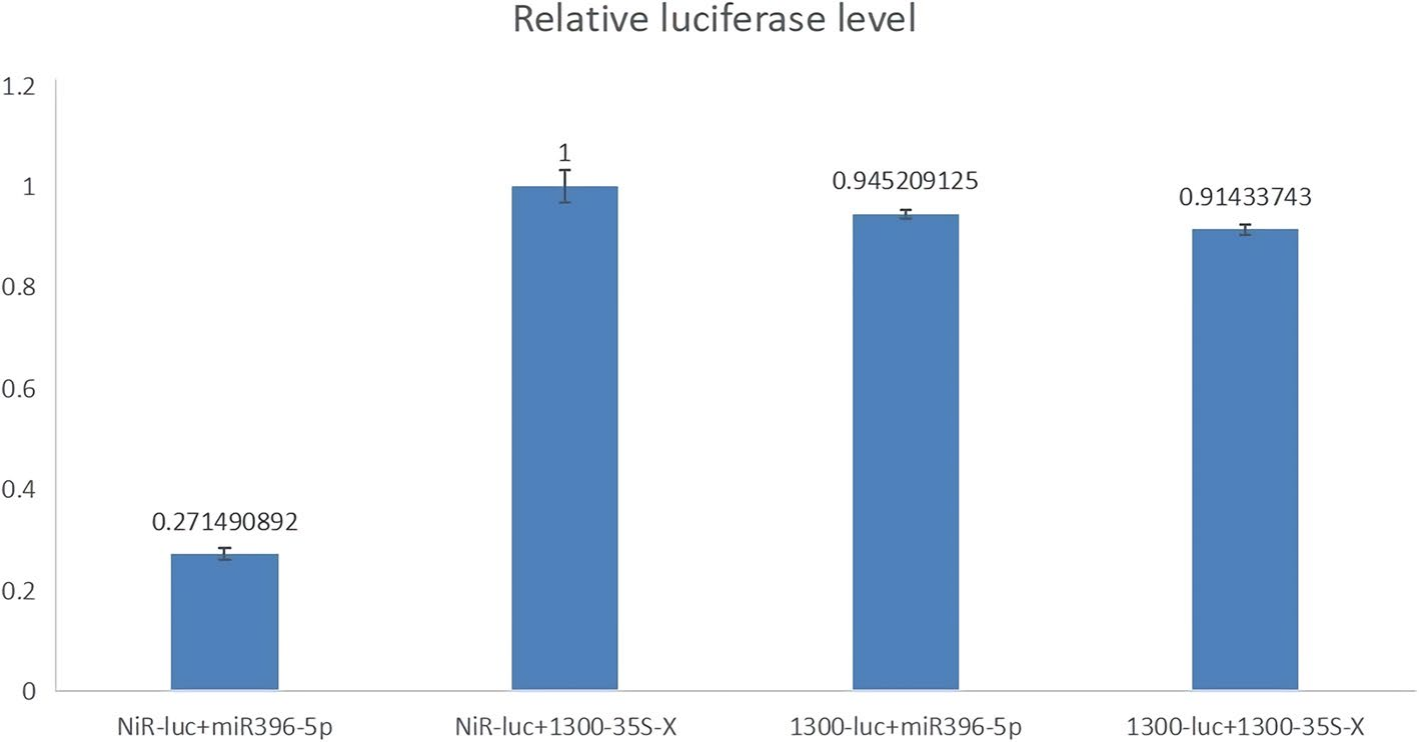
Validation of the binding site of stu-miR396-5p to StNiR

As shown in Fig. 15, the binding ratio of NiR-luc to miR396-5p was 0.2715; the binding ratio of NiR-luc to 1300-35S-X was 1; the binding ratio of 1300-luc to NiR-luc was 0.9452; and the binding ratio of 1300-luc to NiR-luc was 0.9452. The binding ratio to 1300-35S-X was 0.9143; hence, it was confirmed that miR396-5p binds to the NiR sequence and causes product degradation. Therefore, it can be confirmed that *stu-miR396-5p* could bind in a targeted manner with *StNiR*.

## Discussion

### Analysis of miRNA expression under N stress

The continuous developments in sequencing technology have resulted in its increasingly extensive application in recent years. Studies involving the use of Illumina sequencing technology have shown that there are differences in the aluminum tolerance of miRNAs between *Ailanthus sinensis* and *Ailanthus macrophylla* roots. miR160 promotes adventitious and lateral root development; miR3627 results in an increase in citric acid secretion; miR3627 and miR482 control the alternative glycolytic pathway and TCA cycle in a flexible manner; miR172 regulates miRNA metabolism flexibly. miR160 was found to affect root development in citrus plants, and *cas-miR5139* and *csi-miR1210*5 played important roles in the tolerance to aluminum in citrus plants via the regulation of cell wall components [17]. The study by Fische on the regulation of miRNA expression in plants showed that *miR396* expression was down-regulated under N-deficient conditions [18]. Trindade et al. have revealed that miR396 facilitated the generation of a response to water stress in alfalfa [19]. Our miRNA sequencing results showed that the expression levels of *stu-miR396-5p*, *stu-miR8036-3p*, and *stu-miR482a-3p* were significantly different in the over- and under-fertilized seedlings, roots, and leaves in the germination stage in the Yanshu4 and Atlantic varieties.

### Expression analysis of differential miRNAs in two potato cultivars, root and leaf tissues, and two growth stages under two N stress conditions

#### Expression analysis of miR396

Numerous studies have shown that different miRNAs are involved in the generation of responses to different stress conditions and play extremely important roles in plant growth. miR396 is directly related to the metabolic regulation, growth, and development of plants [20], and is expressed upon exposure to water stress, temperature stress, salt stress, and during processes such as oxidative processes, fatty acid metabolism, root tip growth and development [21], and bacterial infections [22]. In addition, miR396-GRF plays an important regulatory role in the response to different types of N stress, and the expression of *LsaGRF*s in lettuce with *LSA-miR396* can regulate leaf growth via the cleavage of complementary sequences [23]. We found that the differentially expressed *stu-miR396-5p* was highly responsive to N stress, with *stu-miR396-5p* being down-regulated in the Atlantic variety in both the seedling stage and roots, and in the budding-stage leaves and roots, and *stu-miR396-5p* being down-regulated in the seedling-stage and budding-stage roots, and up-regulated in the seedling-stage and budding-stage leaves in the Yanshu4 variety. A study by Fischer [24] on the regulation of miRNA expression in plants found that the expression of miR396 was also down-regulated under N deficient conditions [25].

#### Expression analysis of miR156

Studies have shown that miR156 expression is upregulated in plants under N deficient conditions. miR156 has also been found to play an important role in many metabolic pathways in the potato plant, and it has been shown that miR156 inhibits potato tuber formation [26]. miR156e regulates potato tuber development by regulating its target gene *StPTB6 *[27]. It has been shown that the expression level of potato miR156 is also regulated during the photoperiod [28]. miR156/157 targets SPL transcription factors, thereby regulating the polarity of flowering organs in the potato plant [29]. In this study, we found that *stu-miR156a_L-1* was down-regulated in the seedling stage and roots, and in roots in the budding stage under two types of N stress in the Yanshu4 variety. In the Atlantic variety, *stu-miR156a_L-1* was down-regulated in the seedling stage and roots, and up-regulated in the seedling stage and leaves at the budding stage under both types of N stress, and the expression of both potatoes was significantly higher in leaves treated with excess N than in leaves not treated with N. It is assumed that *stu-miR156_L-1* was up-regulated under N stress. The corresponding target gene was *NRT2.5*, a key enzyme involved in the N metabolism pathway, and a previous study on the *NRT* family revealed that family members play an active role in the N metabolism pathway when the N level is sufficient, while N deficiency induces stress in potato [30]. Thus, we further speculate that *stu-miR156a_L-1* may have a regulatory relationship with its target gene *NRT2.5*, which facilitates the regulation of the N metabolic pathway in potato; however, the exact regulatory mechanism needs to be explored further.

#### Expression analysis of miR482

Notably, pathogenic infections were effectively suppressed in potato [31], tomato [32], and cotton [33] plants through the inhibition of miR482. We found that the expression of *stu-miR482a-5p* and *stu-miR482a-3p* was up-regulated with an increase in N application under both types of N stress, which could indicate that miR482a-5p is sensitive to different N treatments. *stu-miR482a-3p* was up-regulated in the seedling stage and roots and leaves in the budding stage under both types of N stress. The expression of *stu-miR482a-3p* was up-regulated in the seedling stage and roots and leaves at the budding stage under both types of N stress. In the Atlantic variety, *stu-miR482a-3p* was down-regulated in the seedling stage and roots and up-regulated in leaves and roots in the budding stage under both types of N stress. *stu-miR482a-5p* was up-regulated in the seedling stage and roots of the Yanshu4 variety and up-regulated in both the seedling stage and roots and leaves in the budding stage in the Atlantic variety. We tentatively identified the role of miR482a against a novel abiotic stress, but its specific functions need to be investigated further.

#### Expression analysis of miR172

miR172 is reportedly involved in flower and tuber induction signaling pathways in potato plants and helps visualize the clear link between solute transport and flowering and tuber induction [34]. It has also been shown that tomato miR172 targets the APETALA2 transcription factor SlAP2a to regulate fruit ripening [35]. Ferdous found that under conditions of water stress in barley plants, miR172, miR396a, and miR396c regulated *P5CS* expression, which in turn regulates proline accumulation and provides molecular evidence regarding the process of drought tolerance in potato plants [36]. It was also found that miR172 was significantly expressed in the potato tuber developmental stages [37]. In this study, *stu-miR172b-5p* was found to be expressed at higher levels in the budding-stage roots of the Atlantic variety than in leaves subjected to excessive N treatment. *stu-miR172b-5p* was up-regulated in the seedling-stage and budding-stage leaves and roots of the Atlantic variety and down-regulated in the seedling stage. In Yanshu4, *stu-miR172b-5p* was up-regulated in the seedling-stage and budding-stage roots, and down-regulated in the seedling stage; thus, it is highly responsive to N stress. Our findings are similar to the findings obtained in previous studies, but the functions of these miRNAs need to be investigated further.

#### Expression analysis of miR827

It has been suggested that miR827 plays a key role in adaptation to drought tolerance in barley, and miR827 strongly induces Pi starvation through the aboveground area and roots [38]. We also found a more significant difference between *stu-miR827-3p* and *stu-miR827-5p* in both types of potato plants subjected to different N treatments. In Yanshu4, *stu-miR827-5p* was down-regulated in the seedling stage. *stu-miR827-3p* was down-regulated in the seedling stage, roots, and budding-stage roots, and was up-regulated in the seedling stage. In the Atlantic variety, *stu-miR827-5p* was up-regulated in the seedling- and budding-stage roots and down-regulated in the seedling- and budding-stage leaves. *stu-miR827-3p* was down-regulated in the seedling- and budding-stage leaves and up-regulated in the seedling- and budding-stage roots. However, we intended to screen for N metabolism-related genes whose target genes do not include key enzymes in the metabolic pathway for N, as this would provide new directions for research.

#### Expression analysis of miR408

miR408 is involved in the regulation of plant growth and development, and responses to stress. miR408 can affect photosynthesis and ultimately promote grain yield by down-regulating target proteins that regulate plastocyanin; this reveals the function of miR408 and its targets in the regulation of plant growth and development and responses of plants to various abiotic and biotic stresses [39]. Previous studies have shown that overexpression of miR408 significantly enhances drought tolerance in chickpea [40], while new studies have shown that the loss of function of miR408 can negatively regulate germination in light-dependent seeds [41]. We found that *stu-miR408b-3p_R-1* was up-regulated in the seedling-stage and budding-stage roots and leaves of the Yanshu4 variety subjected to both types of N stress. In the Atlantic variety, *stu-miR408b-3p_R-1* was up-regulated in both the seedling stage and roots and the budding-stage leaves and roots because of the high responsiveness to N stress. We verified the expression of miR408 from a new abiotic stress perspective, which opens a new avenue for further understanding this ancient and highly conserved miRNA.

#### Expression analysis of miR8036

There are hardly any reports on miR8036. We found that *stu-miR8036-3p* was up-regulated in the Atlantic variety subjected to both types of N stress in both the seedling stage and roots and in the budding-stage leaves and roots; while in the Yanshu4 variety, *stu-miR8036-3p* was up-regulated in the seedling stage and roots and down-regulated in the budding-stage leaves and roots. The specific regulatory mechanism of action is not clear and needs to be explored thoroughly in future studies.

#### Expression analysis of miR398

miRNA398 is considered to have a direct association with the plant stress regulatory network, as it regulates plant responses to oxidative stress, water deficit, salt stress, abscisic acid stress, UV stress, copper and phosphorus deficiencies, high glucose levels, and bacterial infections [12]. It is of great importance in studies focusing on the responses to both biotic and abiotic stresses. It has been suggested that miR398 enhances superoxide dismutase (SOD) activity in wheat roots by regulating the expression of *WRKY*, thus achieving the alleviation of non-induced oxidative toxicity in wheat roots [42]. Recent studies have shown that miR398 can alleviate the symptoms of bamboo mosaic virus infections and mitigate viral accumulation via the regulation of antioxidants [43]. In the present study, we found that *stu-miR398a-5p* was up-regulated when the Atlantic variety was subjected to both types of N stress in the seedling stage and roots and the budding-stage leaves and roots. *stu-miR398a-5p* was up-regulated in the seedling- and budding-stage leaves and down-regulated in the seedling- and budding-stage roots in the Yanshu4 variety. We investigated the relationship between miRNA398 and N stress and have contributed to research on miRNA398.

#### Expression analysis of miR6022

Studies reporting on significant changes in the relative levels of abundance of *Streptococcus* species such as the *Streptococcus habrochetes* subtype in tomato plants under low-temperature stress could indicate the important role played by miR6022 in response to cold stress [44]. It has also been shown that *sly-miR6022* regulates the tomato R gene *Cf-9* at the post-transcriptional level [45]. In the potato plant, miR6022 was found to be involved in the regulation of other miRNAs via the intertwining of different regulatory networks, which revealed how developmental signals, disease symptom development, and stress signals are regulated by each other, and the balance in their levels is, therefore, maintained  [46]. In the present study, we found that *stu-miR6022-p3_7* was up-regulated in the seedling stage under two types of N stress in the Yanshu4 variety. In the Atlantic variety, *stu-miR6022-p3_7* was up-regulated in the seedling stage and roots at the budding stage. This study provides a new research idea based on previous work in this field, as specific regulatory mechanisms need to be investigated further.

#### Expression analysis of miR166

Recent studies found that the *sly-miR166* and *SlyHB* modules are factors that increase susceptibility for ToLCNDV (New Delhi tomato varroa virus) in the tomato plant. *sly-miR166*/*SlyHB* is negatively regulated; hence, the regulation of *sly-miR166* expression can be achieved by regulating *SlyHB*, which in turn regulates the pathogenesis of ToLCNDV [47]. It was found that tomato plants carrying *Slhb15a* with miRNA166 resistance alleles exhibited normal ovule development, and the use of *Slhb15a* and reciprocal regulation of miRNA166 could promote fruit setting at extreme temperatures [48]. We found that when the Yanshu4 variety was subjected to two types of N stress, *stu-miR166c-5p_L-3* was down-regulated in the seedling stage and roots and up-regulated in the budding-stage leaves and roots; and in the Atlantic variety, *stu-miR166c-5p_L-3* was up-regulated in the budding-stage roots and down-regulated in the budding-stage leaves. The specific regulatory mechanisms attributable for these activities need to be investigated further, to understand the role of these miRNAs against biotic and abiotic stresses in plants more thoroughly.

### Differences in the expression of miRNAs, target genes, and traits under N stress

This study showed that the relative expression of *StNiR* showed a significant upward trend with an increase in N application, while the relative expression of *stu-miR396-5p* showed a significant downward trend with increased N application; this was more notable in the Yanshu4 variety. Degradome analysis revealed that the corresponding target gene of *stu-miR396-5p* was *NiR*, and in combination with the findings of a previous study, NiR activity was found to show a significant upward trend from no N application to excessive N application in the Yanshu4 and Atlantic varieties; this indicates a high level of responsiveness to N stress, which is consistent with the findings of Jiao [49]. This study also reported that *stu-miR396-5p* showed a trend of down-regulation from no N application to excessive N application in the Yanshu4 and Atlantic varieties. Therefore, it was suggested that there was a negative correlation between *stu-miR396-5p* and *StNiR* when potato plants were subjected to N stress. Thus, it was hypothesized that these miRNAs might play an important role in the N metabolic pathway of potato plants.

**Fig. 16:**
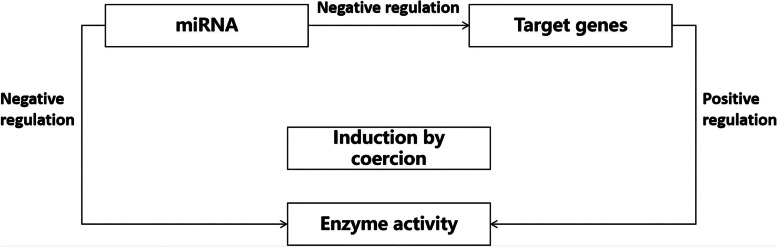
miRNA and target gene regulatory strate

Yi found that the target gene of *sit-miR396*, Type-IV, was functionally enriched mainly when it performed a catalytic role, such as that observed for hydrolase, isomerase, and peroxidase, and a functional study of *sit-miR396* showed that it could effectively regulate the growth of plants [50]. In addition, the rapid validation of rice miRNA target genes via the transient expression of rice protoplasts could enhance the activity of the target gene *OsNF-YA4*; the expression of miR169o was significantly up-regulated, which clarified the regulatory role of *OsNF-YA4* and miR169o in the expression of rice protoplasts [51]. It has also been reported that in tomato plants, the S1COL4 transcription factor plays a negative regulatory role in fruit ripening by regulating *ASC* gene expression, which in turn regulates the ethylene biosynthesis pathway [52]. Therefore, we designed our experimental protocol according to the Fig. 16 shown below, in order to fully explore the specific regulatory role played by *StNiR* and *stu-miR396-5p* in the N metabolism pathway of potato plants.

In summary, our study confirmed the key role of 13 miRNAs in adaptation to two N stresses in potato. The selected *stu-miR396-5p* and its potential target gene *StNiR* are involved in the nitrogen metabolism pathway of potato, and further studies are needed to confirm the specific loci of action of miRNAs and their targets to explore their functions in nitrogen uptake and utilization, so as to breed nitrogen-efficient potato varieties that respond to nitrogen stress without affecting yield.

## Conclusion

miRNA sequencing facilitated the prediction of 48 families and 1439 miRNAs, of which 798 miRNAs have been reported previously and 349 are novel miRNAs. Thirteen miRNAs that were closely related to the N metabolic pathway were screened in this study, and degradome analysis showed that most of these miRNAs showed a many-to-many relationship with target genes. The results of GO and KEGG enrichment analyses revealed that numerous biological processes and metabolic pathways were involved in N metabolism, carbon metabolism, and phytohormone biosynthesis. We screened the miRNAs associated with N metabolism pathways and related pathways. The validation of the screened differential miRNAs by qRT-PCR showed that *stu-miR396-5p*, *stu-miR408b-3p_R-1*, *stu-miR3627-3p*, *stu-miR482a-3p*, *stu-miR8036-3p*, *stu-miR482a-5p*, *stu-miR827-5p*, *stu-miR156a_L-1*, *stu-miR827-3p*, *stu-miR172b-5p*, *stu-miR6022-p3_7*, *stu-miR398a-5p*, and *stu-miR166c-5p_L-3* were all found in the Yanshu4 and Atlantic varieties, at the seedling and budding stages, and differential expression was observed in the root and leaf in response to N stress. The most significant differences in miRNAs were observed for *stu-miR396-5p*, *stu-miR8036-3p*, and *stu-miR482a-3p*. These miRNAs, especially *stu-miR396-5p*, were down-regulated in the seedling stage and roots, and in the budding-stage leaves and roots of the Atlantic variety under both types of N stress; *stu-miR482a-3p* was down-regulated in the seedling stage and roots and up-regulated in the budding-stage leaves and roots; *stu-miR482a-3p* was up-regulated in the seedling stage and roots, and the budding-stage leaves. The expression of *stu-miR482a-3p* was down-regulated in the seedling stage and roots, and up-regulated in leaves and roots at the budding stage; *stu-miR8036-3p* was up-regulated in the seedling stage and roots, and leaves and roots at the budding stage. Previously, it was found that the activities of NiR in the leaves and roots of the Yanshu4 and Atlantic varieties were positively correlated at the seedling and budding stages in varieties that were not treated or excessively treated with N, and both were highly responsive to N stress. qRT-PCR results revealed that *NiR* was positively correlated with the expression levels in the seedlings, buds, roots, and leaves of both the Yanyan4 and Atlantic varieties, and *stu-miR396-5p* was negatively correlated with the expression levels in the seedlings, buds, roots, and leaves of both the Yanyan4 and Atlantic varieties that were either not treated or excessively treated with N, and the difference in the expression level was highly significant. The splicing relationship between *StNiR* and *stu-miR396-5p* was identified using online prediction software, and this relationship was verified using the luciferase assay.

## Materials and methods

### Plant material and treatments

The tetraploid N-efficient potato variety Yanshu4 (*Solanum tuberosum* L. var. Yanshu4) (Y) and N-inefficient Atlantic (*S. tuberosum* L. var. Atlantic) (D) variety, used as test materials, were provided by the Potato Innovation Team at Jilin Agricultural University. The pot test was performed, and culture growth was assessed under open field conditions. Seed potatoes were cut, treated for potting, and subjected to two types of N stress after potting, i.e., a complete lack of application of N fertilizer at N: 0 kg/667 m^2^ and over-application of N fertilizer at N: 25 kg/667 m^2^; the main source of N fertilizer was urea (containing 46% N). Calcium superphosphate (containing P_2_O_5_ 46%) was applied uniformly at a rate of 18 kg/667 m^2^, and potassium sulfate (containing K_2_O 50%) was applied at a rate of 36 kg/667 m^2^. All fertilizers were applied once as base fertilizer, and 10 pots were planted during each the treatment process; three biological replicates were set up. Roots and leaves were collected at the seedling stage (15 days after seedling emergence, S) and budding stage (30 days after seedling emergence, A), snap-frozen in liquid nitrogen, stored at -80℃, and sent to Biotechnology Ltd. for miRNA sequencing, degradome analysis, and qRT-PCR validation.

### Construction of miRNA and degradation libraries

The miRNA library was constructed in accordance with the standard procedure provided by Illumina for library preparation and sequencing-related experiments. miRNAs from the sequencing library TruSeq were prepared using Small RNA Sample Prep Kits (Illumina, San Diego, USA).

End-repair technology was used to construct the degradation group library and perform 5'-adaptor ligation, 3'-adaptor ligation, reverse transcription, short PCR amplification, enzyme digestion, 3' double chain DNA joint ligation, and long PCR amplification [53]. Library construction was carried out on the basis of Axtel1 [54] degradome library construction, which was further optimized and simplified by the introduction of the bead screening process. After library preparation, the constructed libraries were sequenced using the Illumina HiSeq2000/2500 (Illumina, San Diego, USA)instrument at a single-end read length of 1 × 50 bp. See Additional file 1, Table 5 for all database URLs used.

### Analysis of miRNAs and prediction of target genes

Through a series of data processing steps, the original data obtained during sequencing can be used to generate comparable pairs of sequences for subsequent analysis. The re-alignment of sequences with sequences of species obtained via cDNA database sequence alignment generated a degradation group density file (degradome density file). Then, using shear site prediction software (GSTAr), we predicted and sequenced species miRNA sequence pairs of target gene sequences. Finally, the predicted miRNAs and their corresponding target genes were combined with the target genes in the generated degradome data, to identify common target genes.

The degradation group was analyzed using the Cleave Land Program [55]. The application OliGO map for the short reading frame calibrator enabled us to identify the target genes matching the degradation group sequence [56]. The OliGO map was again applied to extract 13 sequences upstream and 13 sequences downstream of the paired site for each accurately paired degradome sequence, to form a 26-nt target gene, and the Needle program from the EMBOSS package was applied to derive all the sequences that matched the sequences in the provided miRNA library. Then, columns were scored against the plant miRNAs/target pairing criteria. The score should not exceed a user-set threshold and should retain 10 nt at the 5' end of the degradome sequence paired with the miRNA.

### miRNA identification and prediction of expression profiles

The expression profile information of miRNAs was identified using a normalization method. First, a regular sequence was identified in all samples, a reference data family was constructed, and a logarithmic transformation with base 2 was performed using the copy number for all samples and the reference data family (log2(copy#)). Then, the differences in the Δlog2(copy#) between the respective samples and the reference data family were determined, |Δlog2(copy#)| was set at < 2 sequences, and finally, the correction factor algorithm fi = 2Δyi was applied for sample i; the number of copies in each sample was corrected by multiplying the original number of copies by the algorithm correction factor fi.

### Identification and functional annotation of target genes

Predicted target genes were mapped to the potato genome GDDH13 Version 1.1 (https://iris.angers.inra.fr/GDDH13/the-apple-genome-downloads.html) using HISAT2 (Johns Hopkins University Center for Computational Biology, Baltimore, MD, USA). Differently expressed genes with |log2(fold change)|≥ 1 and statistically significant values (*P* < 0.05) were selected using the R package Ballgown.

Enrichment analysis requires functional annotation to be performed using the GO database (http://www.geneontology.org/) and the KEGG pathway database [57] (http://www.genome.jp/KEGG/) [58]. First, the number of genes was counted per function or pathway for all target gene annotations corresponding to the selected miRNAs. Subsequently, a hypergeometric test was applied to determine the number of genes corresponding to the GO or KEGG pathway [59] in the annotation library (for all functionally annotated genes or all functionally annotated genes with a *p*-value ≤ 0.05, the threshold value was calculated (the default threshold is Score ≤ 2.5), and functions satisfying this condition were defined as those that were significantly enriched in miRNA-mRNA pairs. Functional enrichment analysis was used to identify the main biological functions of miRNA-mRNA relationships.

### Quantitative real-time reverse transcription polymerase chain reaction analysis

Upstream primers for miRNAs were designed using miRNA Design V1.01 software (see Table 6 in Additional file 1 for details) with the universal reverse transcription primer: 3'- CAGCATAGGTCACGTCCCAGGCTCCAT AAGCGTGACCTATGCTGTTCAAG—5' and the universal downstream primer: 5'- AGT GCAGGGTCCGAGGTATT—3'. The cDNA synthesis of the first strand of stem-loop miRNAs was performed using the MR101 kit (Vazyme Biotech Co., Ltd). The reaction system included 2 µl of 5 × gDNA Wiper Mix and RNase-free ddH_2_O up to a volume of 10 µl; mixing was performed using a pipette. The reaction was allowed to occur at 42 °C for 2 min to remove genomic DNA. Subsequently, 1 µl of Stem-loop Primer (2 µM), 2 µl of 10 × RT Mix, and 2 µl of HiScriptII Enzyme Mix were added, and finally, RNase-free ddH_2_O was added to make up the volume to 20 µl. After mixing, the reaction conditions were as follows: 25 °C for 5 min, 50 °C for 15 min, and 85 °C for 5 min. The synthesis of the first-strand cDNA was completed. The internal reference gene was the potato *elf1-α* gene (Gene ID: 118,059,944) with the forward primer sequence: 5'- CAAGGATGACCCAGCCAAG -3' and the reverse primer sequence: 5'- TTCCTTACCTGAACGCCTGT -3' [60], and qRT-PCR was performed using an MQ101-01 kit obtained from VAZYME (Nanjing). The expression levels of miRNAs were detected by stem-loop RT-PCR using miRNA- specific stem-loop primers. The following mix was prepared in a qPCR tube: 10.0 µl of 2 × miRNAs from Universal SYBR qPCR Master Mix, 0.4 µl of each Specific Primer (10 µM), 0.4 µl of mQ Primer R (10 µM), 1 µl of template DNA/cDNA, and RNase-free ddH_2_O to make up the volume to 20.0 µl. The qRT-PCR reaction was performed in three biological replicates under the following reaction conditions: 5 min at 95 °C, 40 cycles of 10 s at 95 °C and 30 s at 60 °C. Melting curve analysis was performed to determine product specificity. Finally, the fold change in miRNA expression was calculated using the 2^−ΔΔCt^ method.

The miRNA data were uploaded to the GEO database (GEO: GSE199457).

### Luciferase binding site detection

We used histoculture seedlings of the N-efficient Yanshu4 variety and the N-inefficient Atlantic potato variety, extracted total RNA, obtained the first clone of the *StNiR* sequence via PCR amplification (see Table 7 in Additional file 1, Additional file 3, and Additional file 4 for details), and predicted the site at which binding to *stu-miR396-5p* occurred via the Miranda online website.

The pCAMBIA1300-LUC-NiR and pCAMBIA1300-35S-396-5p expression vectors were constructed (see Additional file 3 for details) and transformed into *Agrobacterium* strains. Then, 2–4-week-old tobacco plants were selected for luciferase assay using the Dual-Luciferase Assay System (Promega Inc.). The luciferase activity was analyzed on a Promega luminescence detector. A total of four test groups were set up, namely test group 1: NiR-luc bound to miR396-5p, test group 2: NiR-luc bound to 1300-35S-X, test group 3:1300-luc bound to NiR-luc, and test group 4: 1300-luc bound to 1300-35S-X, where 1300-35S-X was the control for miR396-5p, and 1300-luc was the control for NiR-luc. Finally, the fluorescence value measured by the dual reporter system was used to calculate the fluorescence value of the target plasmid/control plasmid (i.e., the F/R value), and the ratio relative to the control was calculated. Significance analysis was performed and bar graphs were generated.

## Supplementary Information

**Additional file 1: Table 1.** Summarystatistics of the miRNA sequencing data of two potato varieties at the seedlingand budding stages supplied with different levels of  N. **Table 2.** Prediction of highly expressed miRNAs. **Table 3.** Prediction of target genes for differentialmiRNAs. **Table 4.** Enrichment analysis of target genescorresponding to differential miRNAs. **Table 5.** URLs of the online platforms. **Table 6.** Details of qRT-PCR primers for miRNAs. **Table 7.** Primer sequences. 

**Additional file 2: Figure 1.** Analysis of variability among groups of differential miRNAsHN: excessive N application; LN: no N application; Y: Yanshu4; D: Atlantic;S: seedling stage; A: budding stage (A) LN_YLSvsLN_DLS (B) LN_YLAvsLN_DLA (C) HN_YLSvsHN_DLS. (D)HN_YLAvsHN_DLA (E) HN_DLSvsLN_DLS (F) HN_DLAvsLN_DLA. (G) HN_YLSvsLN_YLS (H)HN_YLAvsLN_YLA (I) LN_DLAvsLN_DLS. (J)HN_DLAvsHN_DLS (K)LN_YLAvsLN_YLS (L)HN_YLAvsHN_YLS.(M)LN_YRSvsLN_DRS (N)LN_YRAvsLN_DRA (O)HN_YRSvsHN_DRS. (P)HN_YRAvsHN_DRA (Q)HN_DRSvsLN_DRS(R)HN_DRAvsLN_DRA. (S)HN_YRSvsLN_YRS (T)HN_YRAvsLN_YRA (U)LN_DRAvsLN_DRS. (V)HN_DRAvsHN_DRS(W)LN_YRAvsLN_YRS (X)HN_YRAvsHN_YRS. Using the values of log_2_(fold change) as the horizontalcoordinate and -log_10_(*p*-value) as the vertical coordinate, volcanoplots were constructed for all miRNAs during differential expression analysis.The horizontal coordinate represents the fold change in the differentialexpression of miRNAs in different samples, and the vertical coordinaterepresents the statistical significance of the difference in the change inexpression levels of miRNAs. The red color represents up-regulatedsignificantly differentially expressed genes, the blue color representsdown-regulated significantly differentially expressed genes, and the grey dots representnon-significant differentially expressed genes. **Figure 2.** Comparison between groups of differential miRNAs. HN: excess N; LN:no N; Y: Yanshu4; D: Atlantic; S: seedling stage; A: budding stage. Thehorizontal coordinates indicate the data obtained after the comparison ofgroups, and the vertical coordinates indicate the number of up- anddown-regulated miRNAs. The red color represents up-regulated miRNAs, the bluecolor represents down-regulated miRNAs, and the numbers represent the number ofup- and down-regulated miRNAs. **Figure 3.** Results of clustering analysis ofdifferential miRNAs. HN: excess N; LN: no N; Y: Yanshu4; D: Atlantic; S: seedling;A: bud onset. (A)LN_YLSvsLN_DLS (B)LN_YLAvsLN_DLA (C)HN_YLSvsHN_DLS. (D)HN_YLAvsHN_DL.(E)HN_DLSvsLN_DLS (F)HN_DLAvsLN_DLA. (G)HN_YLSvsLN_YLS(H)HN_YLAvsLN_YLA (I)LN_DLAvsLN_DLS.(J)HN_DLAvsHN_DLS (K)LN_YLAvsLN_YLS (L)HN_YLAvsHN_YLS. (M)LN_YRSvsLN_DRS (N)LN_YRAvsLN_DRA(O)HN_YRSvsHN_DRS. (P)HN_YRAvsHN_DRA (Q)HN_DRSvsLN_DRS (R)HN_DRAvsLN_DRA. (S)HN_YRSvsLN_YR.(T)HN_YRAvsLN_YRA (U)LN_DRAvsLN_DRS. (V)HN_DRAvsHN_DRS (W)LN_YRAvsLN_YRS (X)HN_YRAvsHN_YRS

**Additional file 3.**

**Additional file 4: Figure 1.** Construction of therecombinant plasmid pCAMBIA1300-LUC-NiR for the luciferase reporter vector. **Figure 2.** Construction of the recombinant plasmid pCAMBIA1300-35S-396-5p for theluciferase localization report vector.

## Data Availability

The data content has been submitted to GEO database on 2022.3.25, and will be publicly released on 2024.2.28. The following links allow you to view the uploaded data: To review GEO accession GSE199457: Go to https://www.ncbi.nlm.nih.gov/geo/query/acc.cgi?acc=GSE199457. Enter token unedcscqzpahxet into the box.
