# Supplementary material for: Screening of differentially expressed microRNAs and target genes in two potato varieties under nitrogen stress
Source: BMC Plant Biol. 2022 Oct 8;22:478. doi: 10.1186/s12870-022-03866-5 (PMC9547441; doi:10.1186/s12870-022-03866-5)
Supplement: Supplementary file 1 — Additional file 1: Table 1. Summarystatistics of the miRNA sequencing data of two potato varieties at the seedlingand budding stages supplied with different levels of N. Table 2. Prediction of highly expressed miRNAs. Table 3. Prediction of target genes for differentialmiRNAs. Table 4. Enrichment analysis of target genescorresponding to differential miRNAs. Table 5. URLs of the online platforms. Table 6. Details of qRT-PCR primers for miRNAs. Table 7. Primer sequences. [file 12870_2022_3866_MOESM1_ESM.docx]

Appendix A

Table 1 Summary statistics of the miRNA sequencing data of two potato varieties at the seedling and budding stages supplied with different levels of N

| Sample | #Raw reads | #Clean reads | #valid reads | Sample | #Raw reads | #Clean reads | #valid reads |
| --- | --- | --- | --- | --- | --- | --- | --- |
| HN_YRS | 3588321 | 798832 | 2715479 | HN_DLA | 2149180 | 496144 | 1594006 |
| LN_DRS | 3722148 | 461691 | 3167781 | LN_DLA | 2262475 | 393626 | 1800986 |
| LN_YRS | 4365972 | 654661 | 3608159 | LN_YLA | 3557690 | 432794 | 3036495 |
| HN_DRS | 3400126 | 781384 | 2539099 | HN_YLA | 3610163 | 536264 | 2972948 |
| LN_DLS | 2028663 | 491863 | 1486354 | LN_YRS | 3977173 | 615711 | 3264162 |
| HN_DLS | 2484537 | 364370 | 2057157 | LN_YLS | 1929647 | 419409 | 1445533 |
| LN_YLS | 2536227 | 593609 | 1863842 | HN_DRA | 3695790 | 906374 | 2705514 |
| HN_YLS | 2748679 | 461829 | 2197444 | HN_YRA | 3159747 | 831793 | 2250381 |
| HN_DRA | 3633747 | 592260 | 2954610 | HN_DRA | 3016140 | 974343 | 1979869 |
| LN_DRA | 3982557 | 871148 | 3030489 | LN_YLA | 2580474 | 588205 | 1914532 |
| LN_YRA | 3616725 | 888301 | 2648983 | HN_YLA | 3070297 | 545268 | 2433888 |
| HN_YRA | 3872736 | 799781 | 2982378 |  |  |  |  |

HN: over-application of N; LN: no application of N; Y: Yanshu4; D: Atlantic; S: seedling stage; A: budding stage

Raw reads indicate raw sequencing data, clean reads indicate preliminary filtered data, and valid reads indicate valid filtered data, as compared to the database.

Table 2 Prediction of highly expressed miRNAs

| miR_name | miR_sequence | miR_name | miR_sequence |
| --- | --- | --- | --- |
| *stu-miR156a* | TTGACAGAAGATAGAGAGCAC | *stu-miR408b-5p* | ACGGGGACGAGACAGAGCATG |
| *stu-miR156a* | TTGACAGAAGATAGAGAGCAC | *stu-miR408b-3p_R-1* | TGCACTGCCTCTTCCCTGGC |
| *stu-miR156e* | TGACAGAAGAGAGTGAGCAC | *stu-miR477b-5p* | ACTCTCCCTCAAAGGCTTCTG |
| *stu-miR160a-3p* | GCGTATGAGGAGCCAAGCATA | *stu-miR477a-5p* | CCTCTCCCTCAAGGGCTTCTC |
| *stu-miR162a-5p* | GGAGGCAGCGGTTCATCGATC | *stu-miR479* | TGAGCCGAACCAATATCACTC |
| *stu-miR162a-3p* | TCGATAAACCTCTGCATCCAG | *stu-miR482d-5p* | CGTGAGTGGTGGGGTAAGATA |
| *stu-miR164-5p_2ss17AG21TA* | TGGAGAAGCAGGGCACGTGCA | *stu-miR482d-3p* | TCTTGCCTACACCGCCCATGCC |
| *stu-miR166a-3p* | TCGGACCAGGCTTCATTCCCC | *stu-miR482b-5p* | GGAGTGGGTGGCATGGTAAGA |
| *stu-miR166a-5p_1ss21GA* | GGAATGTTGTCTGGCTCGAGA | *stu-miR482b-3p* | TTACCGATTCCCCCCATTCCAA |
| *stu-miR167a-5p* | TGAAGCTGCCAGCATGATCTA | *stu-miR482a-5p* | GGAATTGGTGGATTGGAAAGC |
| *stu-miR167d-3p* | GATCATGTGGTTGCTTCACC | *stu-miR482a-3p* | TTTCCAATTCCACCCATTCCTA |
| *stu-miR171b-3p* | TTGAGCCGCGTCAATATCTCT | *stu-miR482e-5p* | AGTGGGTGGTGTGGTAAGATT |
| *stu-miR171d-5p_L-2R+3* | ATATTGGTGCGGTTCAATTAGA | *stu-miR482e-3p* | TCTTGCCAATACCGCCCATTCC |
| *stu-miR171a-3p* | TGATTGAGCCGTGCCAATATC | *stu-miR482c* | TTTCCTATTCCACCCATGCCAA |
| *stu-miR171a-5p* | TATTGGCCTGGTTCACTCAGA | *stu-miR827-3p* | TTAGATGAACATCAACAAACA |
| *stu-miR171c-3p* | TGATTGAGCCGTGTCAATATC | *stu-miR827-5p* | TTTGTTGATGGTCATCTATTC |
| *stu-miR171a-3p* | TGATTGAGCCGTGCCAATATC | *stu-miR1919-5p* | TGTCGCAGATGACTTTCGCCC |
| *stu-miR172b-5p* | GCAGCACCATCAAGATTCACA | *stu-miR1919-3p* | ACGAGAGTCATCTGTGACAGG |
| *stu-miR172a-3p* | AGAATCTTGATGATGCTGCAT | *stu-miR6022* | TGGAAGGGAGAATATCCAGGA |

Table 2 (continued)

| *stu-miR319a-5p_R-1* | AGAGCTTTCTTCGGTCCACA | *stu-miR6022* | TGGAAGGGAGAATATCCAGGA |
| --- | --- | --- | --- |
| *stu-miR319a-3p* | TTGGACTGAAGGGAGCTCCCT | *stu-MIR6023-p3* | CCAAAGACAAGTCATGGAATA |
| *stu-miR319b_R+1* | TTGGACTGAAGGGAGCTCCTT | *stu-miR6024-5p* | AGAAACAACACTTGCTAAAAGA |
| *stu-miR384-5p* | TTGGCATTCTGTCCACCTCC | *stu-miR6024-3p* | TTTTAGCAAGAGTTGTTTTCCC |
| *stu-miR390-5p* | AAGCTCAGGAGGGATAGCACC | *stu-miR6025* | TACCAACAATTGAGATAACATC |
| *stu-miR391-5p_L-1R+2* | ACGCAGGAGAGATGATGCTGGA | *stu-MIR6025-p3* | TGTTATCTCAGTGTTGGCATG |
| *stu-miR391-3p_L+2R-1* | CAGCATCATACTCCTGCATAT | *stu-miR6027_R-1* | TGAATCCTTCGGCTATCCATA |
| *stu-miR396-5p* | TTCCACAGCTTTCTTGAACTT | *stu-miR6149-5p* | TTGCAACACACCTGAATCGTC |
| *stu-miR396-3p* | GTCCAAGAAAGCTGTGGGAAA | *stu-miR8036-5p* | GGAGGAATCGAAAGATATAAG |
| *stu-miR398b-3p* | TTGTGTTCTCAGGTCACCCCT | *stu-miR8036-3p* | TATGTCTTTCCGATGCCTCCCA |
| *stu-miR398a-5p* | GGGTTGATTTGAGAACATATG | *PC-5p-181_67265* | CGGGTGCTTACTCAACTAATA |
| *stu-miR398a-3p* | TATGTTCTCAGGTCGCCCCTG | *PC-3p-20_524513* | TTGGTTGAGTGAGCATCTAAG |
| *stu-miR408a-3p* | TGCACAGCCTCTTCCCTGGTT |  |  |

Naming of miRNAs: L-n indicates that n bases are missing at the leftmost end of the reported miRNA; R-n indicates that n bases are missing at the rightmost end of the reported miRNA; L+n indicates that n bases more are present at the left end of the reported miRNA; R+n indicates that n bases more are present at the right end of the reported miRNA; 2ss5TC13TA indicates that n bases are missing at the 5^th^ base, at which T is substituted by C (ss, substitution), and at the 13^th^ base, at which T is substituted by A; thus, a total of 2 substitutions occur. The absence of the above annotation indicates a full match with rep_miRNA, rep_mir/MIR. If only mir/MIR is matched, but not miR, then mir/MIR-"p3" and "p5" are used to indicate the position at the arm end of mir/MIR. This is immediately distinguished from miR-3p/5p. Novel miRNAs are labelled as PCs ('Predicted Candidates'), and this label indicates that they exhibit the 5p or 3p end-of-arm position.

Table 3 Prediction of target genes for differential miRNAs

| miRNA | Target gene | miRNA | Target gene |
| --- | --- | --- | --- |
| *PC-3p-312355_41* | *GLN2* | *stu-miR156g-3p* | *NIR* |
| *PC-3p-330354_37* | *PCMP-E105* | *stu-miR319-3p* | *NIR* |
| *PC-3p-62135_418* | *GLU1* | *stu-miR3627-3p* | *HSP70-14* |
| *PC-3p-92045_261* | *GLU1* | *stu-miR396-5p* | *NIR* |
| *PC-5p-147185_139* | *GLU1* | *stu-miR396-5p_L-3* | *NIR1* |
| *stu-MIR319-p5* | *PCMP-H66* | *stu-miR396-5p_L-3* | *-* |
| *stu-MIR397-p3_1ss13TC* | *GLN2* | *stu-miR408b-3p_R-1* | *PCMP-H85* |
| *stu-MIR5303j-p5_2ss10GT17CT* | *NRT2.7* | *stu-miR8036-3p* | *NIT4B* |
| *stu-MIR5303j-p5_2ss10GT17CT* | *NIR* | *stu-miR482a-5p* | *ASP3* |
| *stu-MIR5303j-p5_2ss10GT17CT* | *NIR1* | *stu-miR156a_L-1* | *NRT2.5* |
| *stu-MIR6022-p3_7* | *GLU1* | *stu-miR827-3p* | *SD31* |
| *stu-MIR7981-p5_2ss15AG17AG* | *-* | *stu-miR172b-5p* | *grpE* |
| *stu-MIR8006-p3_1ss9AG* | *NIR1* | *stu-miR482a-3p* | *R1B-16* |
| *stu-MIR8020-p3* | *GLN2* | *stu-miR827-5p* | *CRY2* |
| *stu-MIR8044-p5_1ss13GC_1* | *GLU1* | *stu-miR398a-5p* | *PSBY* |
| *stu-MIR8047-p3_1ss20TC* | *-* | *stu-miR166c-5p_L-3* | *CAT1* |
| *stu-miR156d-3p_R-1* | *NIR* |  |  |

Table 4 Enrichment analysis of target genes corresponding to differential miRNAs

| miRNA | Target gene | miRNA | Target gene |
| --- | --- | --- | --- |

Table 4 (continued)

| *stu-miR156a_L-1* | *SRF6*; *TPIP1*; *TGA1B*; *At1g1032*; *ynbB*; *SPL6*; *RR10*; *spb1*; *SBP1*; *LKHA*; *AGL15*; *ASP5*; *LECRK91*; *At1g43190*; *NRT2.5*; *HLP*; *SPL12*; *SPL15*; *SPL10*; *SBP1*; *CAT1*; *At4g21705*; *CAT1*; *SPL3*; *TGA1*; *PLIM2A*; *At5g38390*; *eIF-2gamma* | *stu-MIR6022-p3_7* | *CYTB5-B*; *MDIS2*; *GLU1*; *RLP12*; *MIK1*; *RLP30*; *RLP12*; *RPP13*; |
| --- | --- | --- | --- |
| *stu-miR827-3p* | *At4g22990*; *EXD1*; *At4g22990*; *LCKB2*; *SD31*; *RS31*; *AAP3*; *ANTR5*; *ML5*; *CYP71D55*; *HERC2*; *PIP5K6*; *IJ*; *R1B-17* | *stu-miR398a-5p* | *PSBY*; *STY17*; *ATX1*; *Os12g0234800* |
| *stu-miR172b-5p* | *WRKY48*; *At4g29360*; *At1g11820*; *grpE*; *NAC100*; *CPN60-2*; *IRX12*; *MT2287*; *ROC5* | *stu-miR166c-5p_L-3* | *CAT1*; *PEX11B*; *GT7*; *At3g47110*; *LECRKS5*; *At1g11300*; *FAM63B*; *CMB1*; *HMGCL; IRE4*; *At4g34215* |
| *stu-miR408b-3p_R-1* | *UCC1*; *LAC5*; *TPS32*; *TPS31*; *PCMP-H85*; *DTX21*; *LAC5*; *BRG3*; *KCS4* |  |  |

Table 5 URLs of the online platforms

| Database | Web Link | Version Date |
| --- | --- | --- |
| miR (miRs) database | <ftp://mirbase.org/pub/mirbase/CURRENT/> | 22.0 |
| Pre-miRNA (mirs/MIRs) database | <ftp://mirbase.org/pub/mirbase/CURRENT/> | 22.0 |
| Species Priority | Stu |  |
| Rfam | Collection of many common non-coding RNA families except microRNA | 12.0 |
|  | <http://rfam.janelia.org> |  |
| Repbase | Prototypic sequences representing repetitive DNA from diﬀerent eukaryotic species | 22.07 |
|  | <http://www.girinst.org/repbase> |  |
| Genome Database | <https://www.sgn.cornell.edu/organism/Solanum_tuberosum/genome> |  |
| mRNA Database | <https://www.sgn.cornell.edu/organism/Solanum_tuberosum/genome> |  |
| KEGG pathway database | <http://www.genome.jp/kegg> | 2016.05 |
| Gene Ontology Database | <ftp://ftp.ncbi.nih.gov/gene/DATA/gene2go.gz> | 2016.04 |
| miRanda | http://www.bioinformatics.com.cn/local_miranda_miRNA_target_prediction_120 |  |

Table 6 Details of qRT-PCR primers for miRNAs

| Name | Sequence | Symbol | Primer(5´-3´) |
| --- | --- | --- | --- |
| *stu-miR396-5p* | TTCCACAGCTTTCTTGAACTT | *NiR*(Nitrite reductase) | CGCGTTCCACAGCTTTCTT |
| *stu-miR8036-3p* | TATGUCTTTCCGATGCCTCCCA | *NIT4B*(Bifunctional nitrilase/nitrile hydratase NIT4B) | CGCGTATGTCTTTCCGATGC |
| *stu-miR482a-5p* | GGAATTGGTGGATTGGAAAGC | *ASP3(aspartate aminotransferase 3 )* | GCGGGAATTGGTGGATTG |
| *stu-miR156a_L-1* | TTGACAGAAGATAGAGAGCAC | *NRT2.5*(nitrate transporter 2.5 ) | CGCGCGTTGACAGAAGATAGA |

Table 6 (continued)

| *stu-miR827-3p* | TTAGATGAACATCAACAAACA | *SD31(G-type lectin S-receptor-like serine/threonine-protein kinase SD3-1 )* | CGCGCGTTAGATGAACATCAA |
| --- | --- | --- | --- |
| *stu-miR172b-5p* | GCAGCACCATCAAGATTCACA | *grpE(nucleotide exchange factor GrpE )* | GCGGCAGCACCATCAAGA |
| *stu-miR408b-3p_R-1* | TGCACTGCCTCTTCCCTGGC | *PCMP-H85(putative pentatricopeptide repeat-containing protein At3g13770)* | CGCGCGCGGCACGCCC |
| *stu-miR3627-3p* | AAGTGCCTCTGTCTTTCGACA | *HSP70-14(Heat shock protein 70 (Hsp 70) family protein)* | CGCGAAGTGCCTCTGTCTT |
| *stu-miR482a-3p* | TTTCCAATTCCACCCATTCCTA | *FZR1(putative pentatricopeptide repeat-containing protein At3g13770)* | GCGTTTCCAATTCCACCCA |
| *stu-miR827-5p* | TTTGTTGATGGTCATCTATTC | *GAUT15(galacturonosyltransferase 15)* | GCGCGTTTGTTGATGGTCAT |
| *stu-miR6022-p3_7* | TGGTATTGTTCCGTTCAGG | *CYTB5-B(cytochrome B5)* | GCGCGTGGAAGGGAGAATAT |
| *stu-miR398a-5p* | GGGTTGATTTGAGAACATATG | *PSBY(photosystem II BY)* | CGCGGGGTTGATTTGAGAA |
| *stu-miR166c-5p_L-3* | ATGTTGTTTGGCTCGAGG | *CAT1(catalase 1)* | GCGGGAATGTTGTTTGGC |
| *ef1-α*--F | —— | —— | CAAGGATGACCCAGCCAAG |
| *ef1-α*--R | —— | —— | TTCCTTACCTGAACGCCTGT |

Table 7 Primer sequences

| Primer name | Primer sequence (5´-3´) |
| --- | --- |
| *StNiR* F | ATGACATCTTTTTCGGTTAAATT |
| *StNiR* R | TTACTCTTCTGTTTCTTCTCTTTCT |
| pCAMBIA1300-LUC-StNiR F | CGGCAAGATCGCCGTGTAACTGCAGATGACATCTTTTTCGGTTAAATT |
| pCAMBIA1300-LUC-StNiR R | gatgatacgaacgaaagctCTGCAGTTACTCTTCTGTTTCTTCTCT |
| pCAMBIA1300-35S-P396-5p | GGATCCTTCCACAGCTTTCTTGAACGTACTAGT |
| pCAMBIA1300-35S-PP396-5p | GGTACCACGTTCAAGAAAGCTGTGGAAGAGCTC |
