# Supplementary material for: Screening of differentially expressed microRNAs and target genes in two potato varieties under nitrogen stress
Source: BMC Plant Biol. 2022 Oct 8;22:478. doi: 10.1186/s12870-022-03866-5 (PMC9547441; doi:10.1186/s12870-022-03866-5)

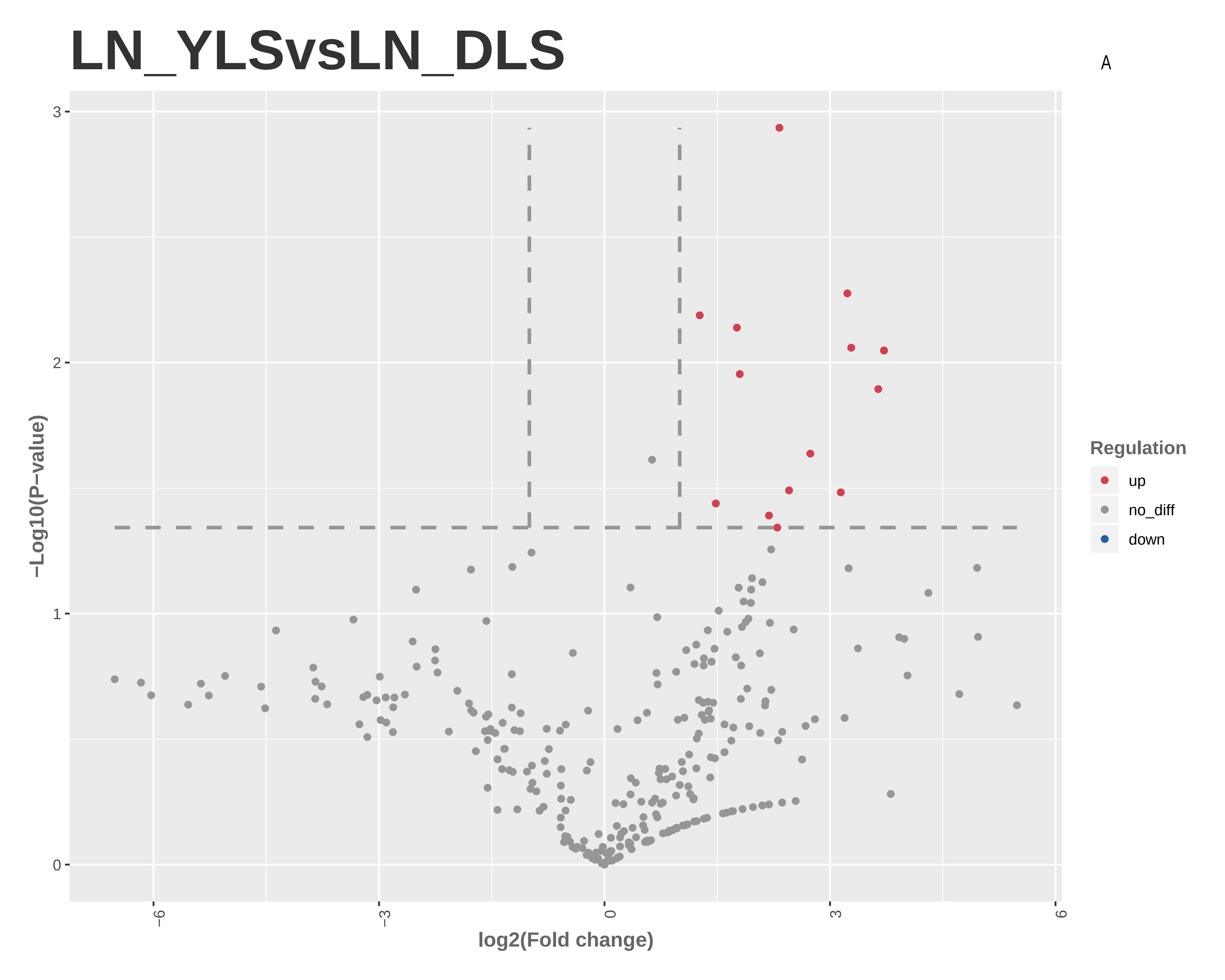

LN_YLSvsLN_DLS
A
−Log10(P−value)
3
2
1
0
−6
−3
0
3
6
log2(Fold change)
Regulation
up
no_diff
down

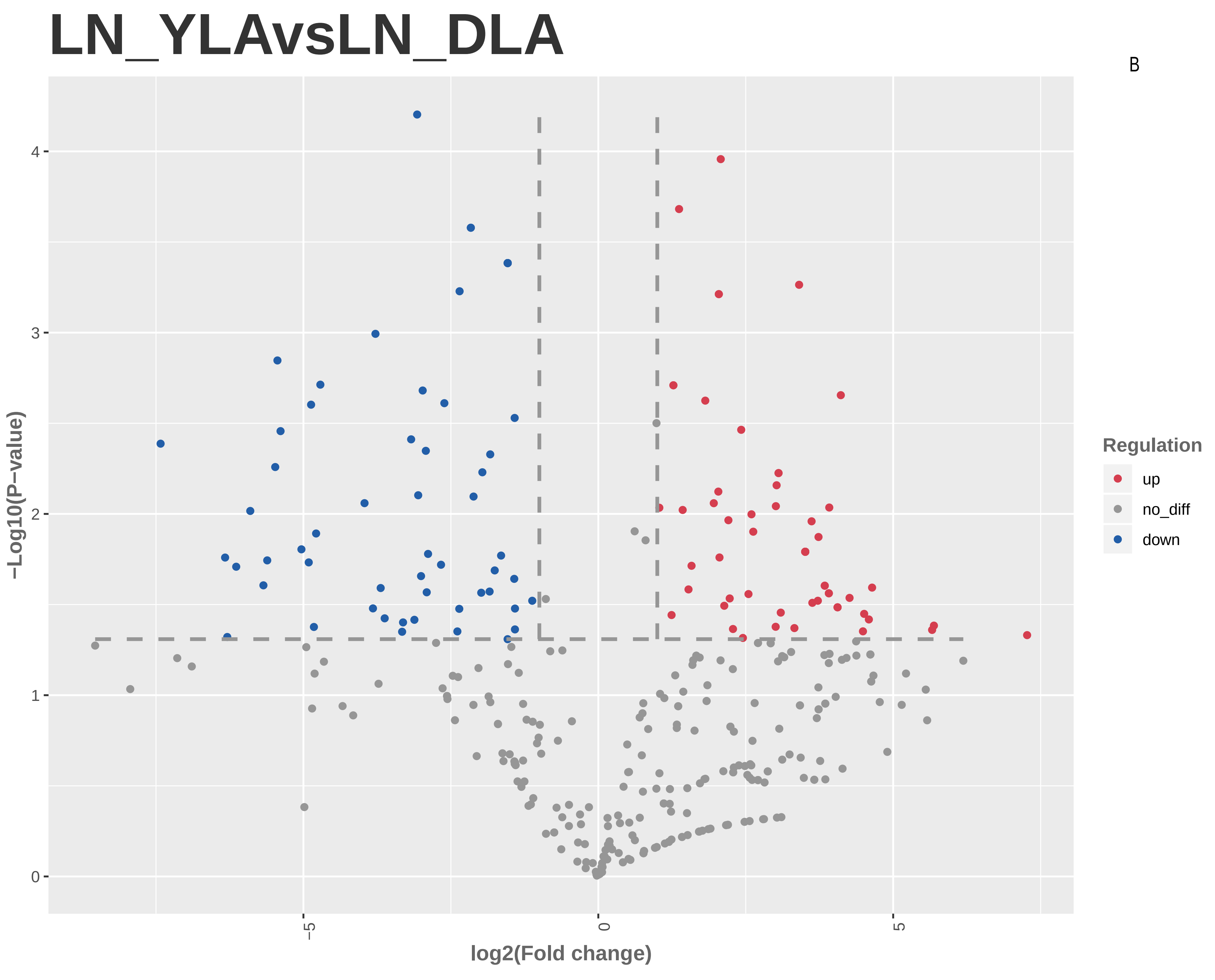

LN_YLAvsLN_DLA
B
−Log10(P−value)
log2(Fold change)
Regulation
up
no_diff
down
0
1
2
3
4
−5
0
5

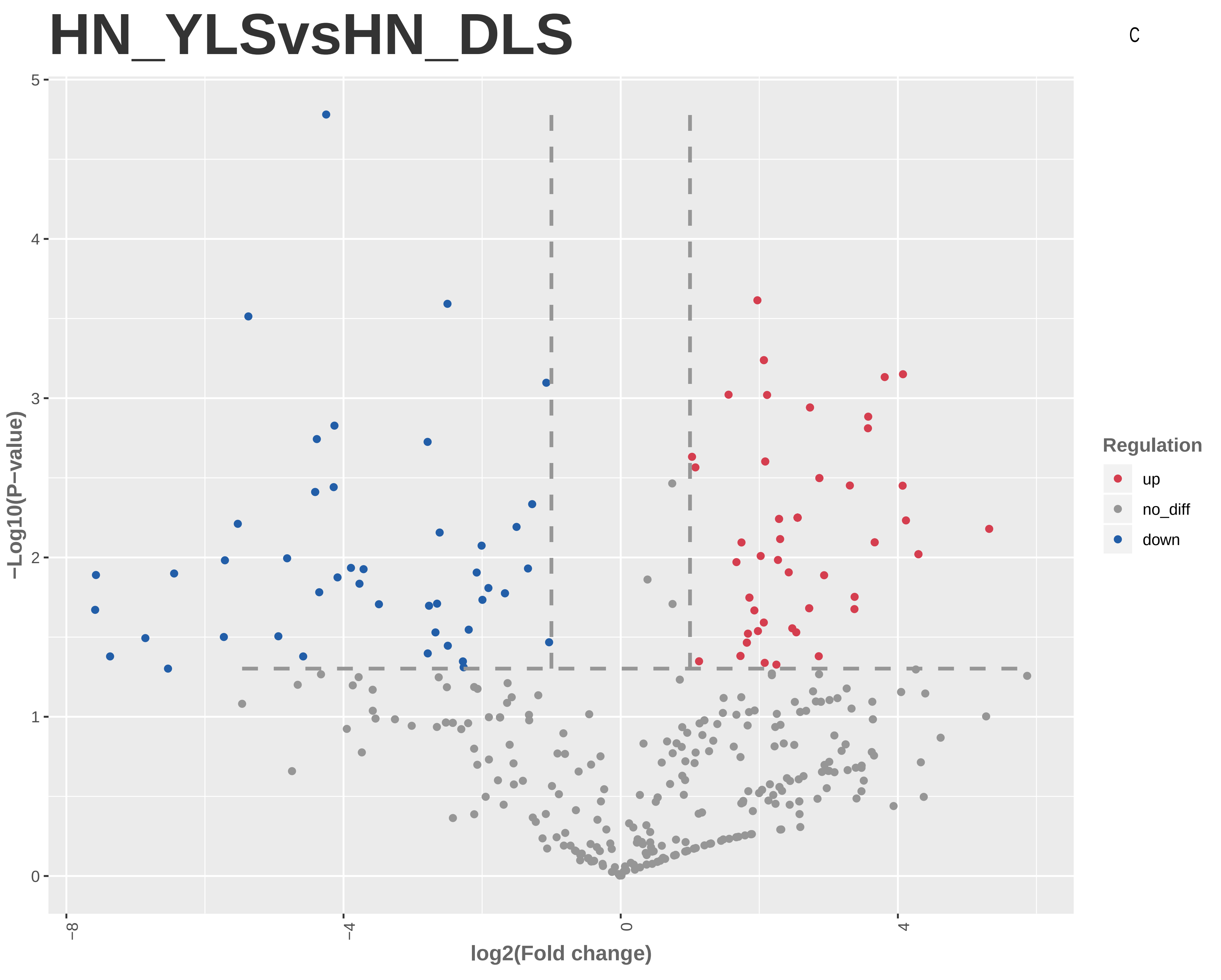

HN_YLSvsHN_DLS
C
−Log10(P−value)
0
1
2
3
4
5
−8
−4
0
4
log2(Fold change)
Regulation
up
no_diff
down

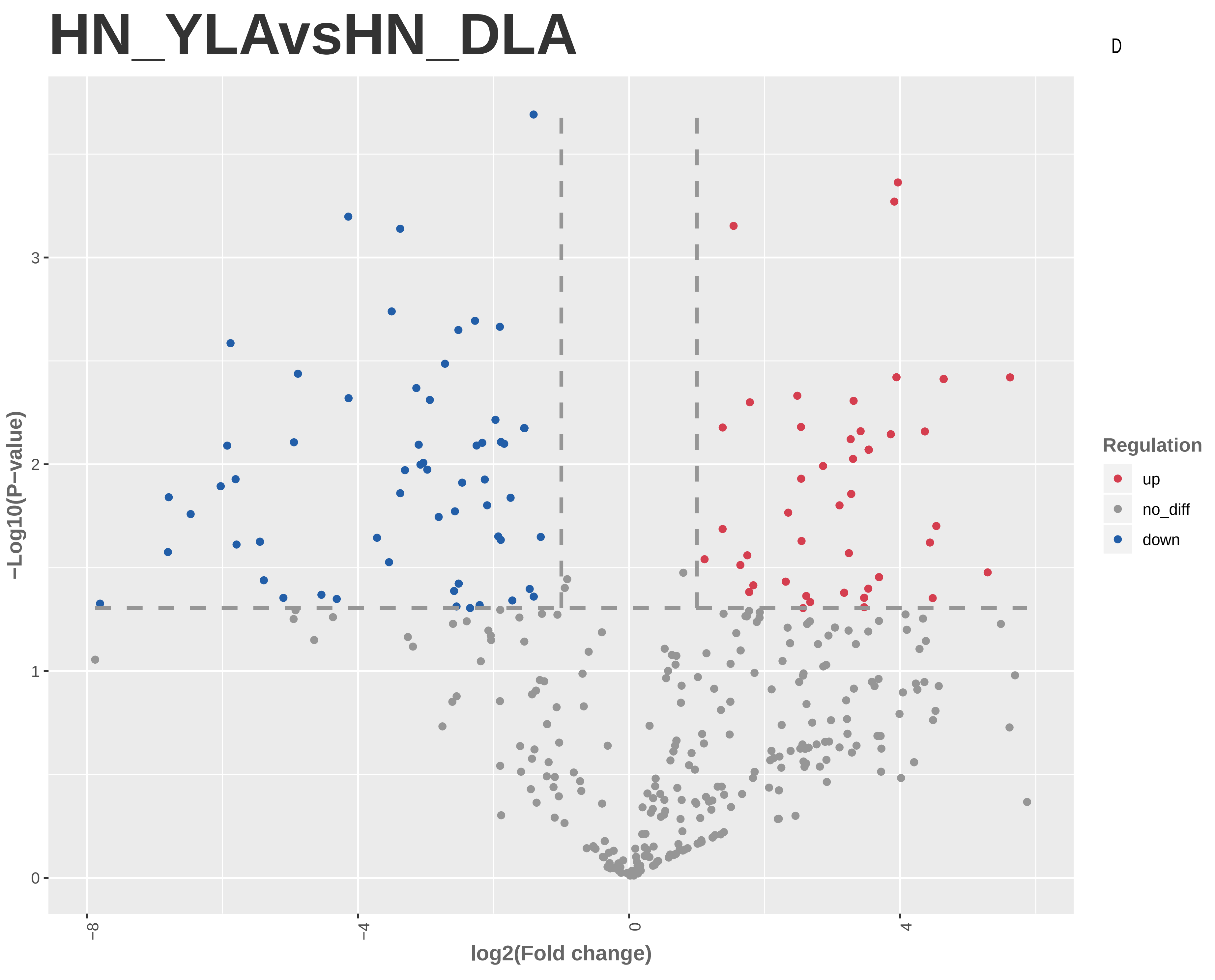
HN_YLAvsHN_DLA
D
−Log10(P−value)
log2(Fold change)
Regulation
up
no_diff
down
3
2
1
0
−8
−4
0
4

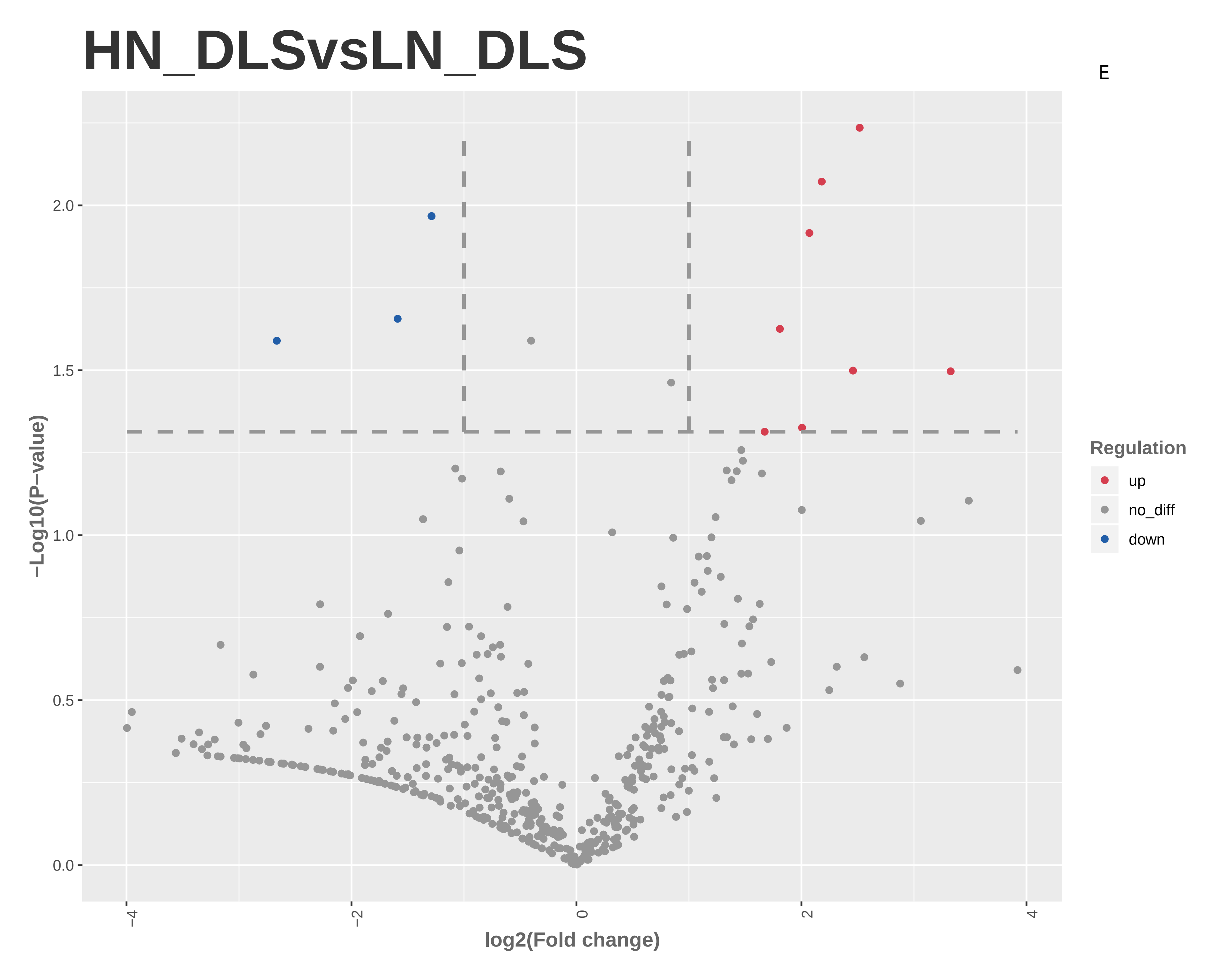

HN_DLSvsLN_DLS
E
−Log10(P−value)
2.0
1.5
1.0
0.5
0.0
−4
−2
0
2
4
log2(Fold change)
Regulation
up
no_diff
down

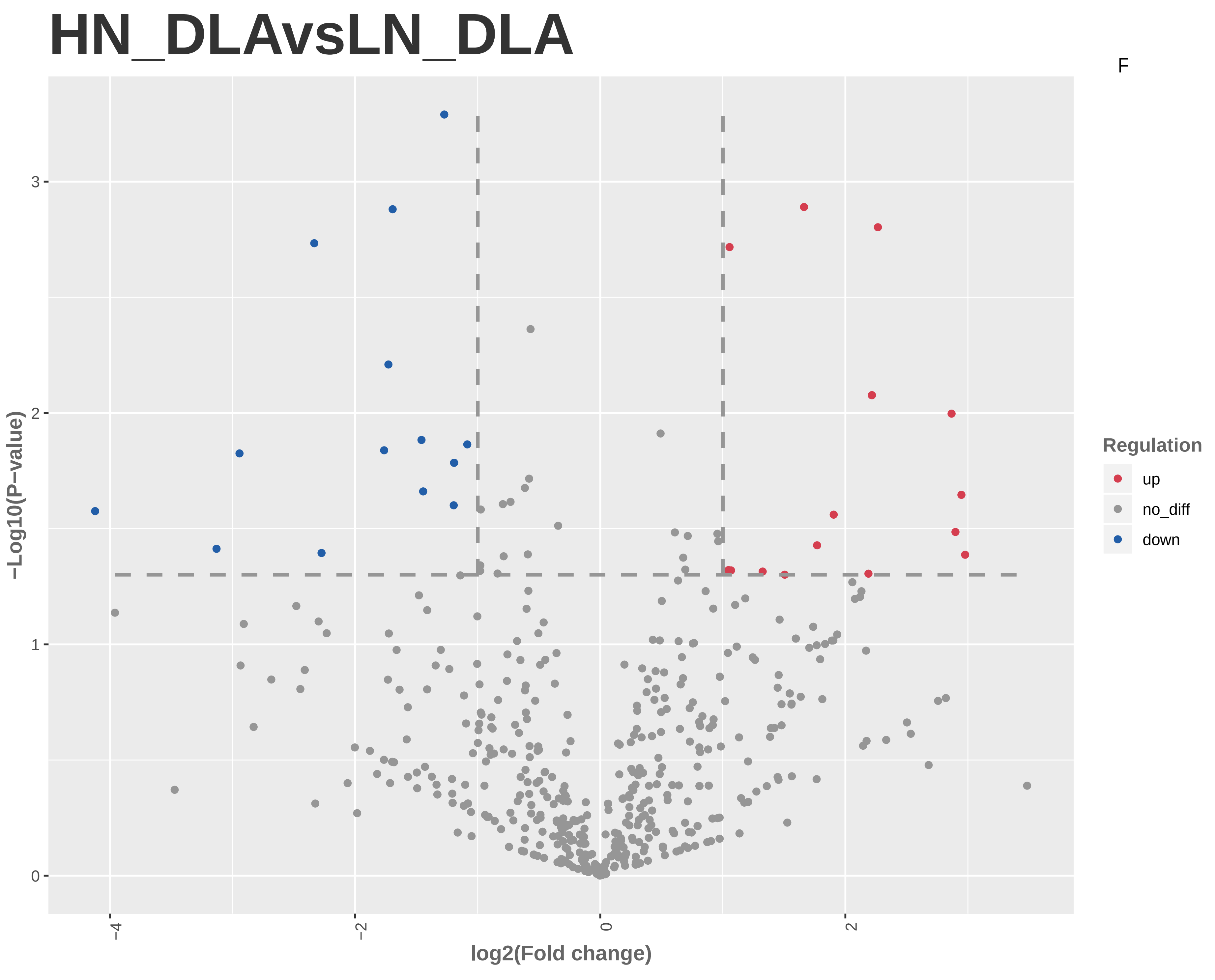
HN_DLAvsLN_DLA
F
Regulation
up
no_diff
down
−Log10(P−value)
log2(Fold change)
3
2
1
0
−4
−2
0
2

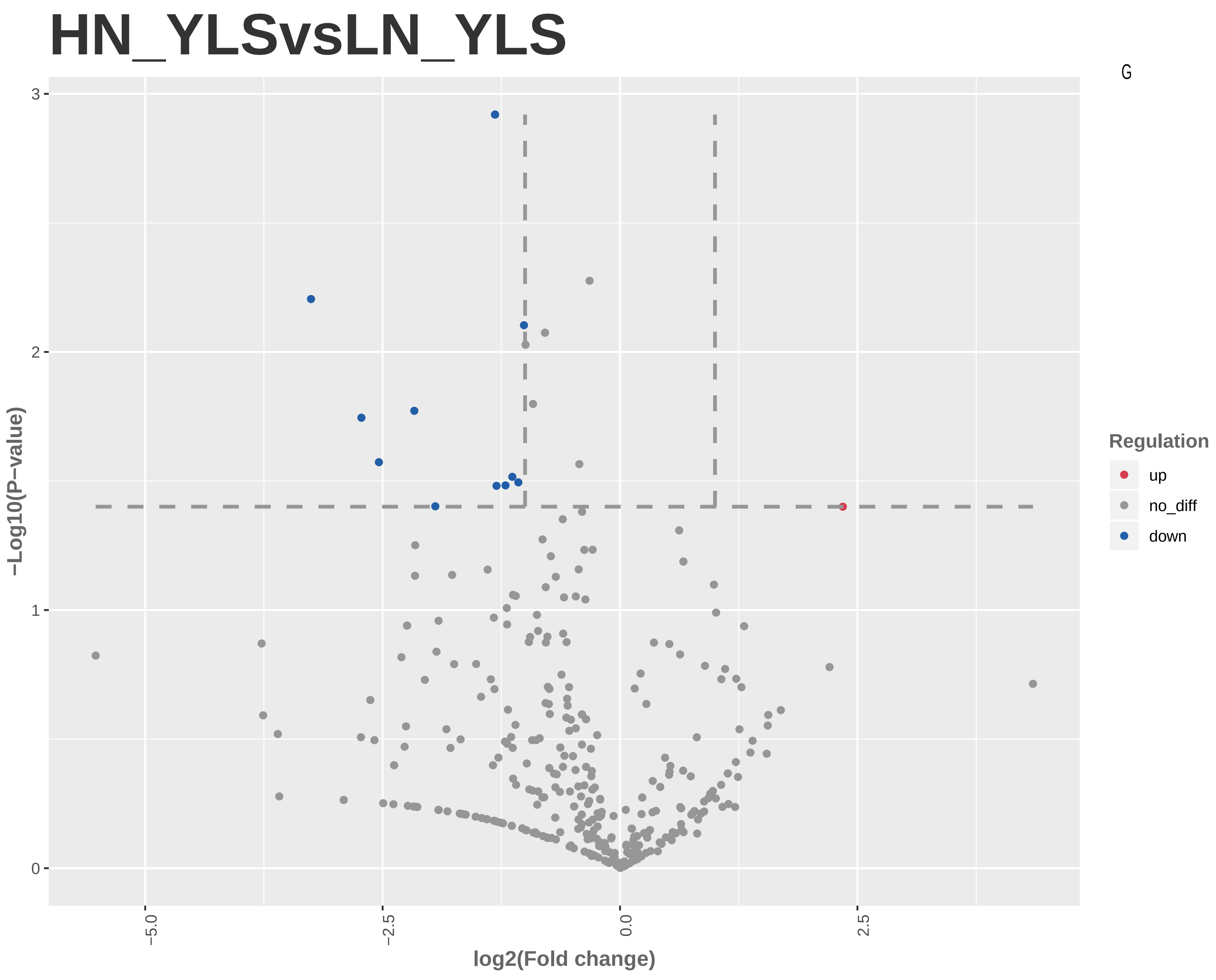

HN_YLSvsLN_YLS
G
3
2
1
0
−Log10(P−value)
−5.0
−2.5
0.0
2.5
log2(Fold change)
Regulation
up
no_diff
down

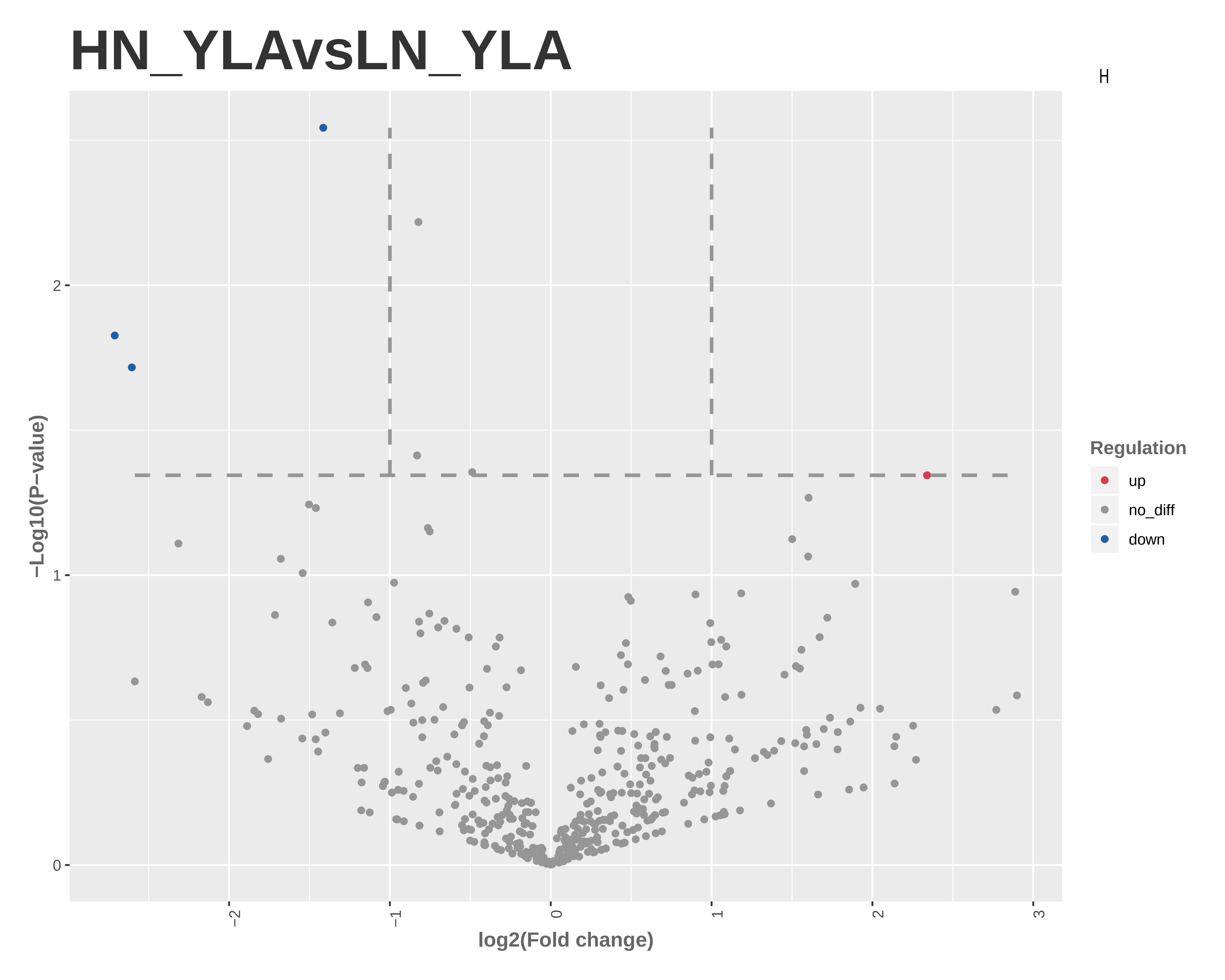

HN_YLAvsLN_YLA
H
−Log10(P−value)
2
1
0
−2
−1
0
1
2
3
log2(Fold change)
Regulation
up
no_diff
down

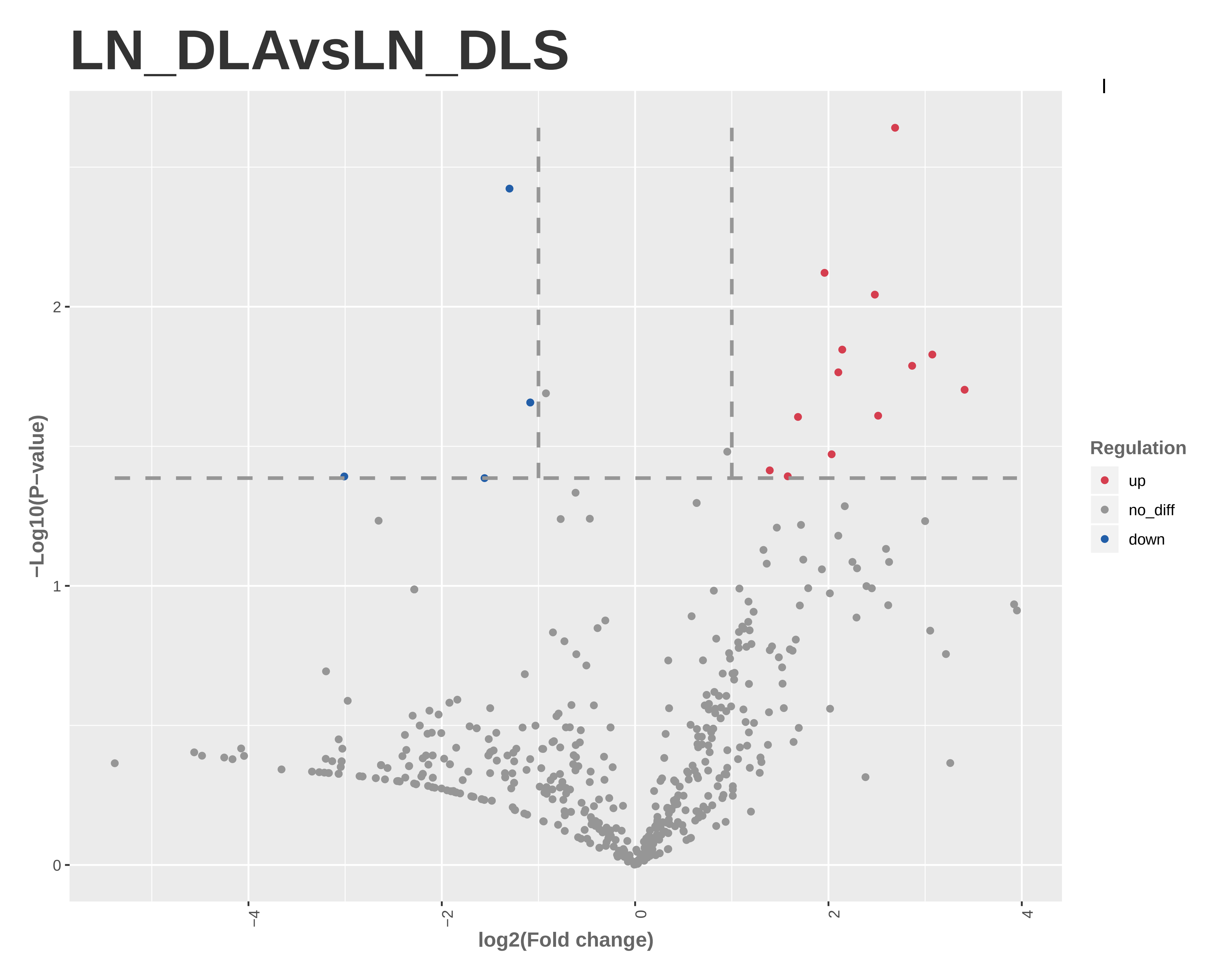
LN_DLAvsLN_DLS
−Log10(P−value)
log2(Fold change)
Regulation
up
no_diff
down
2
1
0
−4
−2
0
2
4

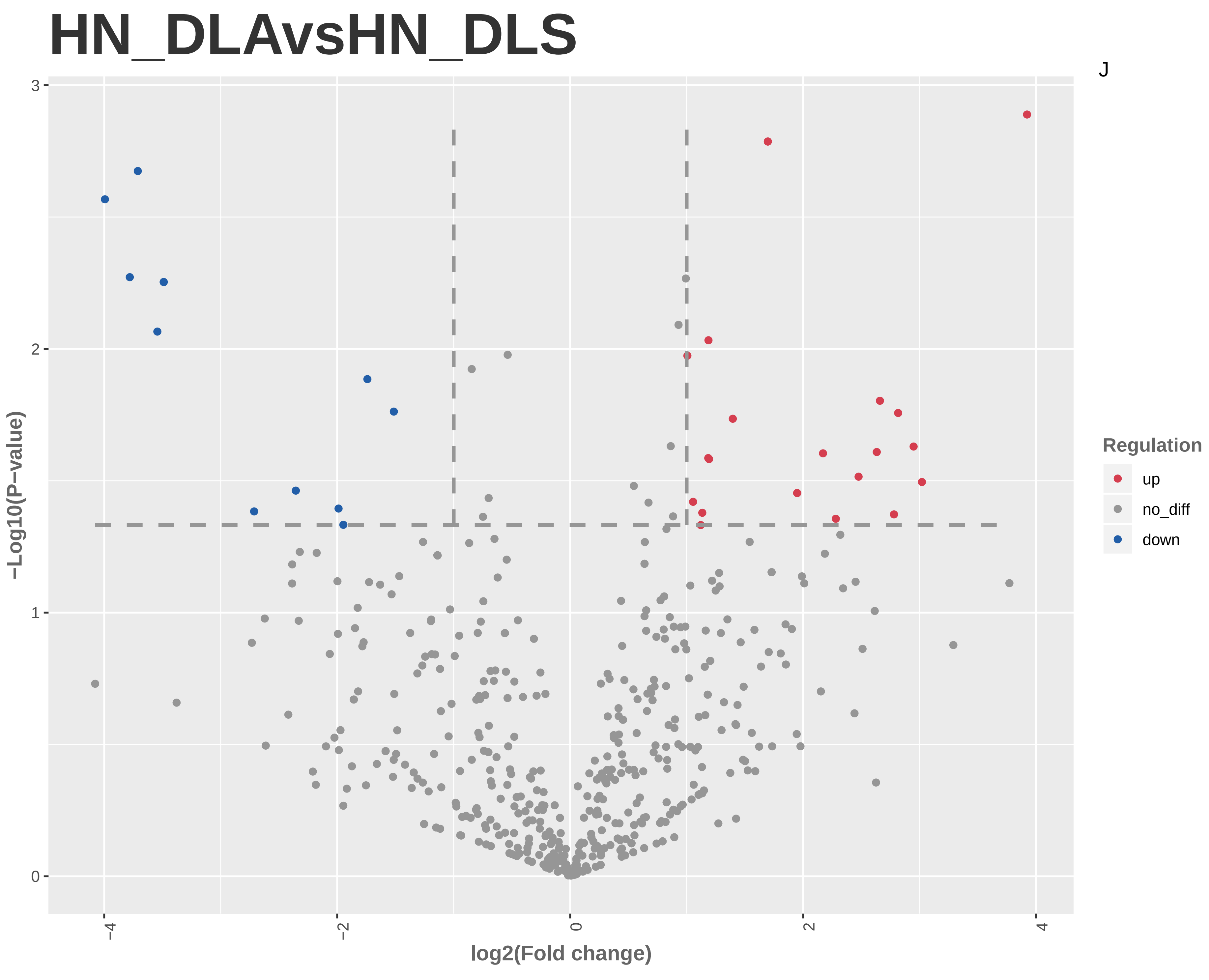
HN_DLAvsHN_DLS
J
−Log10(P−value)
log2(Fold change)
Regulation
up
no_diff
down
3
2
1
0
−4
−2
0
2
4

LN_YLAvsLN_YLS

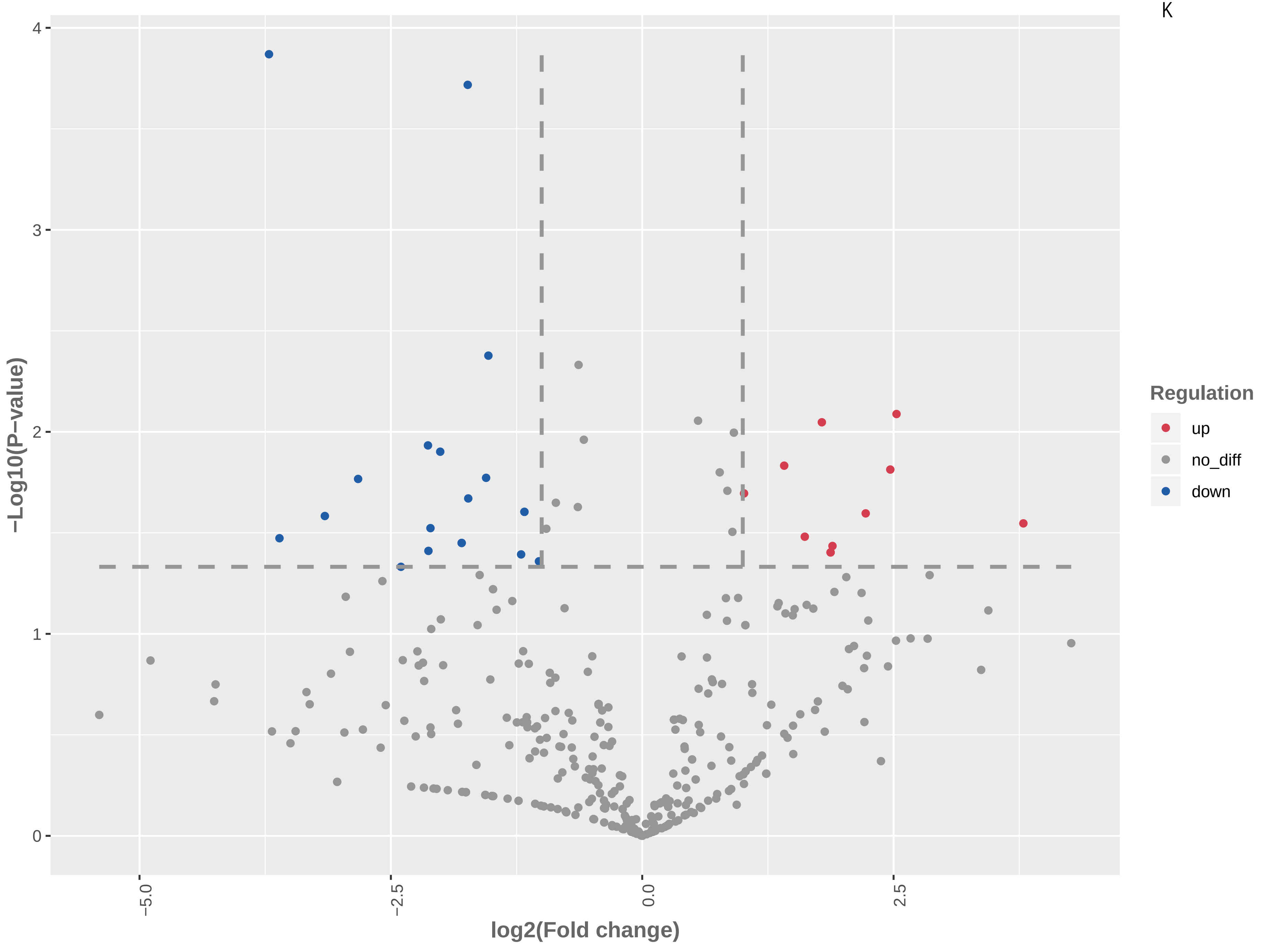

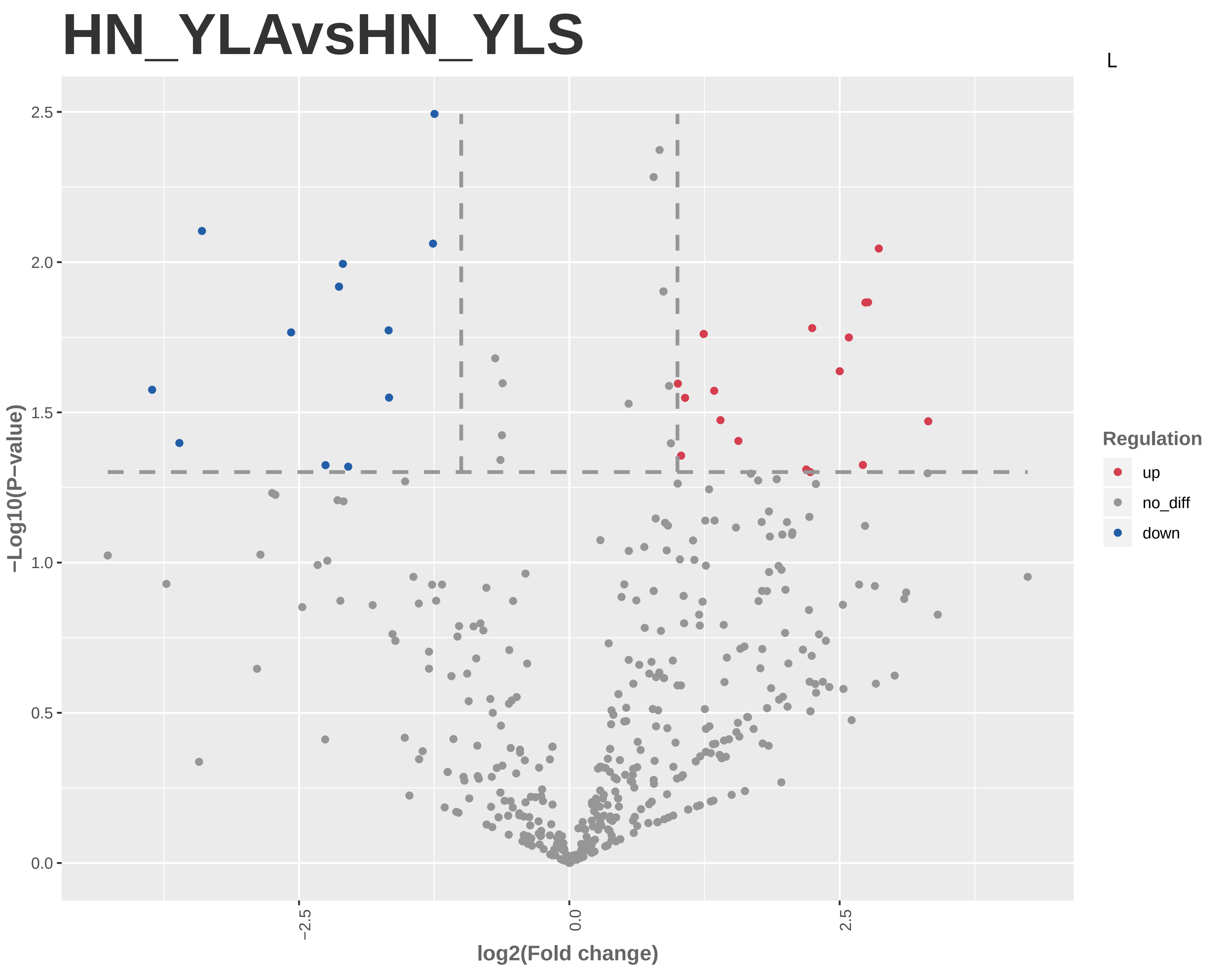
HN_YLAvsHN_YLS
L
−Log10(P−value)
2.5
2.0
1.5
1.0
0.5
0.0
−2.5
0.0
2.5
log2(Fold change)
Regulation
up
no_diff
down

LN_YRSvsLN_DRS

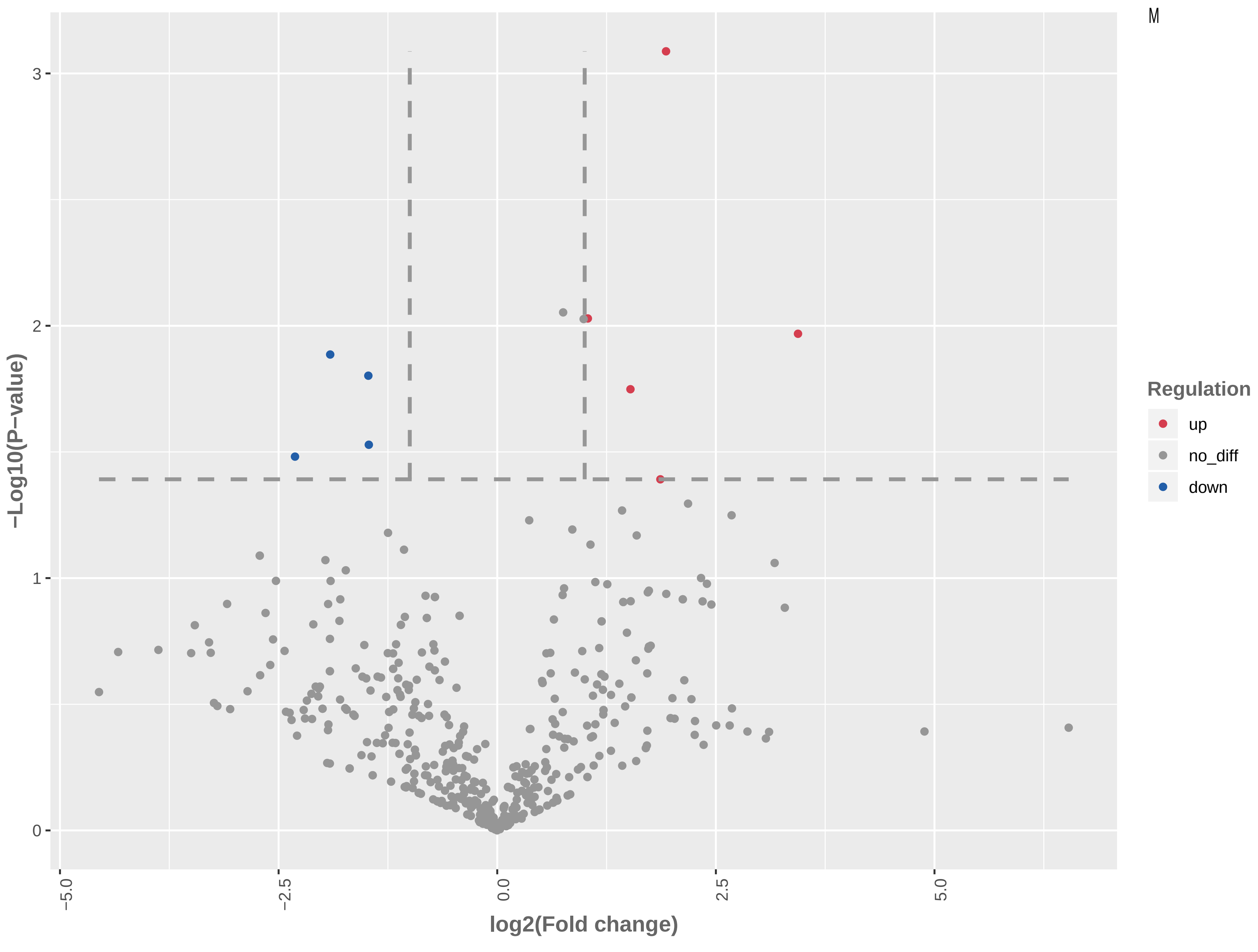

LN_YRAvsLN_DRA

N

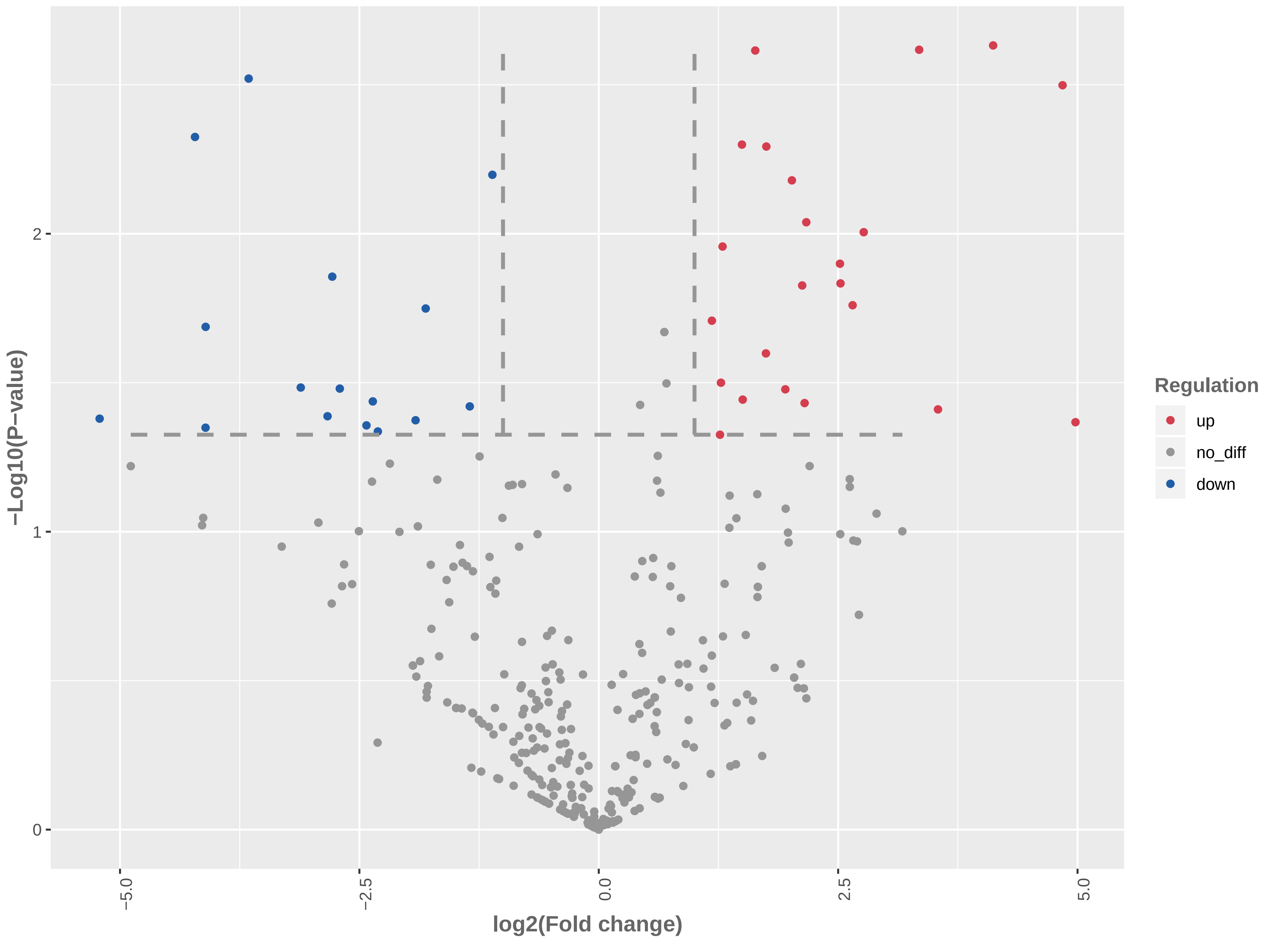

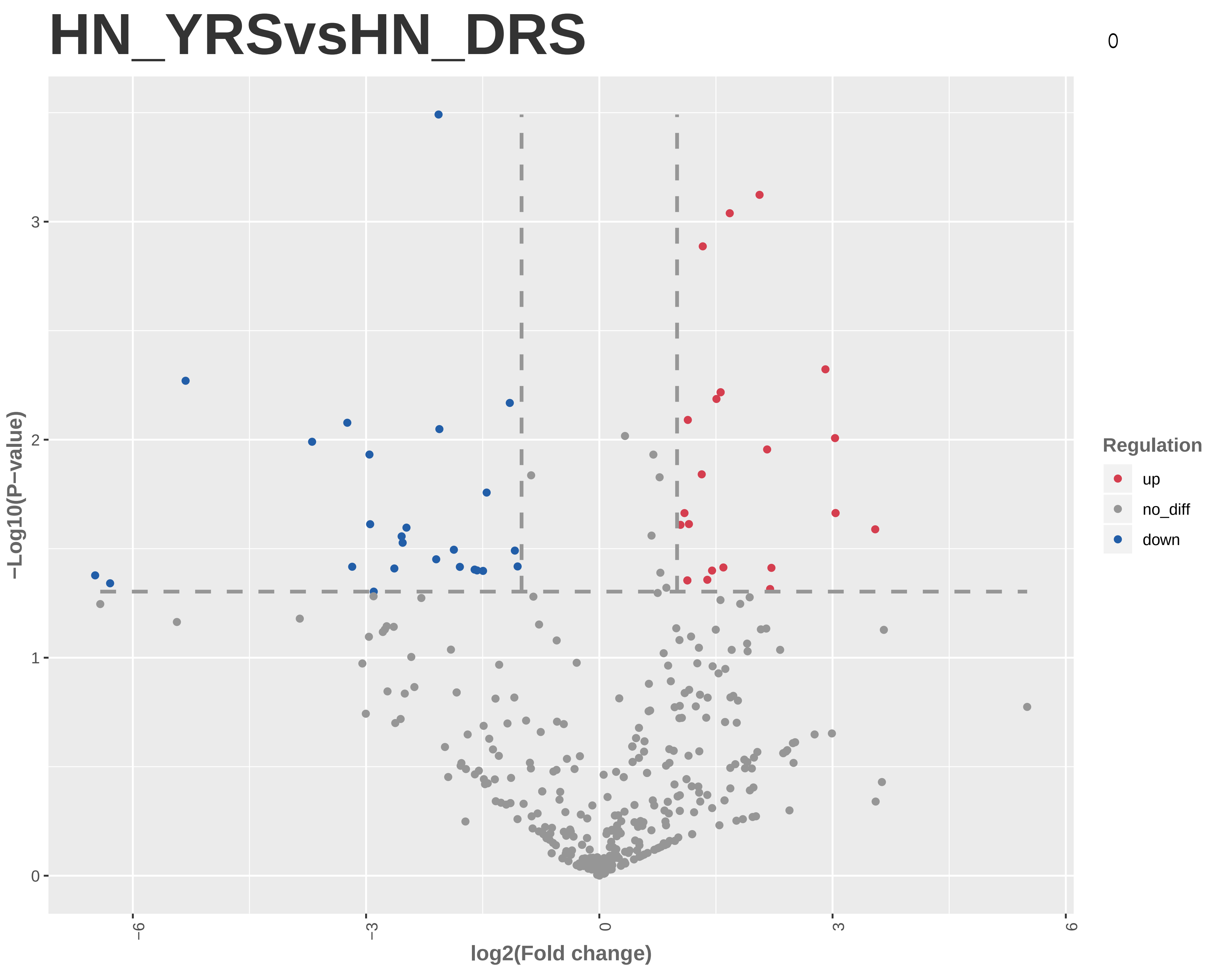
HN_YRSvsHN_DRS
0
−Log10(P−value)
log2(Fold change)
3
2
1
0
−6
−3
0
3
6
Regulation
up
no_diff
down

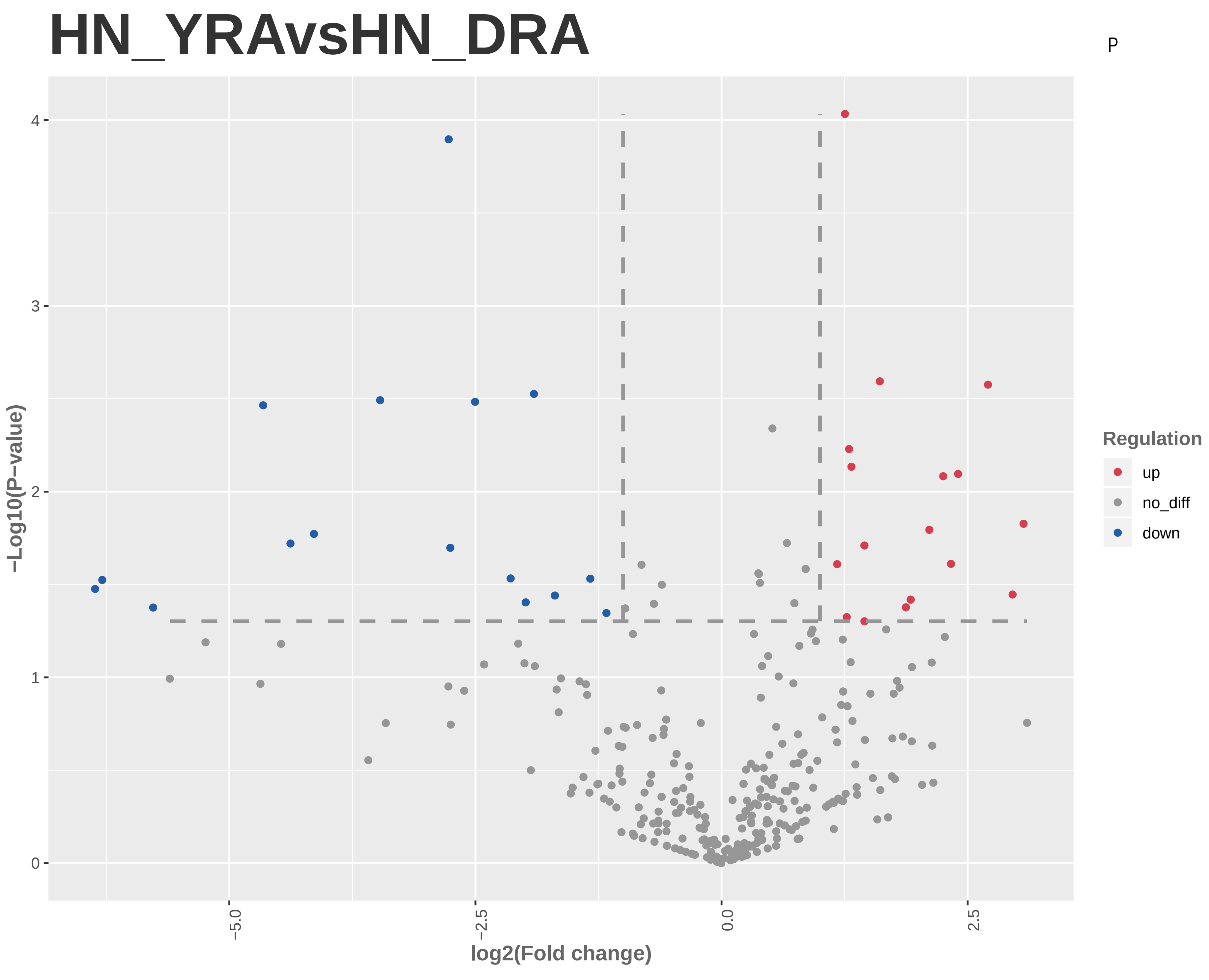

HN_YRAvsHN_DRA
P
−Log10(P−value)
log2(Fold change)
4
3
2
1
0
−5.0
−2.5
0.0
2.5
Regulation
up
no_diff
down

HN_DRSvsLN_DRS

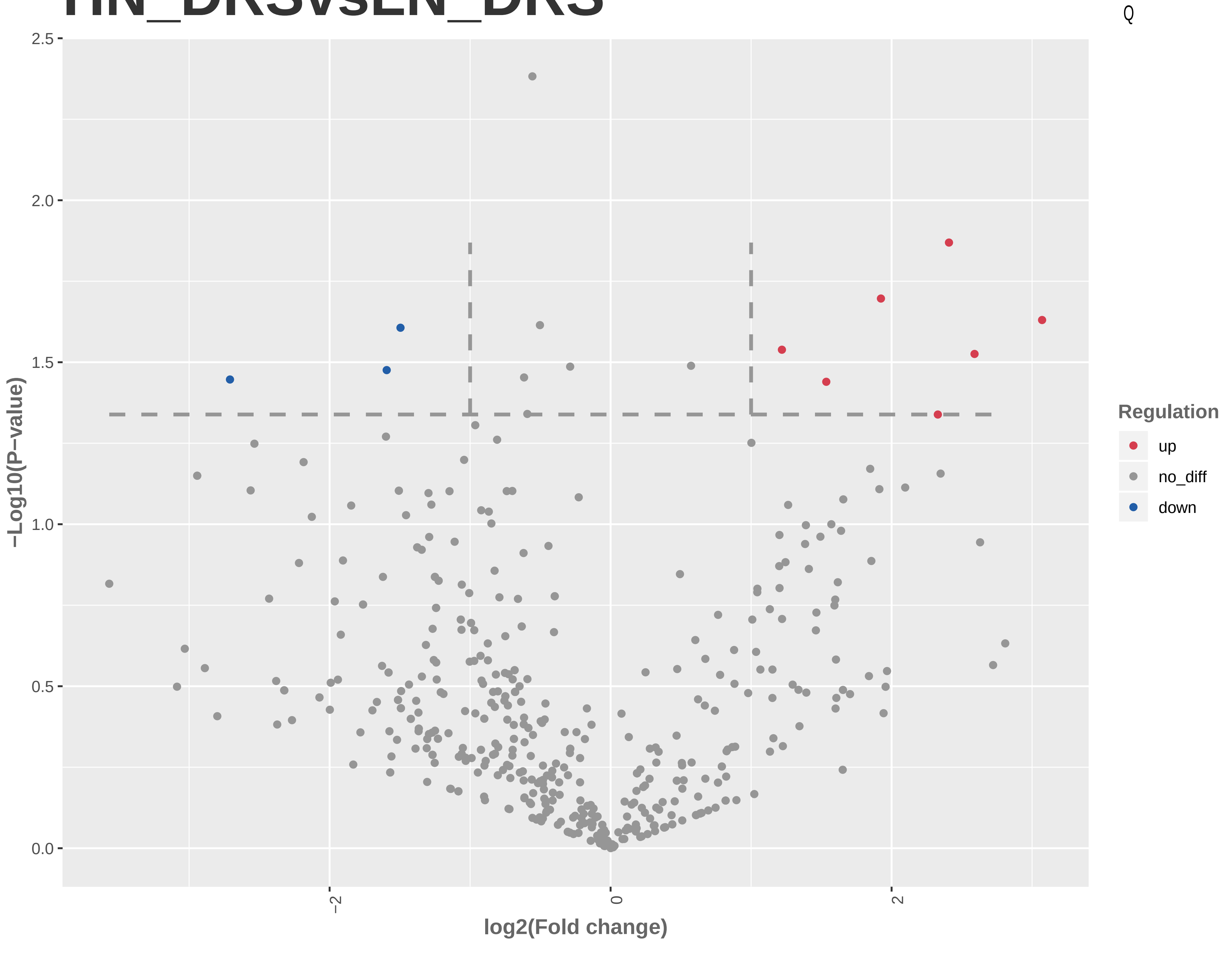

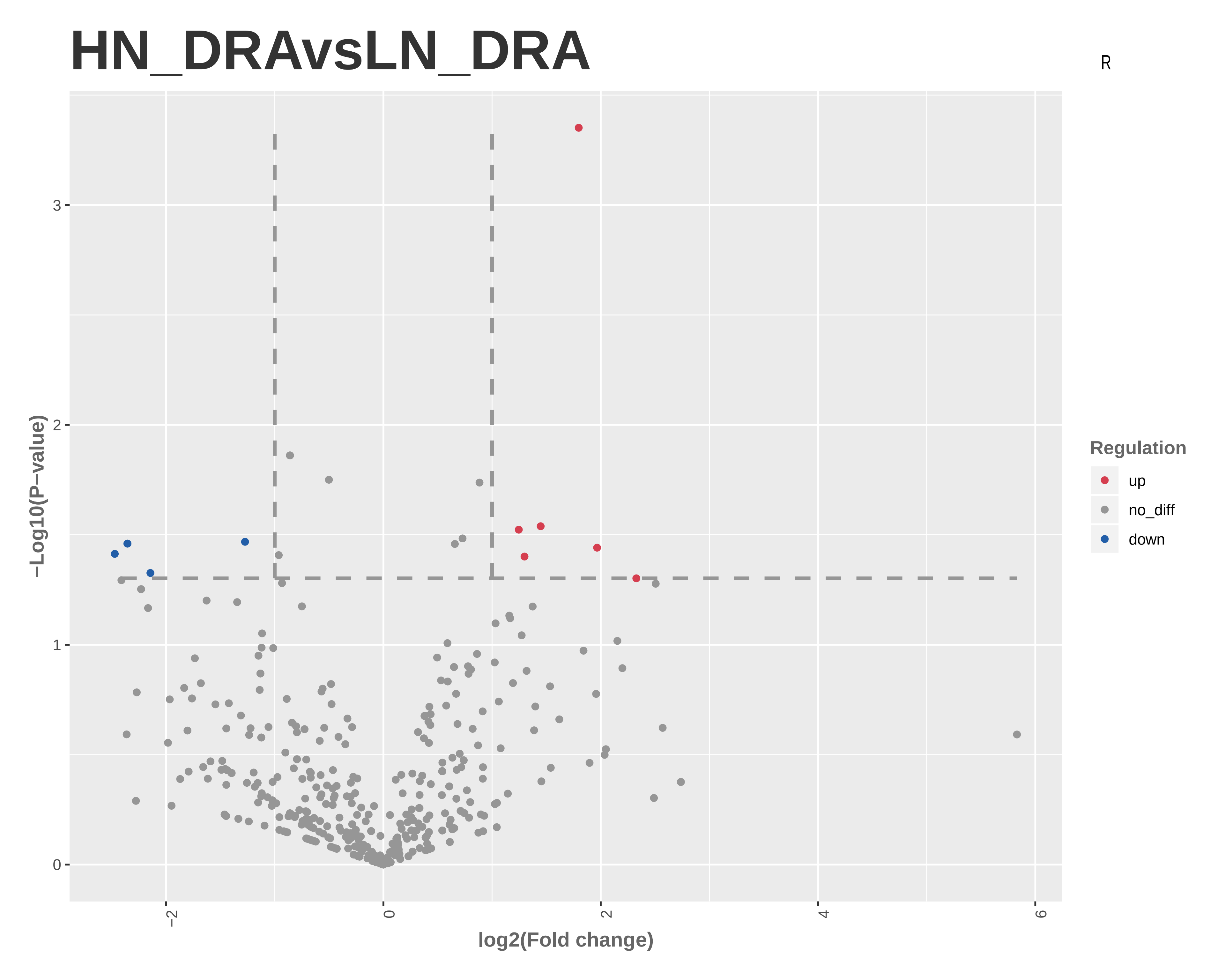

HN_DRAvsLN_DRA
R
−Log10(P−value)
log2(Fold change)
Regulation
up
no_diff
down

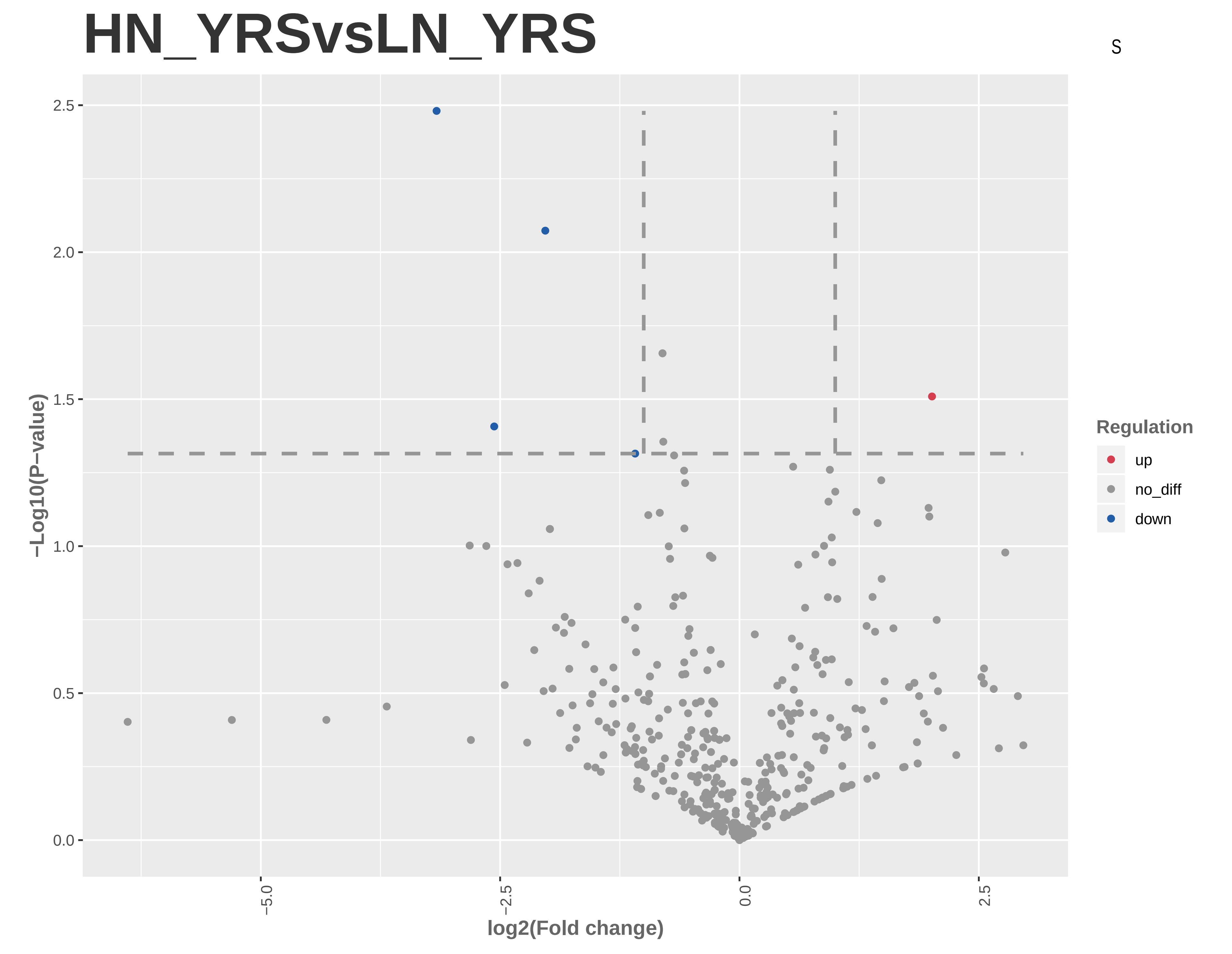

HN_YRSvsLN_YRS
S
−Log10(P−value)
2.5
2.0
1.5
1.0
0.5
0.0
−5.0
−2.5
0.0
2.5
log2(Fold change)
Regulation
up
no_diff
down

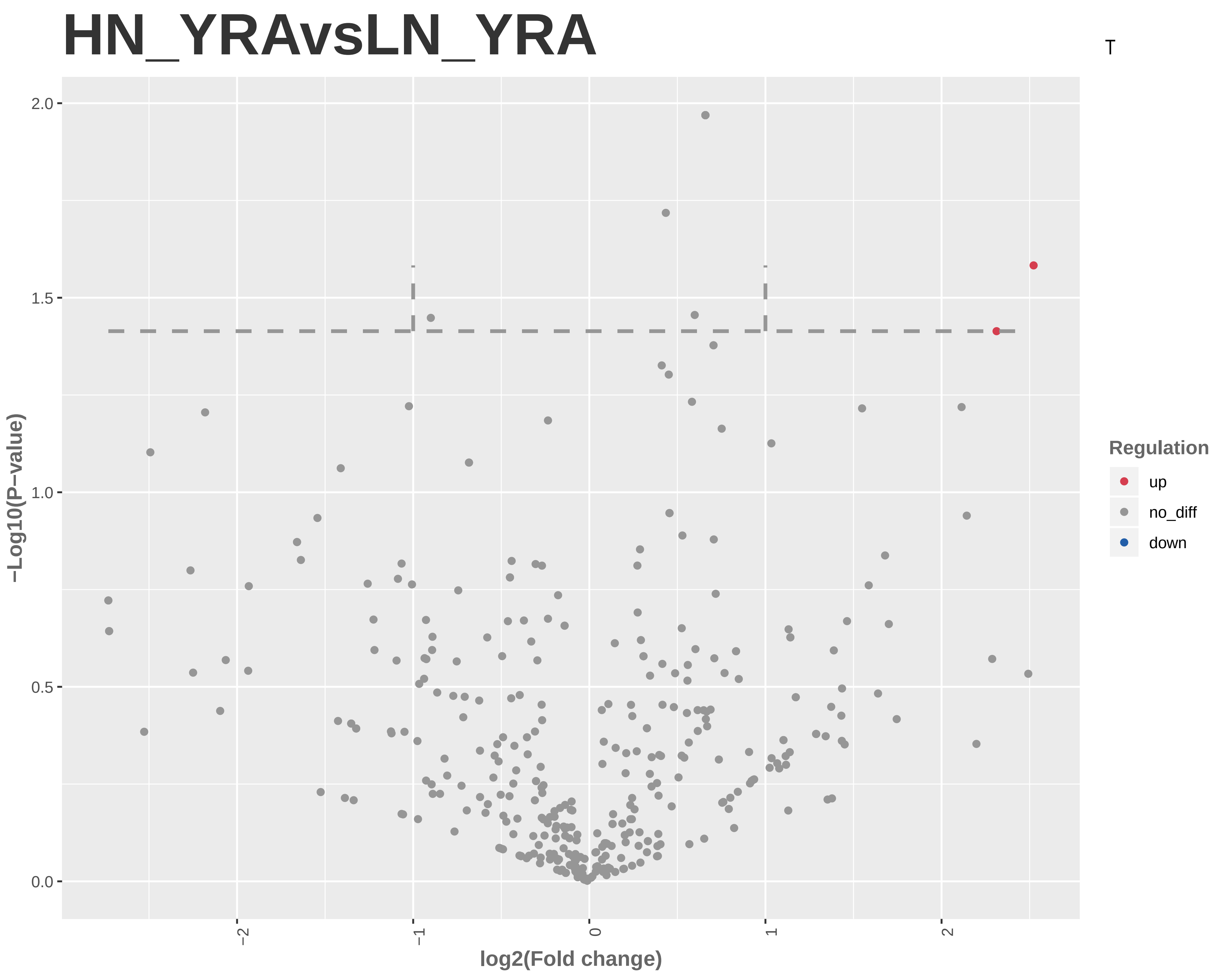
HN_YRAvsLN_YRA
T
−Log10(P−value)
2.0
1.5
1.0
0.5
0.0
−2
−1
0
1
2
log2(Fold change)
Regulation
up
no_diff
down

LN_DRAvsLN_DRS

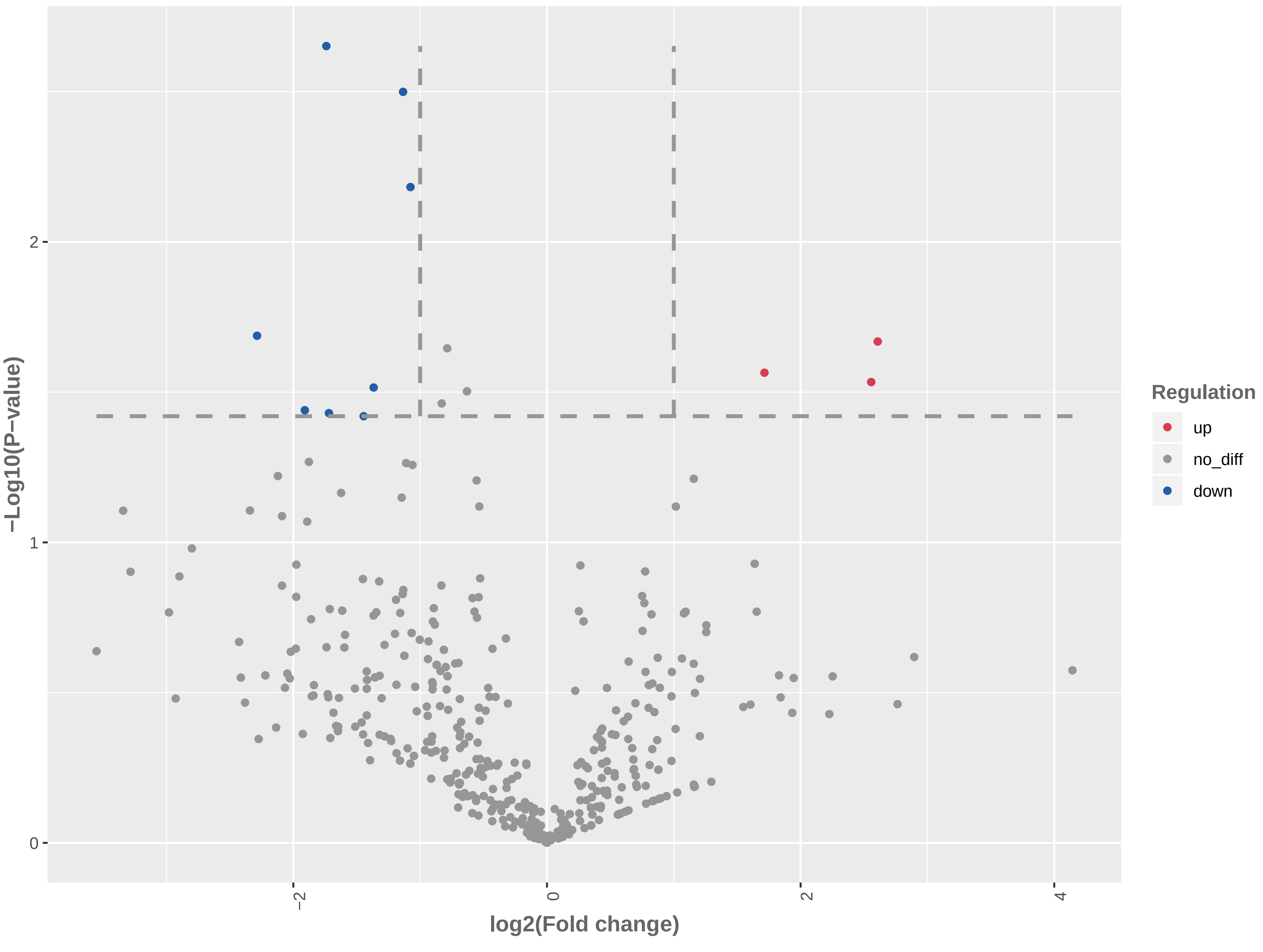

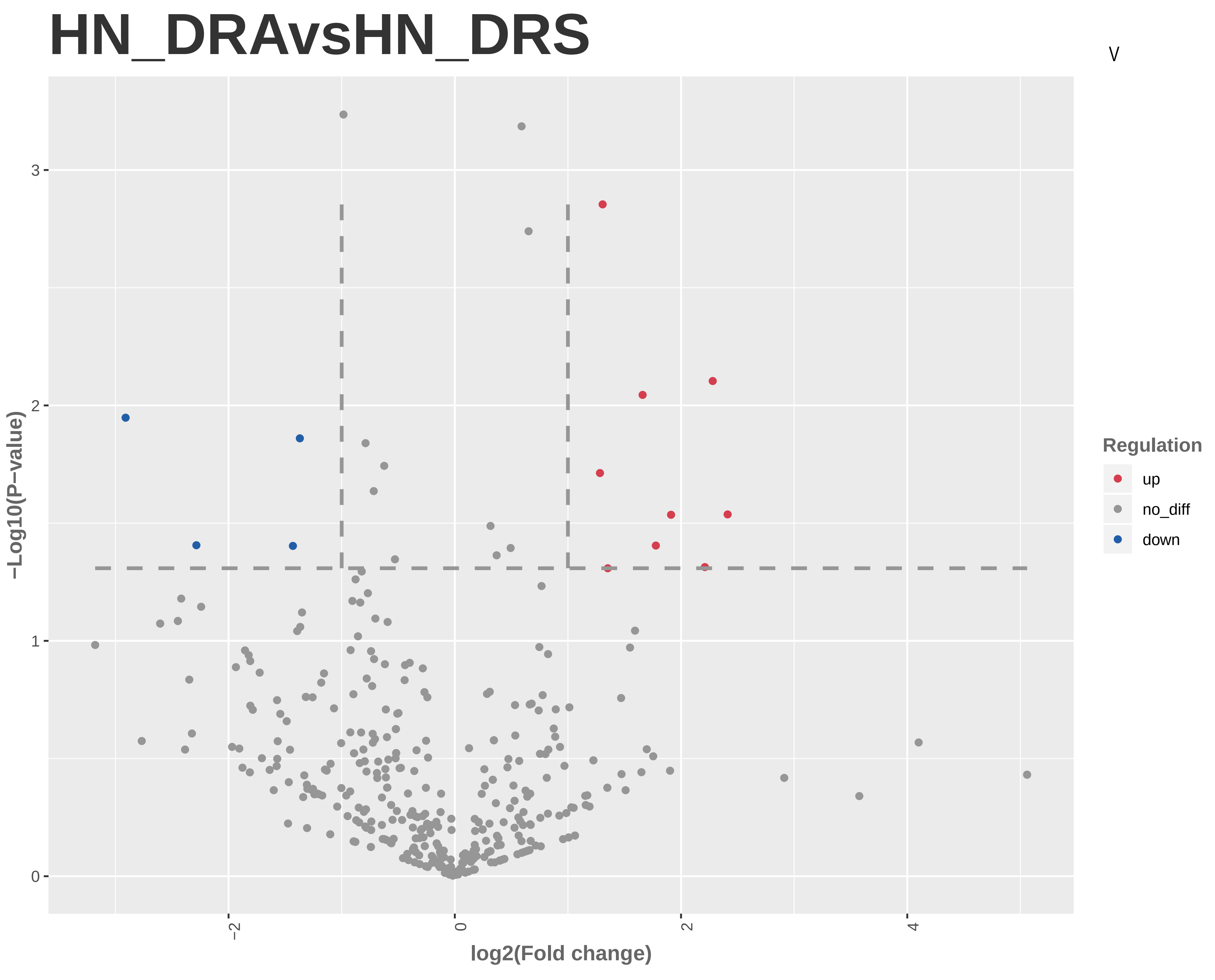

HN_DRAvsHN_DRS
−Log10(P−value)
log2(Fold change)
Regulation
up
no_diff
down

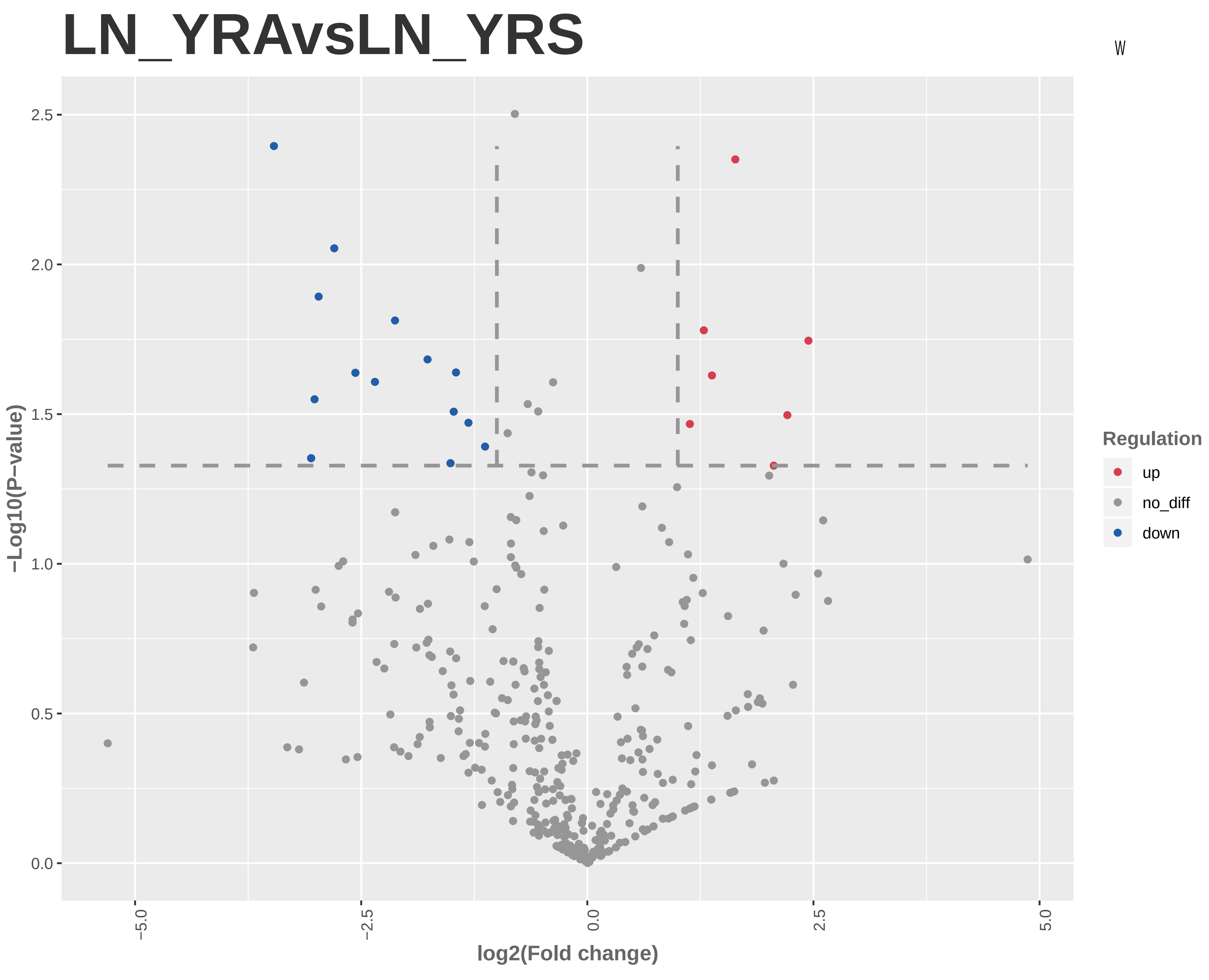

LN_YRAvsLN_YRS
W
−Log10(P−value)
2.5
2.0
1.5
1.0
0.5
0.0
−5.0
−2.5
0.0
2.5
5.0
log2(Fold change)
Regulation
up
no_diff
down

HN_YRAvsHN_YRS

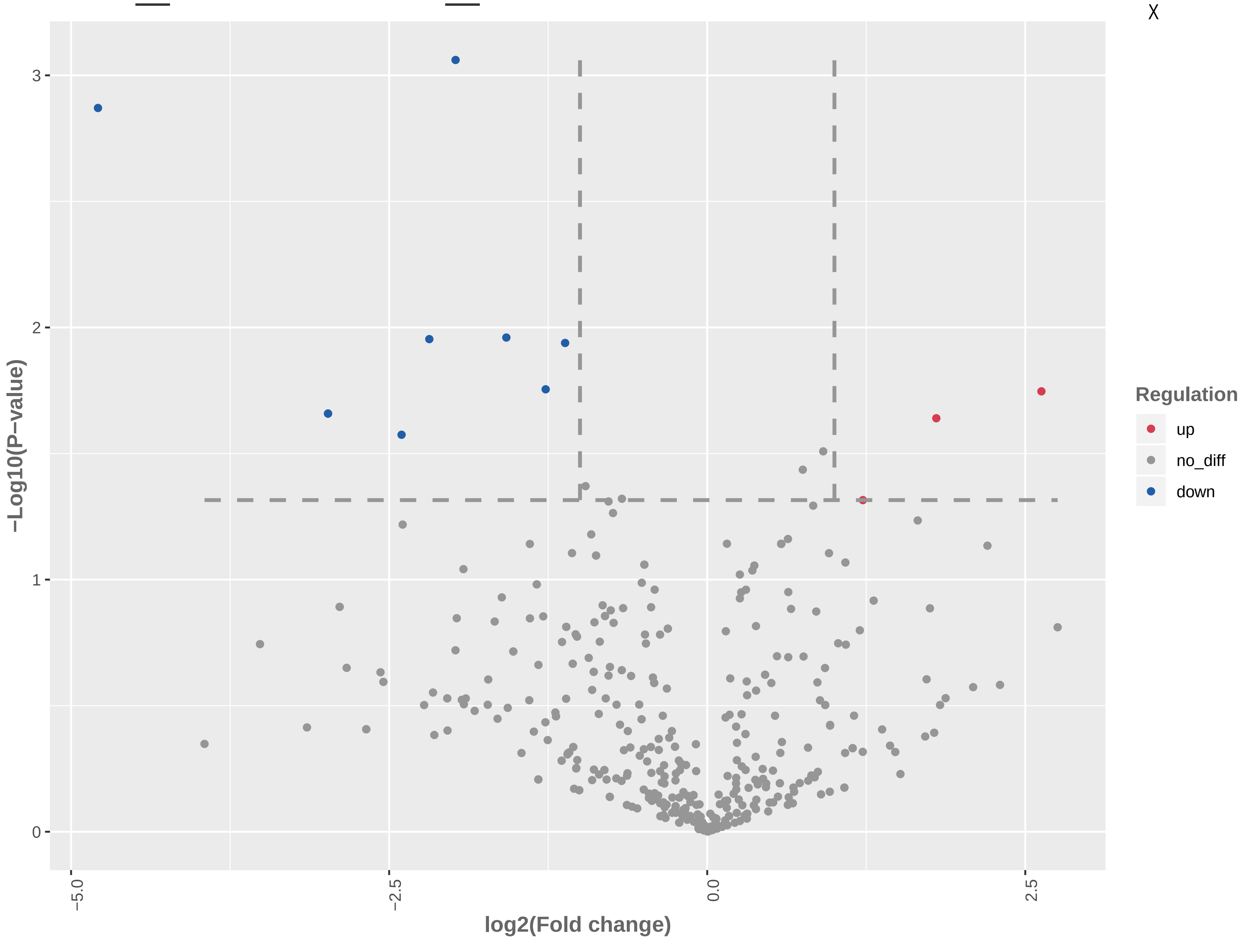

Supplement: Supplementary file 2 — Additional file 2: Figure 1. Analysis of variability among groups of differential miRNAsHN: excessive N application; LN: no N application; Y: Yanshu4; D: Atlantic;S: seedling stage; A: budding stage (A) LN_YLSvsLN_DLS (B) LN_YLAvsLN_DLA (C) HN_YLSvsHN_DLS. (D)HN_YLAvsHN_DLA (E) HN_DLSvsLN_DLS (F) HN_DLAvsLN_DLA. (G) HN_YLSvsLN_YLS (H)HN_YLAvsLN_YLA (I) LN_DLAvsLN_DLS. (J)HN_DLAvsHN_DLS (K)LN_YLAvsLN_YLS (L)HN_YLAvsHN_YLS.(M)LN_YRSvsLN_DRS (N)LN_YRAvsLN_DRA (O)HN_YRSvsHN_DRS. (P)HN_YRAvsHN_DRA (Q)HN_DRSvsLN_DRS(R)HN_DRAvsLN_DRA. (S)HN_YRSvsLN_YRS (T)HN_YRAvsLN_YRA (U)LN_DRAvsLN_DRS. (V)HN_DRAvsHN_DRS(W)LN_YRAvsLN_YRS (X)HN_YRAvsHN_YRS. Using the values of log2(fold change) as the horizontalcoordinate and -log10(p-value) as the vertical coordinate, volcanoplots were constructed for all miRNAs during differential expression analysis.The horizontal coordinate represents the fold change in the differentialexpression of miRNAs in different samples, and the vertical coordinaterepresents the statistical significance of the difference in the change inexpression levels of miRNAs. The red color represents up-regulatedsignificantly differentially expressed genes, the blue color representsdown-regulated significantly differentially expressed genes, and the grey dots representnon-significant differentially expressed genes. Figure 2. Comparison between groups of differential miRNAs. HN: excess N; LN:no N; Y: Yanshu4; D: Atlantic; S: seedling stage; A: budding stage. Thehorizontal coordinates indicate the data obtained after the comparison ofgroups, and the vertical coordinates indicate the number of up- anddown-regulated miRNAs. The red color represents up-regulated miRNAs, the bluecolor represents down-regulated miRNAs, and the numbers represent the number ofup- and down-regulated miRNAs. Figure 3. Results of clustering analysis ofdifferential miRNAs. HN: excess N; LN: no N; Y: Yanshu4; D: Atlantic; S: seedling;A: bud onset. (A)LN_YLSvsLN_DLS (B)LN_YLAvsLN_DLA (C)HN_YLSvsHN_DLS. [file 12870_2022_3866_MOESM2_ESM.zip › Appendix B Figure 1.pdf]
