# Supplementary figures and images for: Screening of differentially expressed microRNAs and target genes in two potato varieties under nitrogen stress
Source: BMC Plant Biol. 2022 Oct 8;22:478. doi: 10.1186/s12870-022-03866-5 (PMC9547441; doi:10.1186/s12870-022-03866-5)

# Differentially expressed mirnas in different groups

up-regulated down-regulated

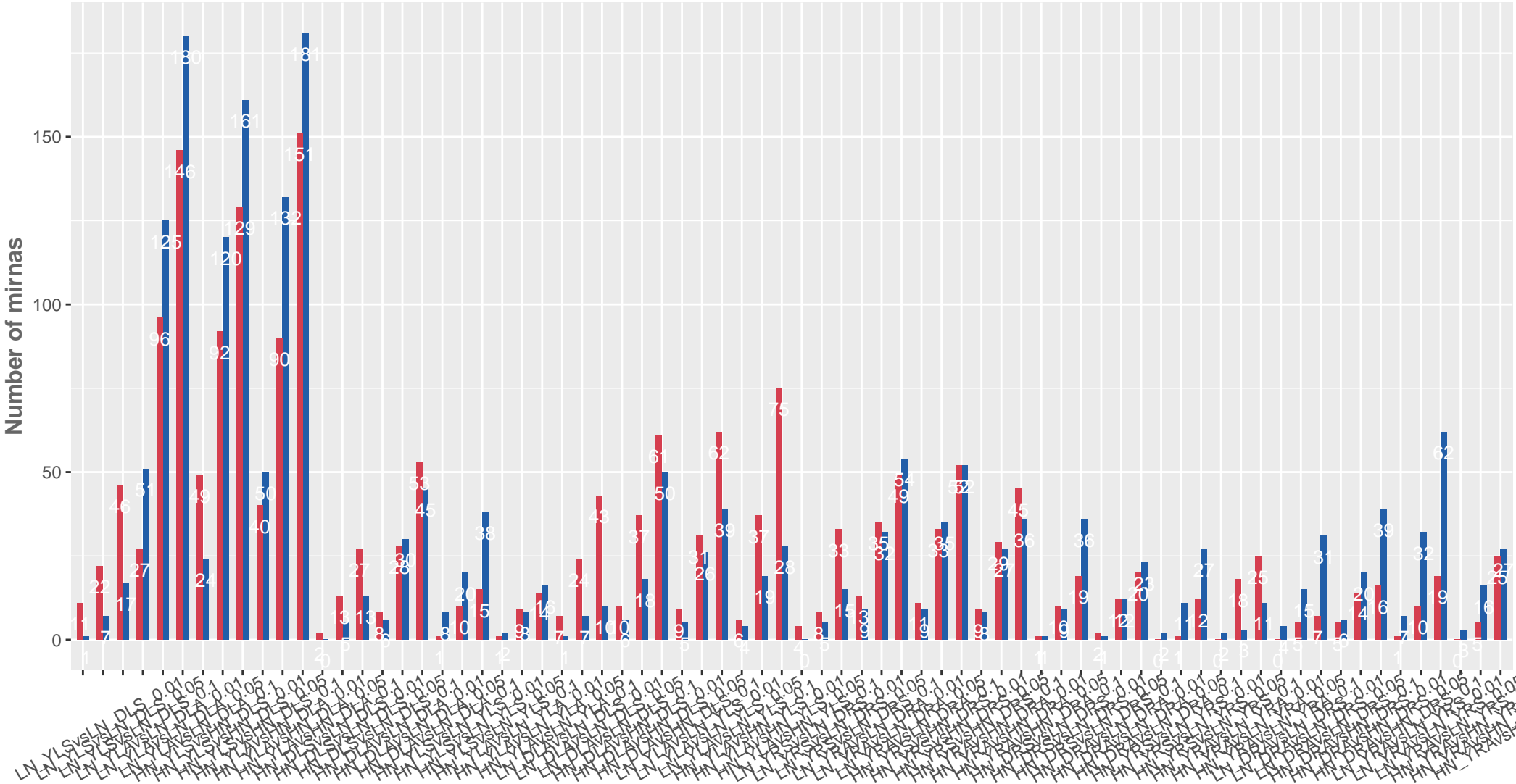

Supplement: Supplementary file 2 — Additional file 2: Figure 1. Analysis of variability among groups of differential miRNAsHN: excessive N application; LN: no N application; Y: Yanshu4; D: Atlantic;S: seedling stage; A: budding stage (A) LN_YLSvsLN_DLS (B) LN_YLAvsLN_DLA (C) HN_YLSvsHN_DLS. (D)HN_YLAvsHN_DLA (E) HN_DLSvsLN_DLS (F) HN_DLAvsLN_DLA. (G) HN_YLSvsLN_YLS (H)HN_YLAvsLN_YLA (I) LN_DLAvsLN_DLS. (J)HN_DLAvsHN_DLS (K)LN_YLAvsLN_YLS (L)HN_YLAvsHN_YLS.(M)LN_YRSvsLN_DRS (N)LN_YRAvsLN_DRA (O)HN_YRSvsHN_DRS. (P)HN_YRAvsHN_DRA (Q)HN_DRSvsLN_DRS(R)HN_DRAvsLN_DRA. (S)HN_YRSvsLN_YRS (T)HN_YRAvsLN_YRA (U)LN_DRAvsLN_DRS. (V)HN_DRAvsHN_DRS(W)LN_YRAvsLN_YRS (X)HN_YRAvsHN_YRS. Using the values of log2(fold change) as the horizontalcoordinate and -log10(p-value) as the vertical coordinate, volcanoplots were constructed for all miRNAs during differential expression analysis.The horizontal coordinate represents the fold change in the differentialexpression of miRNAs in different samples, and the vertical coordinaterepresents the statistical significance of the difference in the change inexpression levels of miRNAs. The red color represents up-regulatedsignificantly differentially expressed genes, the blue color representsdown-regulated significantly differentially expressed genes, and the grey dots representnon-significant differentially expressed genes. Figure 2. Comparison between groups of differential miRNAs. HN: excess N; LN:no N; Y: Yanshu4; D: Atlantic; S: seedling stage; A: budding stage. Thehorizontal coordinates indicate the data obtained after the comparison ofgroups, and the vertical coordinates indicate the number of up- anddown-regulated miRNAs. The red color represents up-regulated miRNAs, the bluecolor represents down-regulated miRNAs, and the numbers represent the number ofup- and down-regulated miRNAs. Figure 3. Results of clustering analysis ofdifferential miRNAs. HN: excess N; LN: no N; Y: Yanshu4; D: Atlantic; S: seedling;A: bud onset. (A)LN_YLSvsLN_DLS (B)LN_YLAvsLN_DLA (C)HN_YLSvsHN_DLS. [file 12870_2022_3866_MOESM2_ESM.zip › Appendix B Figure 2.pdf]

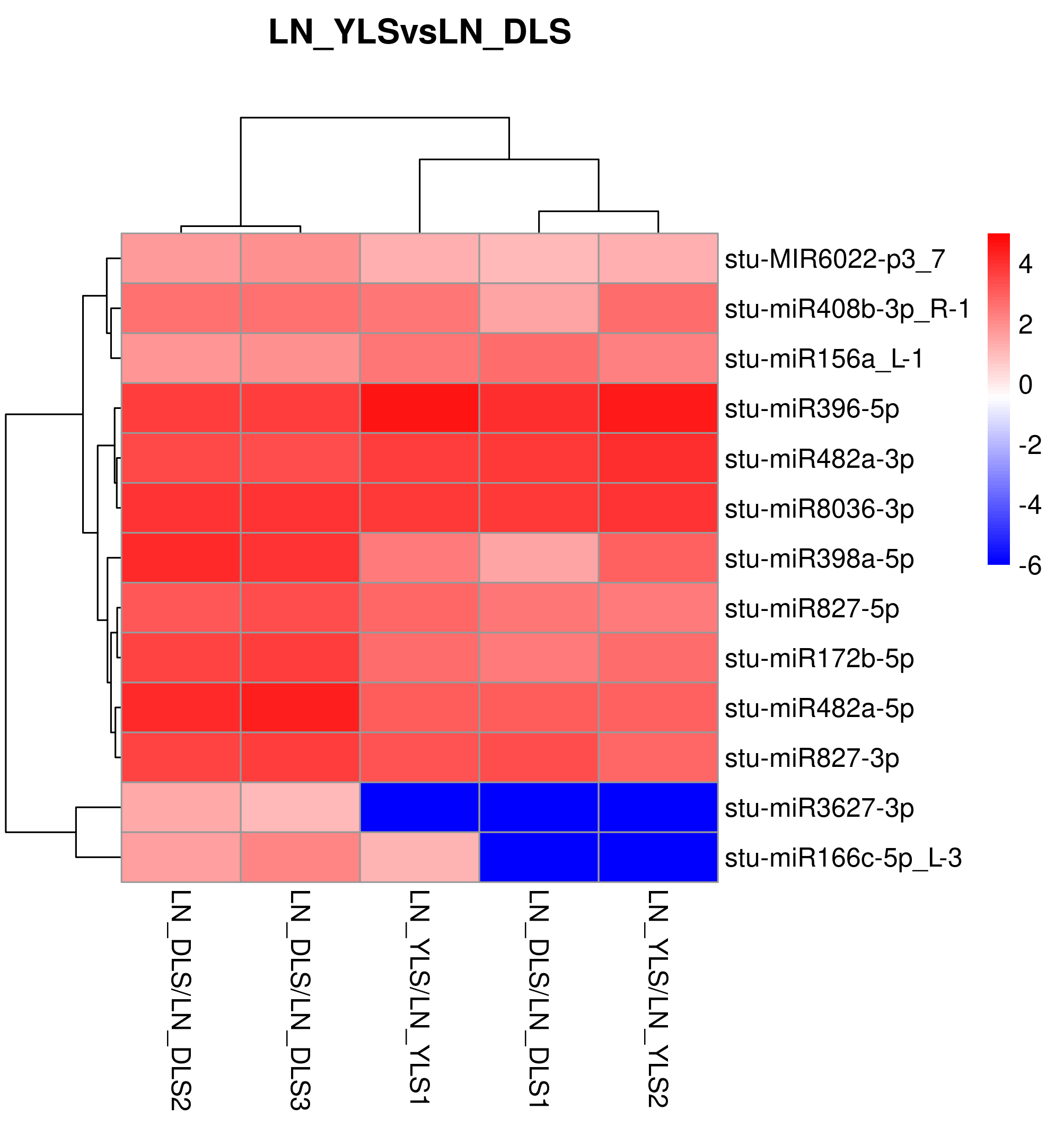

Supplement: Supplementary file 2 — Additional file 2: Figure 1. Analysis of variability among groups of differential miRNAsHN: excessive N application; LN: no N application; Y: Yanshu4; D: Atlantic;S: seedling stage; A: budding stage (A) LN_YLSvsLN_DLS (B) LN_YLAvsLN_DLA (C) HN_YLSvsHN_DLS. (D)HN_YLAvsHN_DLA (E) HN_DLSvsLN_DLS (F) HN_DLAvsLN_DLA. (G) HN_YLSvsLN_YLS (H)HN_YLAvsLN_YLA (I) LN_DLAvsLN_DLS. (J)HN_DLAvsHN_DLS (K)LN_YLAvsLN_YLS (L)HN_YLAvsHN_YLS.(M)LN_YRSvsLN_DRS (N)LN_YRAvsLN_DRA (O)HN_YRSvsHN_DRS. (P)HN_YRAvsHN_DRA (Q)HN_DRSvsLN_DRS(R)HN_DRAvsLN_DRA. (S)HN_YRSvsLN_YRS (T)HN_YRAvsLN_YRA (U)LN_DRAvsLN_DRS. (V)HN_DRAvsHN_DRS(W)LN_YRAvsLN_YRS (X)HN_YRAvsHN_YRS. Using the values of log2(fold change) as the horizontalcoordinate and -log10(p-value) as the vertical coordinate, volcanoplots were constructed for all miRNAs during differential expression analysis.The horizontal coordinate represents the fold change in the differentialexpression of miRNAs in different samples, and the vertical coordinaterepresents the statistical significance of the difference in the change inexpression levels of miRNAs. The red color represents up-regulatedsignificantly differentially expressed genes, the blue color representsdown-regulated significantly differentially expressed genes, and the grey dots representnon-significant differentially expressed genes. Figure 2. Comparison between groups of differential miRNAs. HN: excess N; LN:no N; Y: Yanshu4; D: Atlantic; S: seedling stage; A: budding stage. Thehorizontal coordinates indicate the data obtained after the comparison ofgroups, and the vertical coordinates indicate the number of up- anddown-regulated miRNAs. The red color represents up-regulated miRNAs, the bluecolor represents down-regulated miRNAs, and the numbers represent the number ofup- and down-regulated miRNAs. Figure 3. Results of clustering analysis ofdifferential miRNAs. HN: excess N; LN: no N; Y: Yanshu4; D: Atlantic; S: seedling;A: bud onset. (A)LN_YLSvsLN_DLS (B)LN_YLAvsLN_DLA (C)HN_YLSvsHN_DLS. [file 12870_2022_3866_MOESM2_ESM.zip › Appendix B Figure 3-A.tiff]

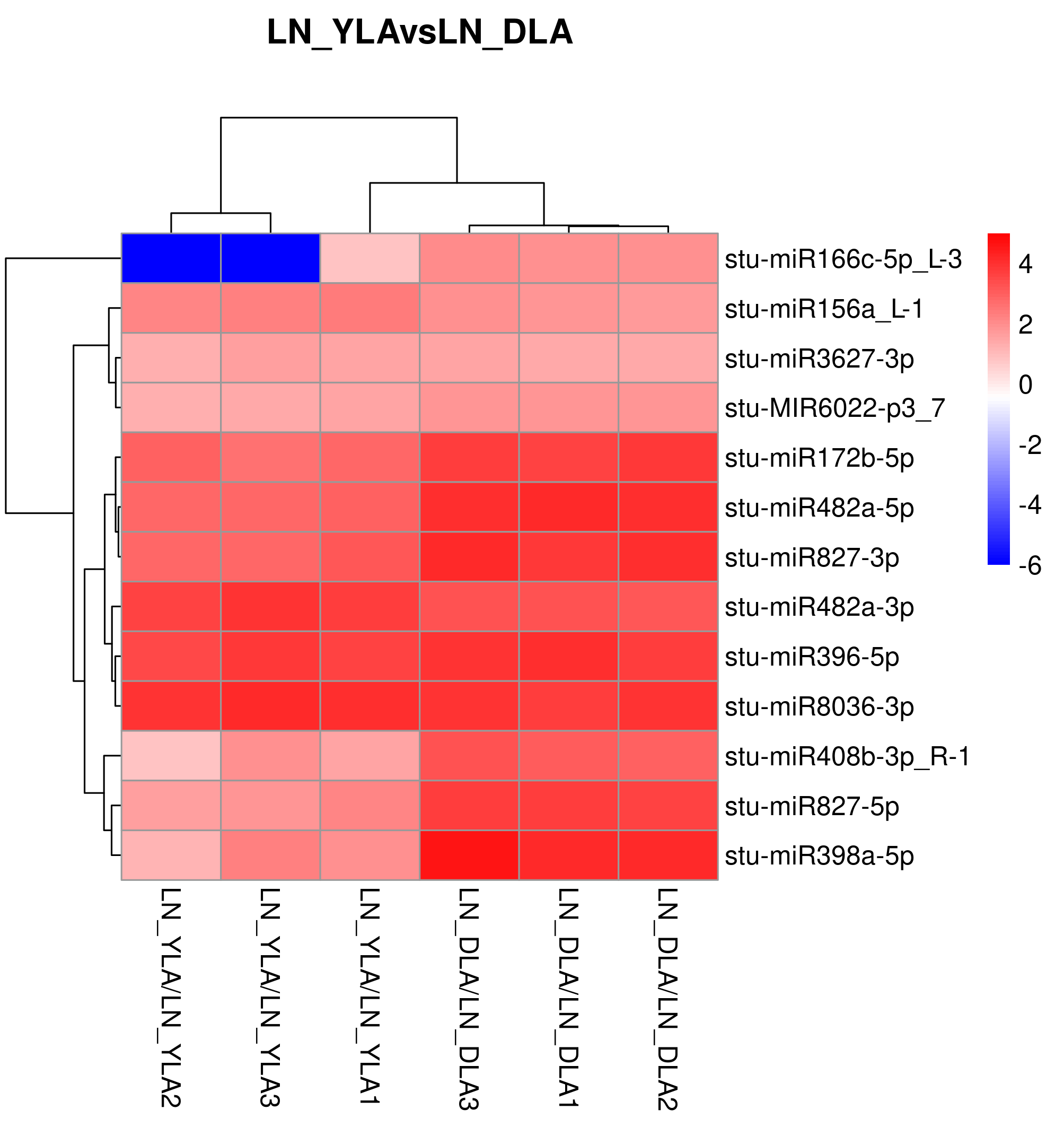

Supplement: Supplementary file 2 — Additional file 2: Figure 1. Analysis of variability among groups of differential miRNAsHN: excessive N application; LN: no N application; Y: Yanshu4; D: Atlantic;S: seedling stage; A: budding stage (A) LN_YLSvsLN_DLS (B) LN_YLAvsLN_DLA (C) HN_YLSvsHN_DLS. (D)HN_YLAvsHN_DLA (E) HN_DLSvsLN_DLS (F) HN_DLAvsLN_DLA. (G) HN_YLSvsLN_YLS (H)HN_YLAvsLN_YLA (I) LN_DLAvsLN_DLS. (J)HN_DLAvsHN_DLS (K)LN_YLAvsLN_YLS (L)HN_YLAvsHN_YLS.(M)LN_YRSvsLN_DRS (N)LN_YRAvsLN_DRA (O)HN_YRSvsHN_DRS. (P)HN_YRAvsHN_DRA (Q)HN_DRSvsLN_DRS(R)HN_DRAvsLN_DRA. (S)HN_YRSvsLN_YRS (T)HN_YRAvsLN_YRA (U)LN_DRAvsLN_DRS. (V)HN_DRAvsHN_DRS(W)LN_YRAvsLN_YRS (X)HN_YRAvsHN_YRS. Using the values of log2(fold change) as the horizontalcoordinate and -log10(p-value) as the vertical coordinate, volcanoplots were constructed for all miRNAs during differential expression analysis.The horizontal coordinate represents the fold change in the differentialexpression of miRNAs in different samples, and the vertical coordinaterepresents the statistical significance of the difference in the change inexpression levels of miRNAs. The red color represents up-regulatedsignificantly differentially expressed genes, the blue color representsdown-regulated significantly differentially expressed genes, and the grey dots representnon-significant differentially expressed genes. Figure 2. Comparison between groups of differential miRNAs. HN: excess N; LN:no N; Y: Yanshu4; D: Atlantic; S: seedling stage; A: budding stage. Thehorizontal coordinates indicate the data obtained after the comparison ofgroups, and the vertical coordinates indicate the number of up- anddown-regulated miRNAs. The red color represents up-regulated miRNAs, the bluecolor represents down-regulated miRNAs, and the numbers represent the number ofup- and down-regulated miRNAs. Figure 3. Results of clustering analysis ofdifferential miRNAs. HN: excess N; LN: no N; Y: Yanshu4; D: Atlantic; S: seedling;A: bud onset. (A)LN_YLSvsLN_DLS (B)LN_YLAvsLN_DLA (C)HN_YLSvsHN_DLS. [file 12870_2022_3866_MOESM2_ESM.zip › Appendix B Figure 3-B.tiff]

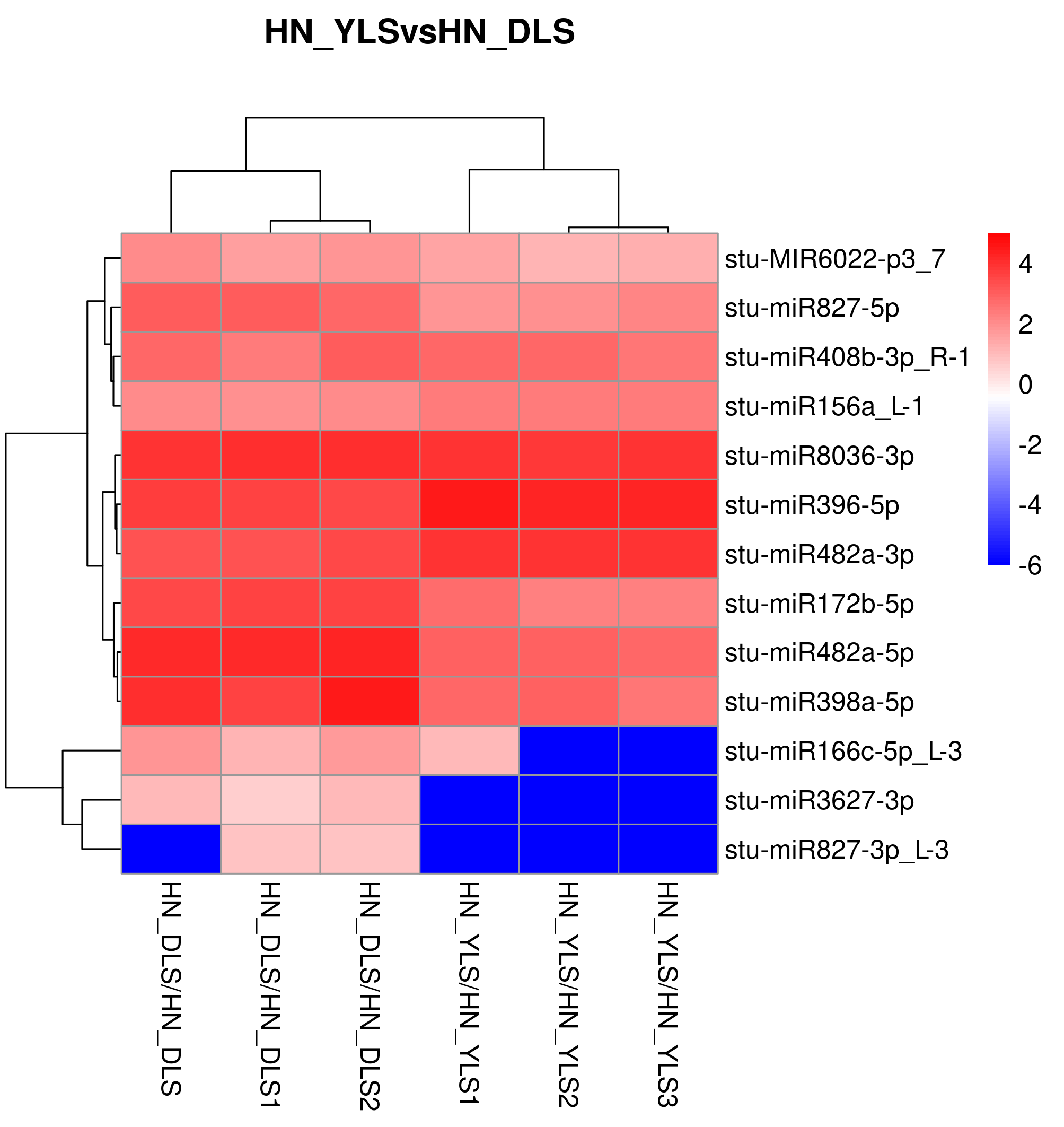

Supplement: Supplementary file 2 — Additional file 2: Figure 1. Analysis of variability among groups of differential miRNAsHN: excessive N application; LN: no N application; Y: Yanshu4; D: Atlantic;S: seedling stage; A: budding stage (A) LN_YLSvsLN_DLS (B) LN_YLAvsLN_DLA (C) HN_YLSvsHN_DLS. (D)HN_YLAvsHN_DLA (E) HN_DLSvsLN_DLS (F) HN_DLAvsLN_DLA. (G) HN_YLSvsLN_YLS (H)HN_YLAvsLN_YLA (I) LN_DLAvsLN_DLS. (J)HN_DLAvsHN_DLS (K)LN_YLAvsLN_YLS (L)HN_YLAvsHN_YLS.(M)LN_YRSvsLN_DRS (N)LN_YRAvsLN_DRA (O)HN_YRSvsHN_DRS. (P)HN_YRAvsHN_DRA (Q)HN_DRSvsLN_DRS(R)HN_DRAvsLN_DRA. (S)HN_YRSvsLN_YRS (T)HN_YRAvsLN_YRA (U)LN_DRAvsLN_DRS. (V)HN_DRAvsHN_DRS(W)LN_YRAvsLN_YRS (X)HN_YRAvsHN_YRS. Using the values of log2(fold change) as the horizontalcoordinate and -log10(p-value) as the vertical coordinate, volcanoplots were constructed for all miRNAs during differential expression analysis.The horizontal coordinate represents the fold change in the differentialexpression of miRNAs in different samples, and the vertical coordinaterepresents the statistical significance of the difference in the change inexpression levels of miRNAs. The red color represents up-regulatedsignificantly differentially expressed genes, the blue color representsdown-regulated significantly differentially expressed genes, and the grey dots representnon-significant differentially expressed genes. Figure 2. Comparison between groups of differential miRNAs. HN: excess N; LN:no N; Y: Yanshu4; D: Atlantic; S: seedling stage; A: budding stage. Thehorizontal coordinates indicate the data obtained after the comparison ofgroups, and the vertical coordinates indicate the number of up- anddown-regulated miRNAs. The red color represents up-regulated miRNAs, the bluecolor represents down-regulated miRNAs, and the numbers represent the number ofup- and down-regulated miRNAs. Figure 3. Results of clustering analysis ofdifferential miRNAs. HN: excess N; LN: no N; Y: Yanshu4; D: Atlantic; S: seedling;A: bud onset. (A)LN_YLSvsLN_DLS (B)LN_YLAvsLN_DLA (C)HN_YLSvsHN_DLS. [file 12870_2022_3866_MOESM2_ESM.zip › Appendix B Figure 3-C.tiff]

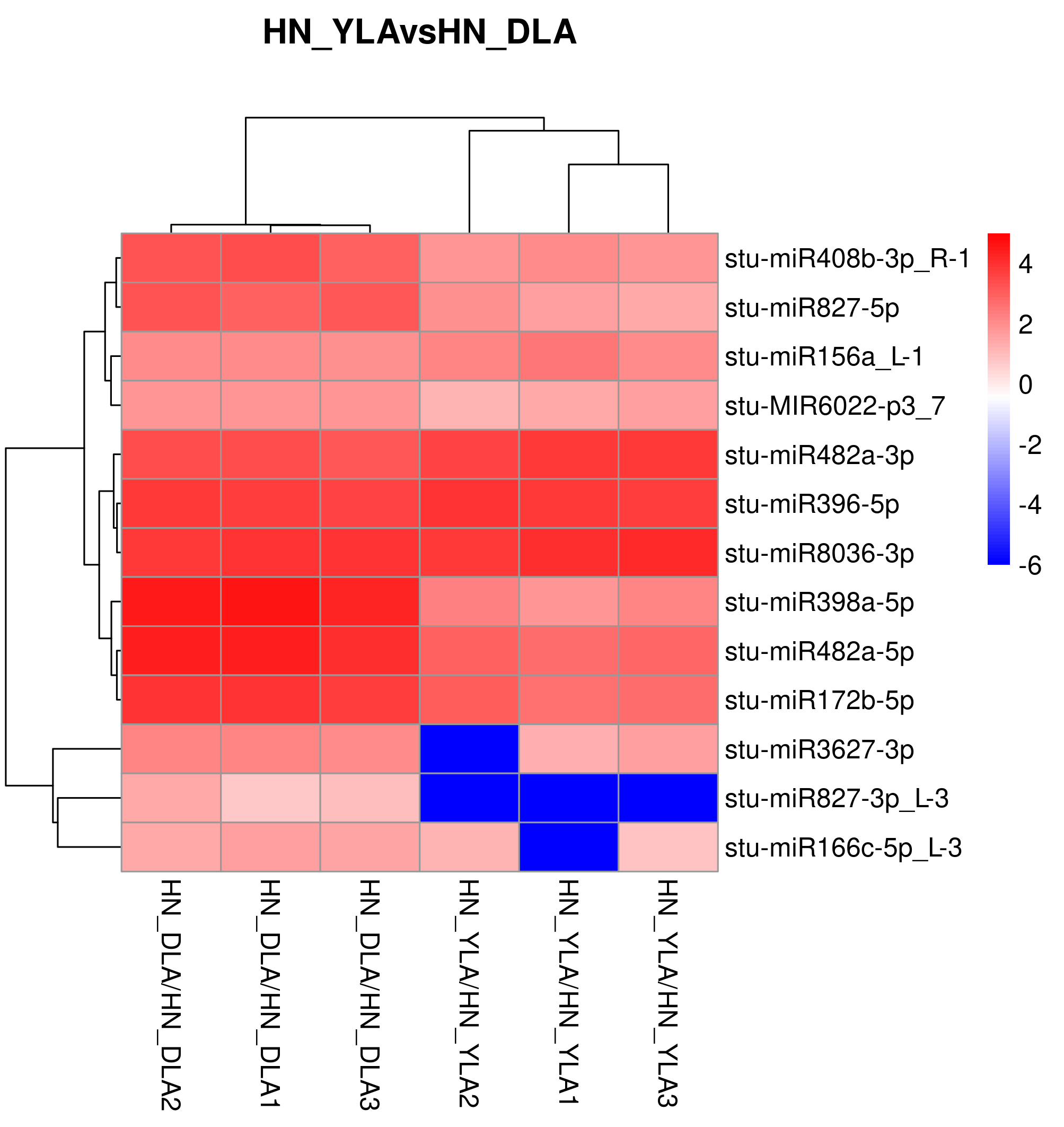

Supplement: Supplementary file 2 — Additional file 2: Figure 1. Analysis of variability among groups of differential miRNAsHN: excessive N application; LN: no N application; Y: Yanshu4; D: Atlantic;S: seedling stage; A: budding stage (A) LN_YLSvsLN_DLS (B) LN_YLAvsLN_DLA (C) HN_YLSvsHN_DLS. (D)HN_YLAvsHN_DLA (E) HN_DLSvsLN_DLS (F) HN_DLAvsLN_DLA. (G) HN_YLSvsLN_YLS (H)HN_YLAvsLN_YLA (I) LN_DLAvsLN_DLS. (J)HN_DLAvsHN_DLS (K)LN_YLAvsLN_YLS (L)HN_YLAvsHN_YLS.(M)LN_YRSvsLN_DRS (N)LN_YRAvsLN_DRA (O)HN_YRSvsHN_DRS. (P)HN_YRAvsHN_DRA (Q)HN_DRSvsLN_DRS(R)HN_DRAvsLN_DRA. (S)HN_YRSvsLN_YRS (T)HN_YRAvsLN_YRA (U)LN_DRAvsLN_DRS. (V)HN_DRAvsHN_DRS(W)LN_YRAvsLN_YRS (X)HN_YRAvsHN_YRS. Using the values of log2(fold change) as the horizontalcoordinate and -log10(p-value) as the vertical coordinate, volcanoplots were constructed for all miRNAs during differential expression analysis.The horizontal coordinate represents the fold change in the differentialexpression of miRNAs in different samples, and the vertical coordinaterepresents the statistical significance of the difference in the change inexpression levels of miRNAs. The red color represents up-regulatedsignificantly differentially expressed genes, the blue color representsdown-regulated significantly differentially expressed genes, and the grey dots representnon-significant differentially expressed genes. Figure 2. Comparison between groups of differential miRNAs. HN: excess N; LN:no N; Y: Yanshu4; D: Atlantic; S: seedling stage; A: budding stage. Thehorizontal coordinates indicate the data obtained after the comparison ofgroups, and the vertical coordinates indicate the number of up- anddown-regulated miRNAs. The red color represents up-regulated miRNAs, the bluecolor represents down-regulated miRNAs, and the numbers represent the number ofup- and down-regulated miRNAs. Figure 3. Results of clustering analysis ofdifferential miRNAs. HN: excess N; LN: no N; Y: Yanshu4; D: Atlantic; S: seedling;A: bud onset. (A)LN_YLSvsLN_DLS (B)LN_YLAvsLN_DLA (C)HN_YLSvsHN_DLS. [file 12870_2022_3866_MOESM2_ESM.zip › Appendix B Figure 3-D.tiff]

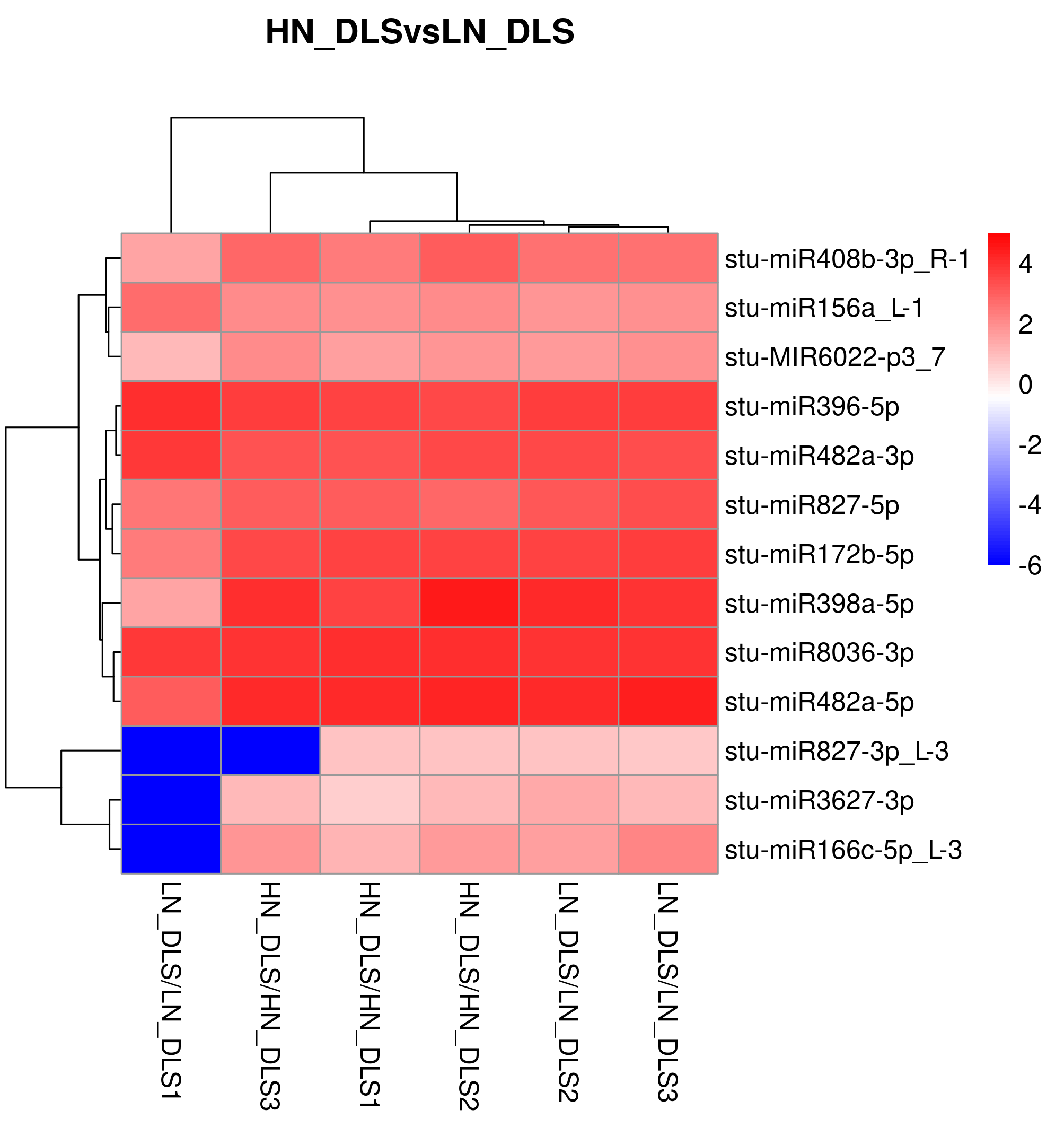

Supplement: Supplementary file 2 — Additional file 2: Figure 1. Analysis of variability among groups of differential miRNAsHN: excessive N application; LN: no N application; Y: Yanshu4; D: Atlantic;S: seedling stage; A: budding stage (A) LN_YLSvsLN_DLS (B) LN_YLAvsLN_DLA (C) HN_YLSvsHN_DLS. (D)HN_YLAvsHN_DLA (E) HN_DLSvsLN_DLS (F) HN_DLAvsLN_DLA. (G) HN_YLSvsLN_YLS (H)HN_YLAvsLN_YLA (I) LN_DLAvsLN_DLS. (J)HN_DLAvsHN_DLS (K)LN_YLAvsLN_YLS (L)HN_YLAvsHN_YLS.(M)LN_YRSvsLN_DRS (N)LN_YRAvsLN_DRA (O)HN_YRSvsHN_DRS. (P)HN_YRAvsHN_DRA (Q)HN_DRSvsLN_DRS(R)HN_DRAvsLN_DRA. (S)HN_YRSvsLN_YRS (T)HN_YRAvsLN_YRA (U)LN_DRAvsLN_DRS. (V)HN_DRAvsHN_DRS(W)LN_YRAvsLN_YRS (X)HN_YRAvsHN_YRS. Using the values of log2(fold change) as the horizontalcoordinate and -log10(p-value) as the vertical coordinate, volcanoplots were constructed for all miRNAs during differential expression analysis.The horizontal coordinate represents the fold change in the differentialexpression of miRNAs in different samples, and the vertical coordinaterepresents the statistical significance of the difference in the change inexpression levels of miRNAs. The red color represents up-regulatedsignificantly differentially expressed genes, the blue color representsdown-regulated significantly differentially expressed genes, and the grey dots representnon-significant differentially expressed genes. Figure 2. Comparison between groups of differential miRNAs. HN: excess N; LN:no N; Y: Yanshu4; D: Atlantic; S: seedling stage; A: budding stage. Thehorizontal coordinates indicate the data obtained after the comparison ofgroups, and the vertical coordinates indicate the number of up- anddown-regulated miRNAs. The red color represents up-regulated miRNAs, the bluecolor represents down-regulated miRNAs, and the numbers represent the number ofup- and down-regulated miRNAs. Figure 3. Results of clustering analysis ofdifferential miRNAs. HN: excess N; LN: no N; Y: Yanshu4; D: Atlantic; S: seedling;A: bud onset. (A)LN_YLSvsLN_DLS (B)LN_YLAvsLN_DLA (C)HN_YLSvsHN_DLS. [file 12870_2022_3866_MOESM2_ESM.zip › Appendix B Figure 3-E.tiff]

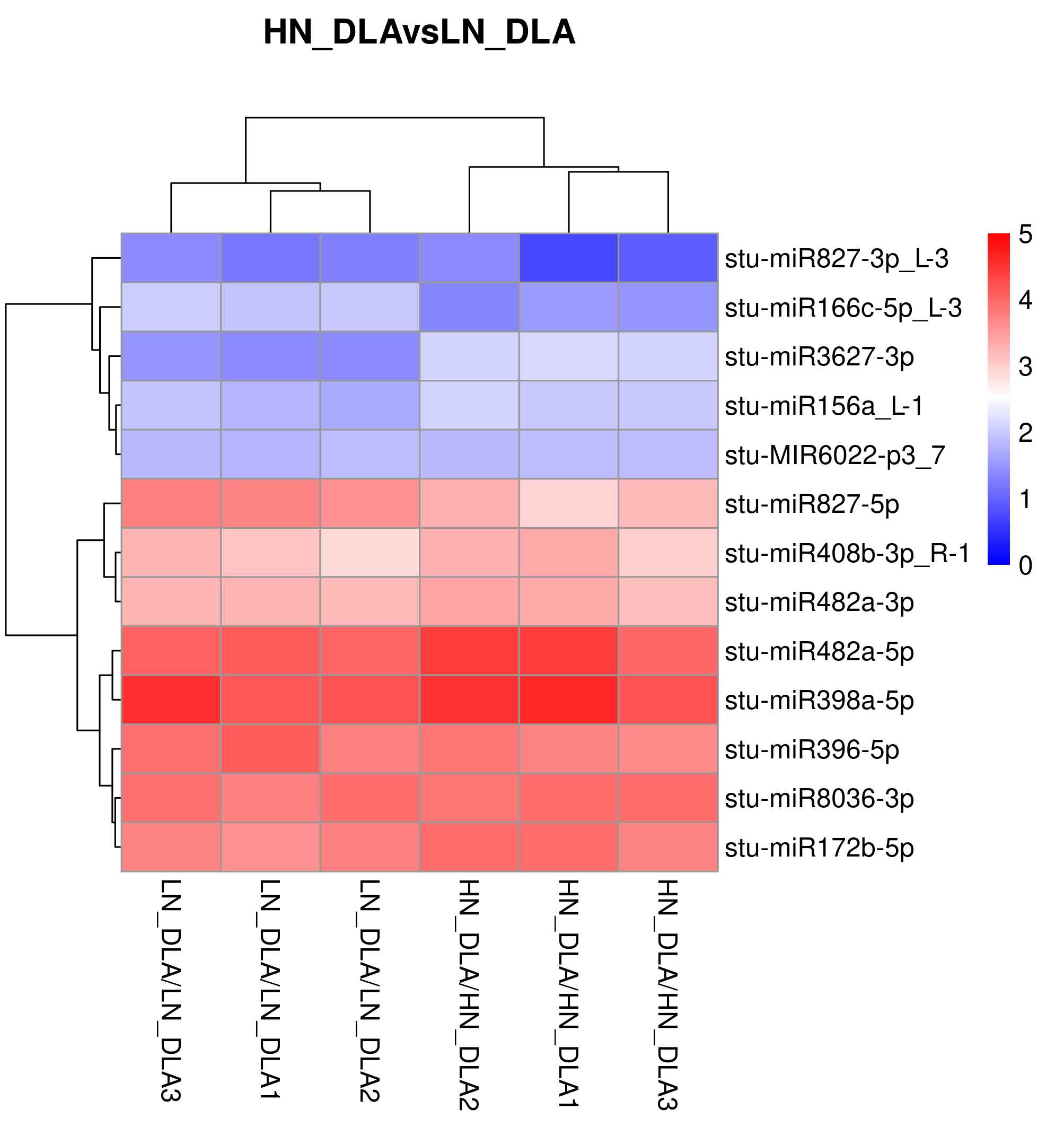

Supplement: Supplementary file 2 — Additional file 2: Figure 1. Analysis of variability among groups of differential miRNAsHN: excessive N application; LN: no N application; Y: Yanshu4; D: Atlantic;S: seedling stage; A: budding stage (A) LN_YLSvsLN_DLS (B) LN_YLAvsLN_DLA (C) HN_YLSvsHN_DLS. (D)HN_YLAvsHN_DLA (E) HN_DLSvsLN_DLS (F) HN_DLAvsLN_DLA. (G) HN_YLSvsLN_YLS (H)HN_YLAvsLN_YLA (I) LN_DLAvsLN_DLS. (J)HN_DLAvsHN_DLS (K)LN_YLAvsLN_YLS (L)HN_YLAvsHN_YLS.(M)LN_YRSvsLN_DRS (N)LN_YRAvsLN_DRA (O)HN_YRSvsHN_DRS. (P)HN_YRAvsHN_DRA (Q)HN_DRSvsLN_DRS(R)HN_DRAvsLN_DRA. (S)HN_YRSvsLN_YRS (T)HN_YRAvsLN_YRA (U)LN_DRAvsLN_DRS. (V)HN_DRAvsHN_DRS(W)LN_YRAvsLN_YRS (X)HN_YRAvsHN_YRS. Using the values of log2(fold change) as the horizontalcoordinate and -log10(p-value) as the vertical coordinate, volcanoplots were constructed for all miRNAs during differential expression analysis.The horizontal coordinate represents the fold change in the differentialexpression of miRNAs in different samples, and the vertical coordinaterepresents the statistical significance of the difference in the change inexpression levels of miRNAs. The red color represents up-regulatedsignificantly differentially expressed genes, the blue color representsdown-regulated significantly differentially expressed genes, and the grey dots representnon-significant differentially expressed genes. Figure 2. Comparison between groups of differential miRNAs. HN: excess N; LN:no N; Y: Yanshu4; D: Atlantic; S: seedling stage; A: budding stage. Thehorizontal coordinates indicate the data obtained after the comparison ofgroups, and the vertical coordinates indicate the number of up- anddown-regulated miRNAs. The red color represents up-regulated miRNAs, the bluecolor represents down-regulated miRNAs, and the numbers represent the number ofup- and down-regulated miRNAs. Figure 3. Results of clustering analysis ofdifferential miRNAs. HN: excess N; LN: no N; Y: Yanshu4; D: Atlantic; S: seedling;A: bud onset. (A)LN_YLSvsLN_DLS (B)LN_YLAvsLN_DLA (C)HN_YLSvsHN_DLS. [file 12870_2022_3866_MOESM2_ESM.zip › Appendix B Figure 3-F.tiff]

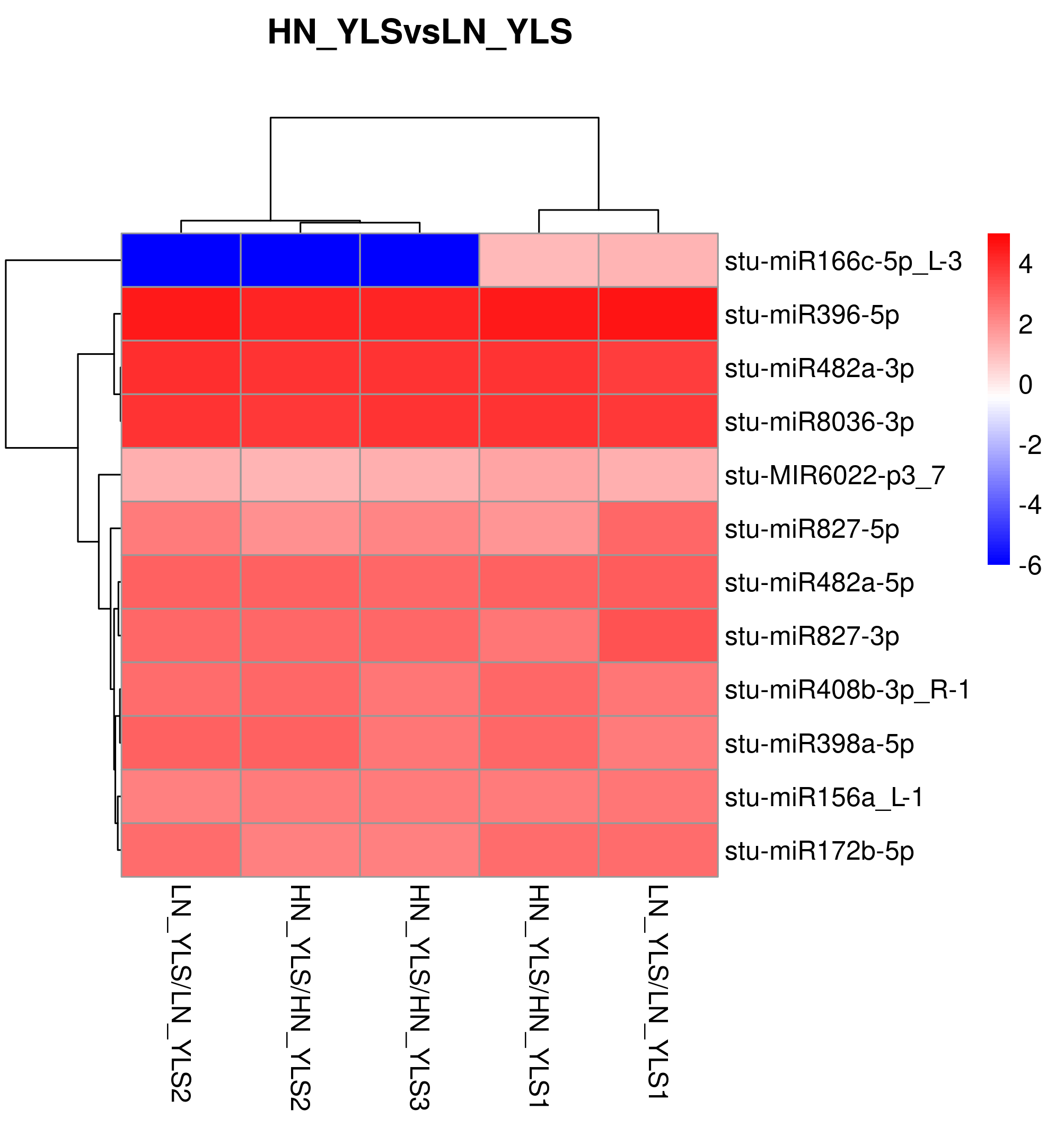

Supplement: Supplementary file 2 — Additional file 2: Figure 1. Analysis of variability among groups of differential miRNAsHN: excessive N application; LN: no N application; Y: Yanshu4; D: Atlantic;S: seedling stage; A: budding stage (A) LN_YLSvsLN_DLS (B) LN_YLAvsLN_DLA (C) HN_YLSvsHN_DLS. (D)HN_YLAvsHN_DLA (E) HN_DLSvsLN_DLS (F) HN_DLAvsLN_DLA. (G) HN_YLSvsLN_YLS (H)HN_YLAvsLN_YLA (I) LN_DLAvsLN_DLS. (J)HN_DLAvsHN_DLS (K)LN_YLAvsLN_YLS (L)HN_YLAvsHN_YLS.(M)LN_YRSvsLN_DRS (N)LN_YRAvsLN_DRA (O)HN_YRSvsHN_DRS. (P)HN_YRAvsHN_DRA (Q)HN_DRSvsLN_DRS(R)HN_DRAvsLN_DRA. (S)HN_YRSvsLN_YRS (T)HN_YRAvsLN_YRA (U)LN_DRAvsLN_DRS. (V)HN_DRAvsHN_DRS(W)LN_YRAvsLN_YRS (X)HN_YRAvsHN_YRS. Using the values of log2(fold change) as the horizontalcoordinate and -log10(p-value) as the vertical coordinate, volcanoplots were constructed for all miRNAs during differential expression analysis.The horizontal coordinate represents the fold change in the differentialexpression of miRNAs in different samples, and the vertical coordinaterepresents the statistical significance of the difference in the change inexpression levels of miRNAs. The red color represents up-regulatedsignificantly differentially expressed genes, the blue color representsdown-regulated significantly differentially expressed genes, and the grey dots representnon-significant differentially expressed genes. Figure 2. Comparison between groups of differential miRNAs. HN: excess N; LN:no N; Y: Yanshu4; D: Atlantic; S: seedling stage; A: budding stage. Thehorizontal coordinates indicate the data obtained after the comparison ofgroups, and the vertical coordinates indicate the number of up- anddown-regulated miRNAs. The red color represents up-regulated miRNAs, the bluecolor represents down-regulated miRNAs, and the numbers represent the number ofup- and down-regulated miRNAs. Figure 3. Results of clustering analysis ofdifferential miRNAs. HN: excess N; LN: no N; Y: Yanshu4; D: Atlantic; S: seedling;A: bud onset. (A)LN_YLSvsLN_DLS (B)LN_YLAvsLN_DLA (C)HN_YLSvsHN_DLS. [file 12870_2022_3866_MOESM2_ESM.zip › Appendix B Figure 3-G.tiff]

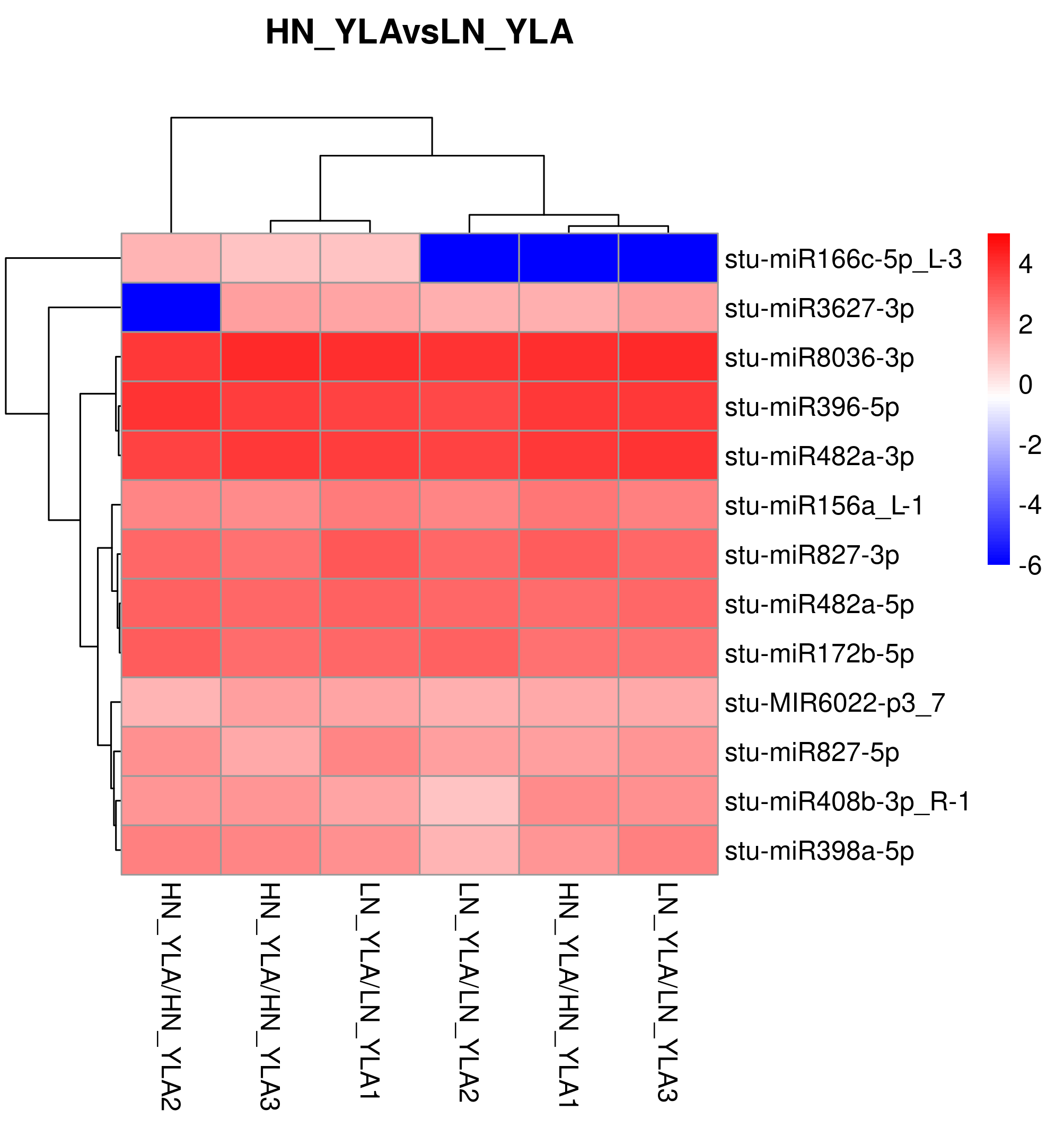

Supplement: Supplementary file 2 — Additional file 2: Figure 1. Analysis of variability among groups of differential miRNAsHN: excessive N application; LN: no N application; Y: Yanshu4; D: Atlantic;S: seedling stage; A: budding stage (A) LN_YLSvsLN_DLS (B) LN_YLAvsLN_DLA (C) HN_YLSvsHN_DLS. (D)HN_YLAvsHN_DLA (E) HN_DLSvsLN_DLS (F) HN_DLAvsLN_DLA. (G) HN_YLSvsLN_YLS (H)HN_YLAvsLN_YLA (I) LN_DLAvsLN_DLS. (J)HN_DLAvsHN_DLS (K)LN_YLAvsLN_YLS (L)HN_YLAvsHN_YLS.(M)LN_YRSvsLN_DRS (N)LN_YRAvsLN_DRA (O)HN_YRSvsHN_DRS. (P)HN_YRAvsHN_DRA (Q)HN_DRSvsLN_DRS(R)HN_DRAvsLN_DRA. (S)HN_YRSvsLN_YRS (T)HN_YRAvsLN_YRA (U)LN_DRAvsLN_DRS. (V)HN_DRAvsHN_DRS(W)LN_YRAvsLN_YRS (X)HN_YRAvsHN_YRS. Using the values of log2(fold change) as the horizontalcoordinate and -log10(p-value) as the vertical coordinate, volcanoplots were constructed for all miRNAs during differential expression analysis.The horizontal coordinate represents the fold change in the differentialexpression of miRNAs in different samples, and the vertical coordinaterepresents the statistical significance of the difference in the change inexpression levels of miRNAs. The red color represents up-regulatedsignificantly differentially expressed genes, the blue color representsdown-regulated significantly differentially expressed genes, and the grey dots representnon-significant differentially expressed genes. Figure 2. Comparison between groups of differential miRNAs. HN: excess N; LN:no N; Y: Yanshu4; D: Atlantic; S: seedling stage; A: budding stage. Thehorizontal coordinates indicate the data obtained after the comparison ofgroups, and the vertical coordinates indicate the number of up- anddown-regulated miRNAs. The red color represents up-regulated miRNAs, the bluecolor represents down-regulated miRNAs, and the numbers represent the number ofup- and down-regulated miRNAs. Figure 3. Results of clustering analysis ofdifferential miRNAs. HN: excess N; LN: no N; Y: Yanshu4; D: Atlantic; S: seedling;A: bud onset. (A)LN_YLSvsLN_DLS (B)LN_YLAvsLN_DLA (C)HN_YLSvsHN_DLS. [file 12870_2022_3866_MOESM2_ESM.zip › Appendix B Figure 3-H.tiff]

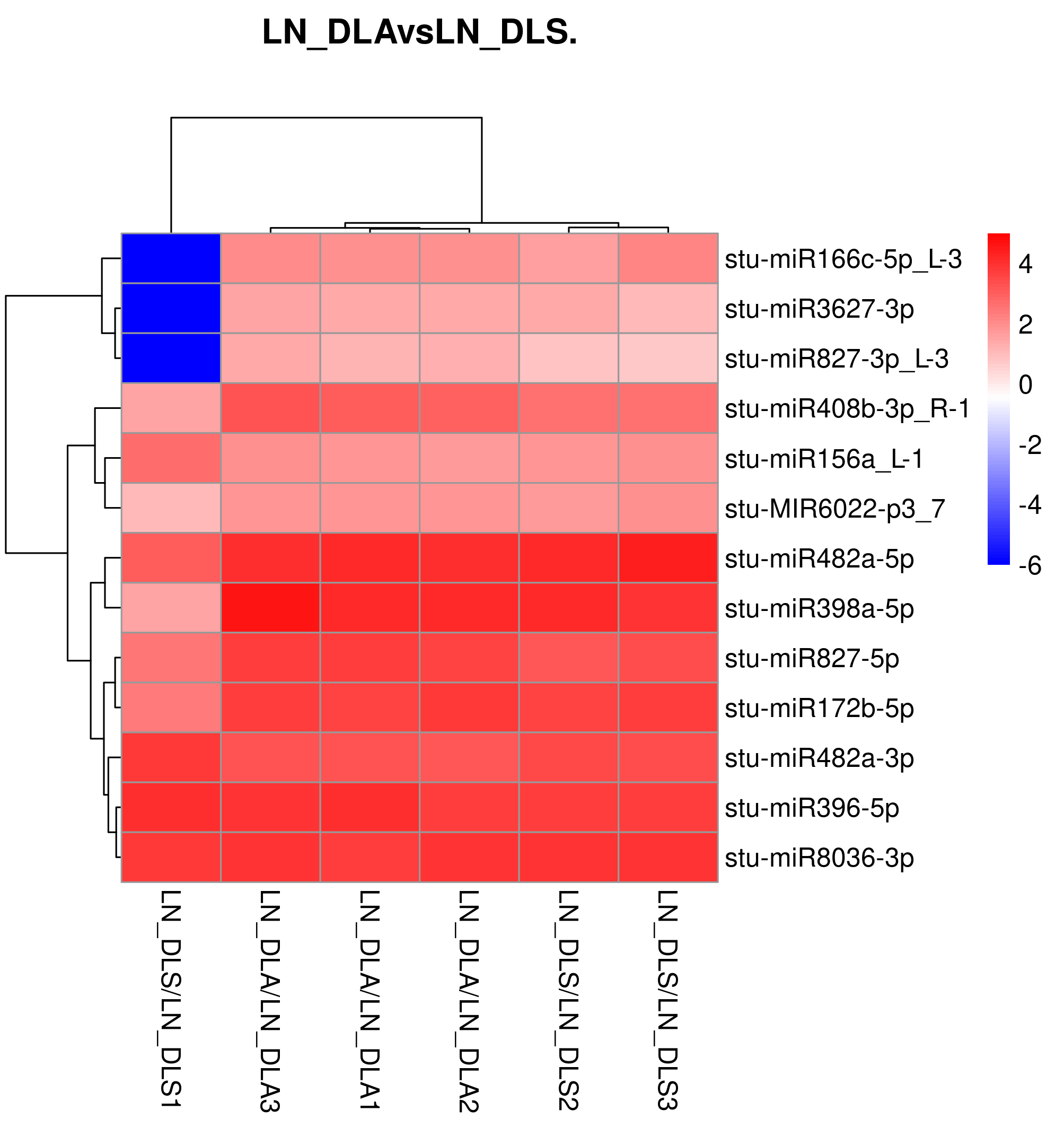

Supplement: Supplementary file 2 — Additional file 2: Figure 1. Analysis of variability among groups of differential miRNAsHN: excessive N application; LN: no N application; Y: Yanshu4; D: Atlantic;S: seedling stage; A: budding stage (A) LN_YLSvsLN_DLS (B) LN_YLAvsLN_DLA (C) HN_YLSvsHN_DLS. (D)HN_YLAvsHN_DLA (E) HN_DLSvsLN_DLS (F) HN_DLAvsLN_DLA. (G) HN_YLSvsLN_YLS (H)HN_YLAvsLN_YLA (I) LN_DLAvsLN_DLS. (J)HN_DLAvsHN_DLS (K)LN_YLAvsLN_YLS (L)HN_YLAvsHN_YLS.(M)LN_YRSvsLN_DRS (N)LN_YRAvsLN_DRA (O)HN_YRSvsHN_DRS. (P)HN_YRAvsHN_DRA (Q)HN_DRSvsLN_DRS(R)HN_DRAvsLN_DRA. (S)HN_YRSvsLN_YRS (T)HN_YRAvsLN_YRA (U)LN_DRAvsLN_DRS. (V)HN_DRAvsHN_DRS(W)LN_YRAvsLN_YRS (X)HN_YRAvsHN_YRS. Using the values of log2(fold change) as the horizontalcoordinate and -log10(p-value) as the vertical coordinate, volcanoplots were constructed for all miRNAs during differential expression analysis.The horizontal coordinate represents the fold change in the differentialexpression of miRNAs in different samples, and the vertical coordinaterepresents the statistical significance of the difference in the change inexpression levels of miRNAs. The red color represents up-regulatedsignificantly differentially expressed genes, the blue color representsdown-regulated significantly differentially expressed genes, and the grey dots representnon-significant differentially expressed genes. Figure 2. Comparison between groups of differential miRNAs. HN: excess N; LN:no N; Y: Yanshu4; D: Atlantic; S: seedling stage; A: budding stage. Thehorizontal coordinates indicate the data obtained after the comparison ofgroups, and the vertical coordinates indicate the number of up- anddown-regulated miRNAs. The red color represents up-regulated miRNAs, the bluecolor represents down-regulated miRNAs, and the numbers represent the number ofup- and down-regulated miRNAs. Figure 3. Results of clustering analysis ofdifferential miRNAs. HN: excess N; LN: no N; Y: Yanshu4; D: Atlantic; S: seedling;A: bud onset. (A)LN_YLSvsLN_DLS (B)LN_YLAvsLN_DLA (C)HN_YLSvsHN_DLS. [file 12870_2022_3866_MOESM2_ESM.zip › Appendix B Figure 3-I.tiff]

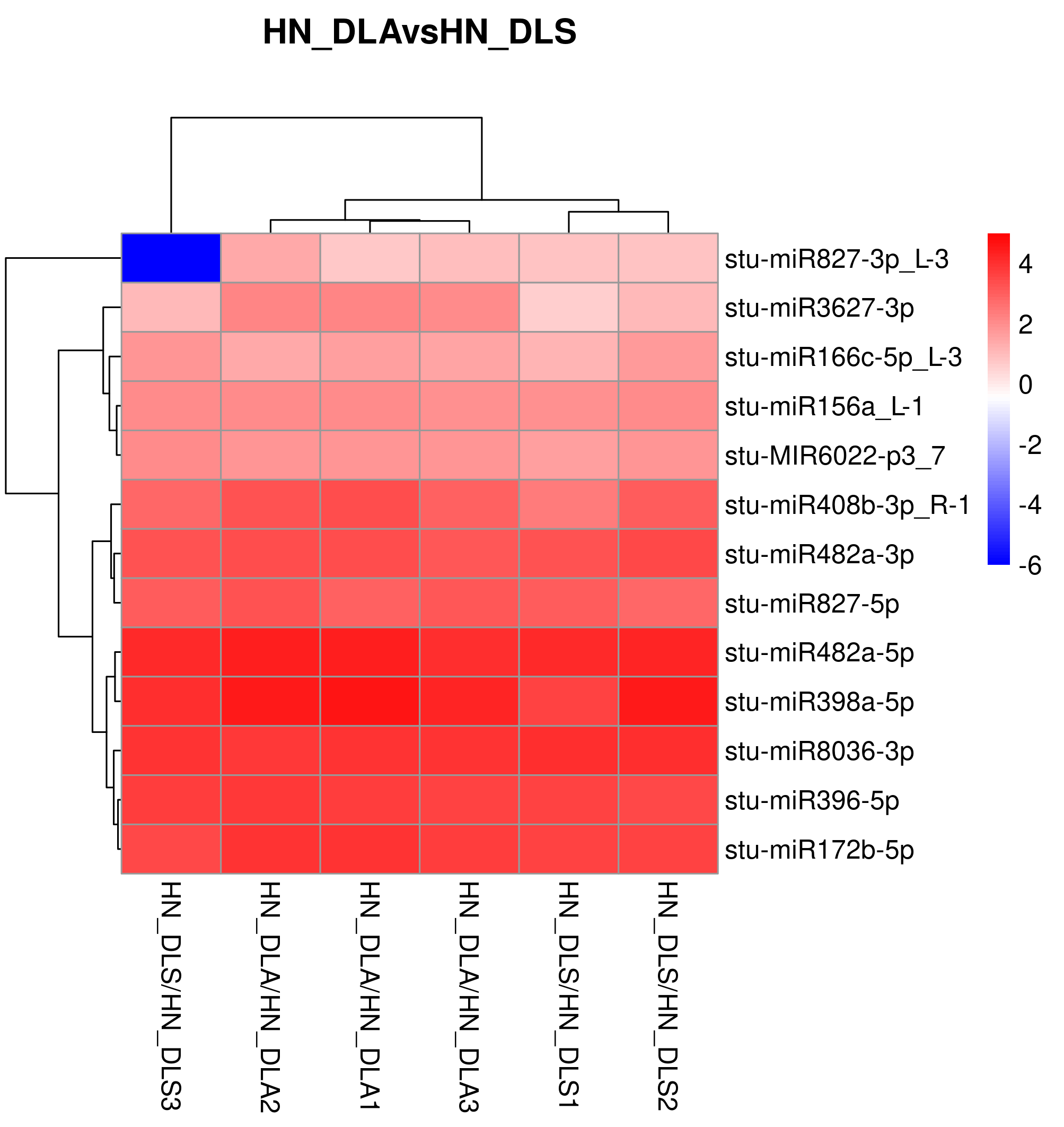

Supplement: Supplementary file 2 — Additional file 2: Figure 1. Analysis of variability among groups of differential miRNAsHN: excessive N application; LN: no N application; Y: Yanshu4; D: Atlantic;S: seedling stage; A: budding stage (A) LN_YLSvsLN_DLS (B) LN_YLAvsLN_DLA (C) HN_YLSvsHN_DLS. (D)HN_YLAvsHN_DLA (E) HN_DLSvsLN_DLS (F) HN_DLAvsLN_DLA. (G) HN_YLSvsLN_YLS (H)HN_YLAvsLN_YLA (I) LN_DLAvsLN_DLS. (J)HN_DLAvsHN_DLS (K)LN_YLAvsLN_YLS (L)HN_YLAvsHN_YLS.(M)LN_YRSvsLN_DRS (N)LN_YRAvsLN_DRA (O)HN_YRSvsHN_DRS. (P)HN_YRAvsHN_DRA (Q)HN_DRSvsLN_DRS(R)HN_DRAvsLN_DRA. (S)HN_YRSvsLN_YRS (T)HN_YRAvsLN_YRA (U)LN_DRAvsLN_DRS. (V)HN_DRAvsHN_DRS(W)LN_YRAvsLN_YRS (X)HN_YRAvsHN_YRS. Using the values of log2(fold change) as the horizontalcoordinate and -log10(p-value) as the vertical coordinate, volcanoplots were constructed for all miRNAs during differential expression analysis.The horizontal coordinate represents the fold change in the differentialexpression of miRNAs in different samples, and the vertical coordinaterepresents the statistical significance of the difference in the change inexpression levels of miRNAs. The red color represents up-regulatedsignificantly differentially expressed genes, the blue color representsdown-regulated significantly differentially expressed genes, and the grey dots representnon-significant differentially expressed genes. Figure 2. Comparison between groups of differential miRNAs. HN: excess N; LN:no N; Y: Yanshu4; D: Atlantic; S: seedling stage; A: budding stage. Thehorizontal coordinates indicate the data obtained after the comparison ofgroups, and the vertical coordinates indicate the number of up- anddown-regulated miRNAs. The red color represents up-regulated miRNAs, the bluecolor represents down-regulated miRNAs, and the numbers represent the number ofup- and down-regulated miRNAs. Figure 3. Results of clustering analysis ofdifferential miRNAs. HN: excess N; LN: no N; Y: Yanshu4; D: Atlantic; S: seedling;A: bud onset. (A)LN_YLSvsLN_DLS (B)LN_YLAvsLN_DLA (C)HN_YLSvsHN_DLS. [file 12870_2022_3866_MOESM2_ESM.zip › Appendix B Figure 3-J.tiff]

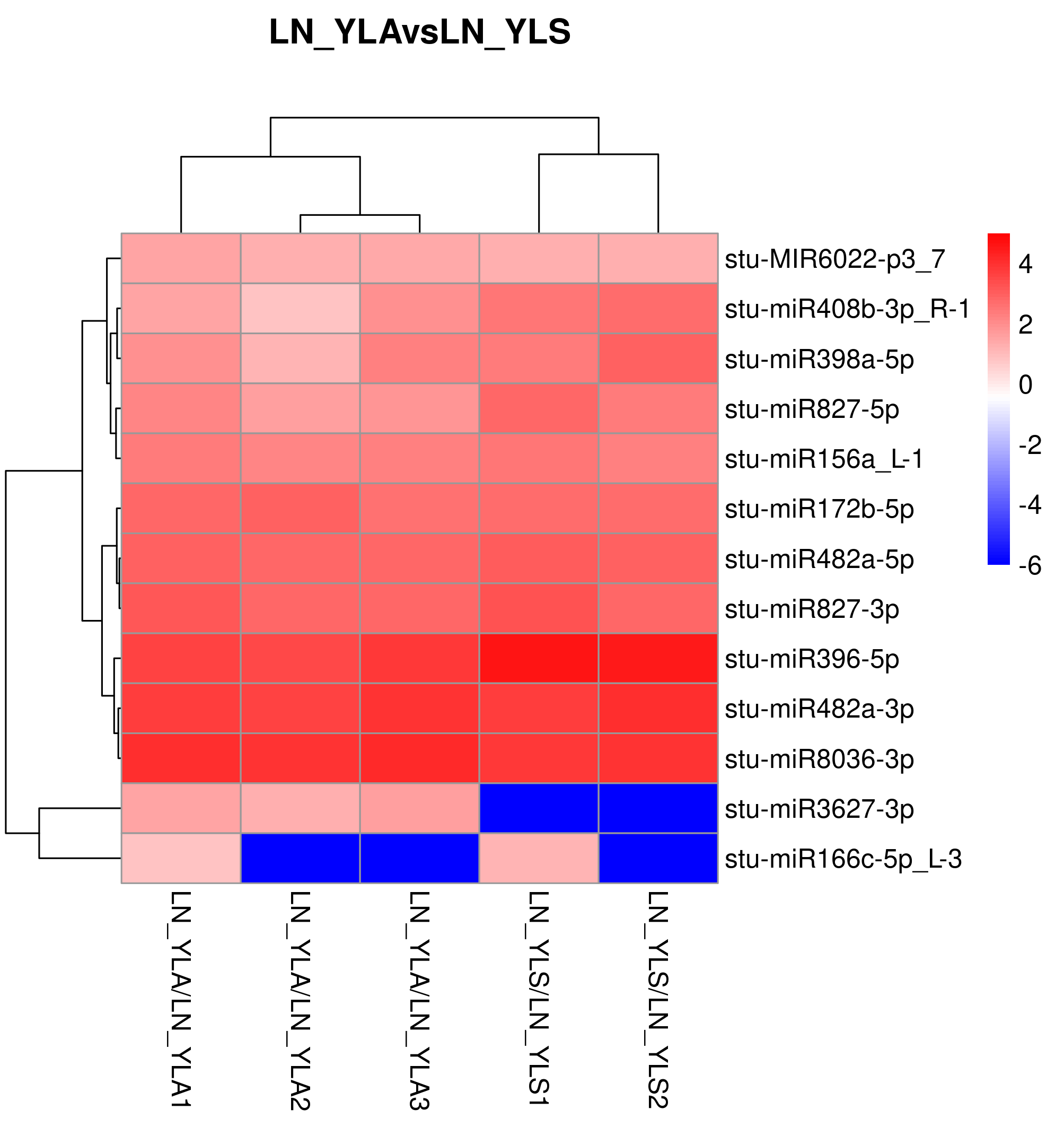

Supplement: Supplementary file 2 — Additional file 2: Figure 1. Analysis of variability among groups of differential miRNAsHN: excessive N application; LN: no N application; Y: Yanshu4; D: Atlantic;S: seedling stage; A: budding stage (A) LN_YLSvsLN_DLS (B) LN_YLAvsLN_DLA (C) HN_YLSvsHN_DLS. (D)HN_YLAvsHN_DLA (E) HN_DLSvsLN_DLS (F) HN_DLAvsLN_DLA. (G) HN_YLSvsLN_YLS (H)HN_YLAvsLN_YLA (I) LN_DLAvsLN_DLS. (J)HN_DLAvsHN_DLS (K)LN_YLAvsLN_YLS (L)HN_YLAvsHN_YLS.(M)LN_YRSvsLN_DRS (N)LN_YRAvsLN_DRA (O)HN_YRSvsHN_DRS. (P)HN_YRAvsHN_DRA (Q)HN_DRSvsLN_DRS(R)HN_DRAvsLN_DRA. (S)HN_YRSvsLN_YRS (T)HN_YRAvsLN_YRA (U)LN_DRAvsLN_DRS. (V)HN_DRAvsHN_DRS(W)LN_YRAvsLN_YRS (X)HN_YRAvsHN_YRS. Using the values of log2(fold change) as the horizontalcoordinate and -log10(p-value) as the vertical coordinate, volcanoplots were constructed for all miRNAs during differential expression analysis.The horizontal coordinate represents the fold change in the differentialexpression of miRNAs in different samples, and the vertical coordinaterepresents the statistical significance of the difference in the change inexpression levels of miRNAs. The red color represents up-regulatedsignificantly differentially expressed genes, the blue color representsdown-regulated significantly differentially expressed genes, and the grey dots representnon-significant differentially expressed genes. Figure 2. Comparison between groups of differential miRNAs. HN: excess N; LN:no N; Y: Yanshu4; D: Atlantic; S: seedling stage; A: budding stage. Thehorizontal coordinates indicate the data obtained after the comparison ofgroups, and the vertical coordinates indicate the number of up- anddown-regulated miRNAs. The red color represents up-regulated miRNAs, the bluecolor represents down-regulated miRNAs, and the numbers represent the number ofup- and down-regulated miRNAs. Figure 3. Results of clustering analysis ofdifferential miRNAs. HN: excess N; LN: no N; Y: Yanshu4; D: Atlantic; S: seedling;A: bud onset. (A)LN_YLSvsLN_DLS (B)LN_YLAvsLN_DLA (C)HN_YLSvsHN_DLS. [file 12870_2022_3866_MOESM2_ESM.zip › Appendix B Figure 3-K.tiff]

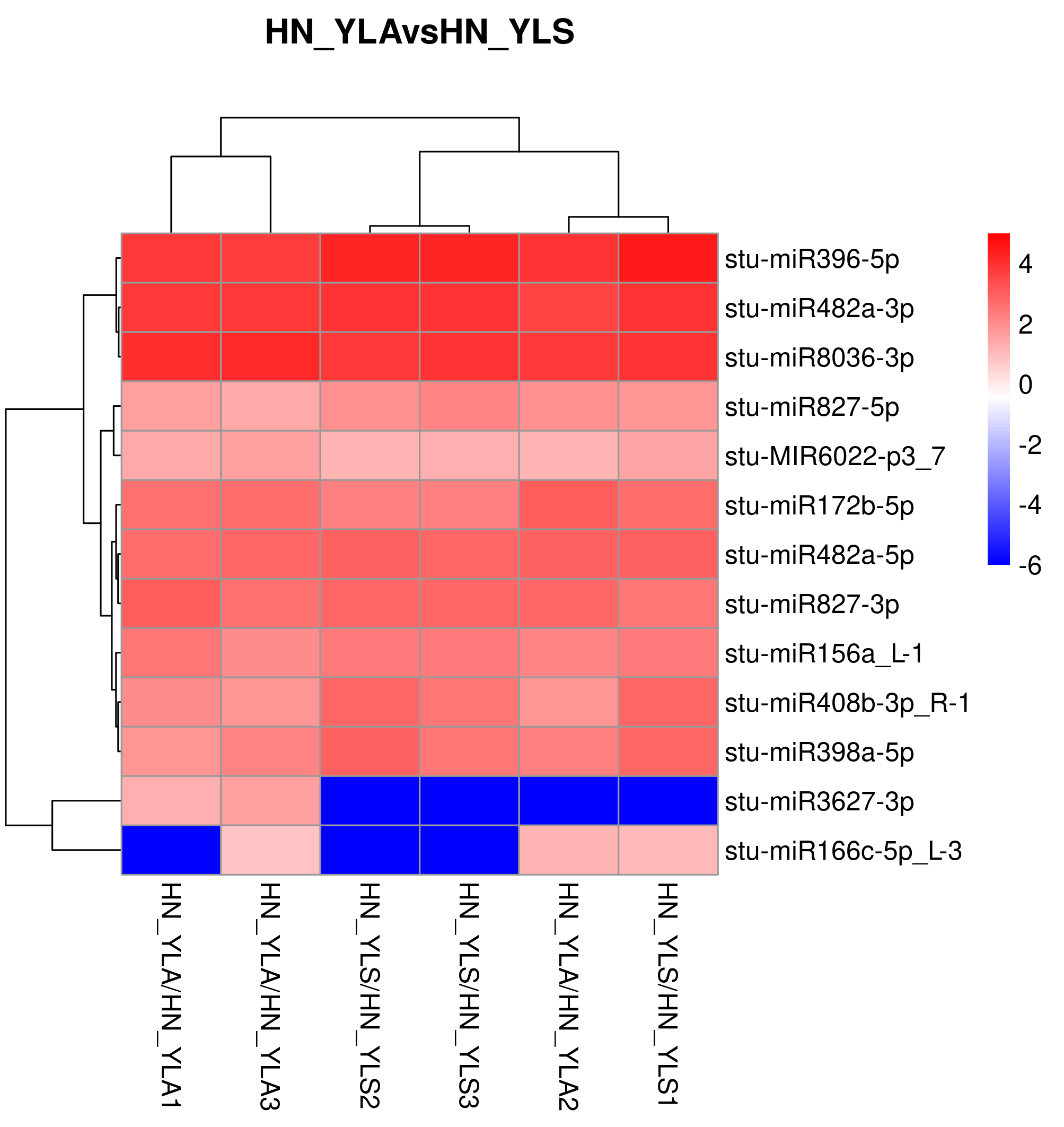

Supplement: Supplementary file 2 — Additional file 2: Figure 1. Analysis of variability among groups of differential miRNAsHN: excessive N application; LN: no N application; Y: Yanshu4; D: Atlantic;S: seedling stage; A: budding stage (A) LN_YLSvsLN_DLS (B) LN_YLAvsLN_DLA (C) HN_YLSvsHN_DLS. (D)HN_YLAvsHN_DLA (E) HN_DLSvsLN_DLS (F) HN_DLAvsLN_DLA. (G) HN_YLSvsLN_YLS (H)HN_YLAvsLN_YLA (I) LN_DLAvsLN_DLS. (J)HN_DLAvsHN_DLS (K)LN_YLAvsLN_YLS (L)HN_YLAvsHN_YLS.(M)LN_YRSvsLN_DRS (N)LN_YRAvsLN_DRA (O)HN_YRSvsHN_DRS. (P)HN_YRAvsHN_DRA (Q)HN_DRSvsLN_DRS(R)HN_DRAvsLN_DRA. (S)HN_YRSvsLN_YRS (T)HN_YRAvsLN_YRA (U)LN_DRAvsLN_DRS. (V)HN_DRAvsHN_DRS(W)LN_YRAvsLN_YRS (X)HN_YRAvsHN_YRS. Using the values of log2(fold change) as the horizontalcoordinate and -log10(p-value) as the vertical coordinate, volcanoplots were constructed for all miRNAs during differential expression analysis.The horizontal coordinate represents the fold change in the differentialexpression of miRNAs in different samples, and the vertical coordinaterepresents the statistical significance of the difference in the change inexpression levels of miRNAs. The red color represents up-regulatedsignificantly differentially expressed genes, the blue color representsdown-regulated significantly differentially expressed genes, and the grey dots representnon-significant differentially expressed genes. Figure 2. Comparison between groups of differential miRNAs. HN: excess N; LN:no N; Y: Yanshu4; D: Atlantic; S: seedling stage; A: budding stage. Thehorizontal coordinates indicate the data obtained after the comparison ofgroups, and the vertical coordinates indicate the number of up- anddown-regulated miRNAs. The red color represents up-regulated miRNAs, the bluecolor represents down-regulated miRNAs, and the numbers represent the number ofup- and down-regulated miRNAs. Figure 3. Results of clustering analysis ofdifferential miRNAs. HN: excess N; LN: no N; Y: Yanshu4; D: Atlantic; S: seedling;A: bud onset. (A)LN_YLSvsLN_DLS (B)LN_YLAvsLN_DLA (C)HN_YLSvsHN_DLS. [file 12870_2022_3866_MOESM2_ESM.zip › Appendix B Figure 3-L.tiff]

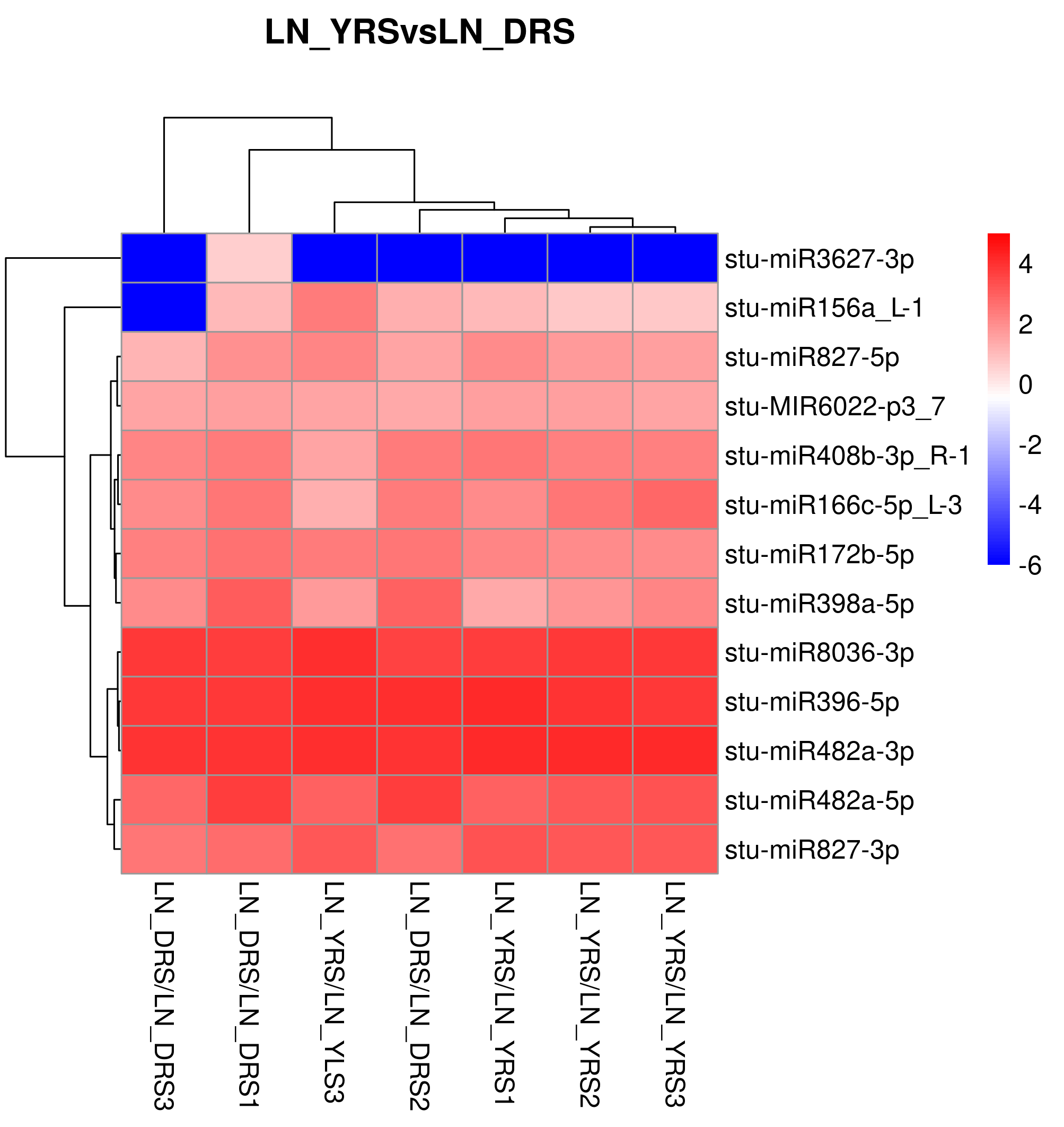

Supplement: Supplementary file 2 — Additional file 2: Figure 1. Analysis of variability among groups of differential miRNAsHN: excessive N application; LN: no N application; Y: Yanshu4; D: Atlantic;S: seedling stage; A: budding stage (A) LN_YLSvsLN_DLS (B) LN_YLAvsLN_DLA (C) HN_YLSvsHN_DLS. (D)HN_YLAvsHN_DLA (E) HN_DLSvsLN_DLS (F) HN_DLAvsLN_DLA. (G) HN_YLSvsLN_YLS (H)HN_YLAvsLN_YLA (I) LN_DLAvsLN_DLS. (J)HN_DLAvsHN_DLS (K)LN_YLAvsLN_YLS (L)HN_YLAvsHN_YLS.(M)LN_YRSvsLN_DRS (N)LN_YRAvsLN_DRA (O)HN_YRSvsHN_DRS. (P)HN_YRAvsHN_DRA (Q)HN_DRSvsLN_DRS(R)HN_DRAvsLN_DRA. (S)HN_YRSvsLN_YRS (T)HN_YRAvsLN_YRA (U)LN_DRAvsLN_DRS. (V)HN_DRAvsHN_DRS(W)LN_YRAvsLN_YRS (X)HN_YRAvsHN_YRS. Using the values of log2(fold change) as the horizontalcoordinate and -log10(p-value) as the vertical coordinate, volcanoplots were constructed for all miRNAs during differential expression analysis.The horizontal coordinate represents the fold change in the differentialexpression of miRNAs in different samples, and the vertical coordinaterepresents the statistical significance of the difference in the change inexpression levels of miRNAs. The red color represents up-regulatedsignificantly differentially expressed genes, the blue color representsdown-regulated significantly differentially expressed genes, and the grey dots representnon-significant differentially expressed genes. Figure 2. Comparison between groups of differential miRNAs. HN: excess N; LN:no N; Y: Yanshu4; D: Atlantic; S: seedling stage; A: budding stage. Thehorizontal coordinates indicate the data obtained after the comparison ofgroups, and the vertical coordinates indicate the number of up- anddown-regulated miRNAs. The red color represents up-regulated miRNAs, the bluecolor represents down-regulated miRNAs, and the numbers represent the number ofup- and down-regulated miRNAs. Figure 3. Results of clustering analysis ofdifferential miRNAs. HN: excess N; LN: no N; Y: Yanshu4; D: Atlantic; S: seedling;A: bud onset. (A)LN_YLSvsLN_DLS (B)LN_YLAvsLN_DLA (C)HN_YLSvsHN_DLS. [file 12870_2022_3866_MOESM2_ESM.zip › Appendix B Figure 3-M.tiff]

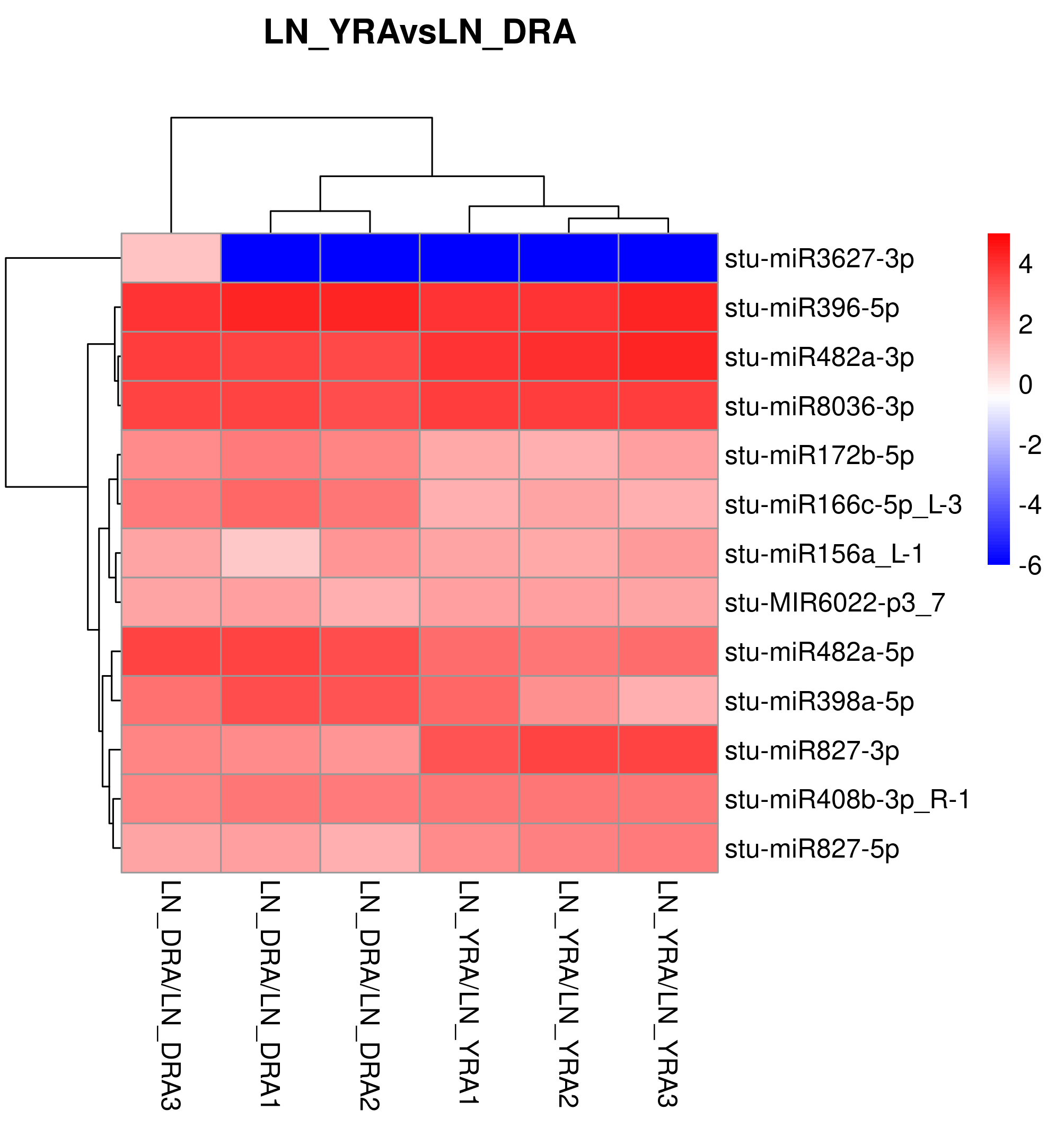

Supplement: Supplementary file 2 — Additional file 2: Figure 1. Analysis of variability among groups of differential miRNAsHN: excessive N application; LN: no N application; Y: Yanshu4; D: Atlantic;S: seedling stage; A: budding stage (A) LN_YLSvsLN_DLS (B) LN_YLAvsLN_DLA (C) HN_YLSvsHN_DLS. (D)HN_YLAvsHN_DLA (E) HN_DLSvsLN_DLS (F) HN_DLAvsLN_DLA. (G) HN_YLSvsLN_YLS (H)HN_YLAvsLN_YLA (I) LN_DLAvsLN_DLS. (J)HN_DLAvsHN_DLS (K)LN_YLAvsLN_YLS (L)HN_YLAvsHN_YLS.(M)LN_YRSvsLN_DRS (N)LN_YRAvsLN_DRA (O)HN_YRSvsHN_DRS. (P)HN_YRAvsHN_DRA (Q)HN_DRSvsLN_DRS(R)HN_DRAvsLN_DRA. (S)HN_YRSvsLN_YRS (T)HN_YRAvsLN_YRA (U)LN_DRAvsLN_DRS. (V)HN_DRAvsHN_DRS(W)LN_YRAvsLN_YRS (X)HN_YRAvsHN_YRS. Using the values of log2(fold change) as the horizontalcoordinate and -log10(p-value) as the vertical coordinate, volcanoplots were constructed for all miRNAs during differential expression analysis.The horizontal coordinate represents the fold change in the differentialexpression of miRNAs in different samples, and the vertical coordinaterepresents the statistical significance of the difference in the change inexpression levels of miRNAs. The red color represents up-regulatedsignificantly differentially expressed genes, the blue color representsdown-regulated significantly differentially expressed genes, and the grey dots representnon-significant differentially expressed genes. Figure 2. Comparison between groups of differential miRNAs. HN: excess N; LN:no N; Y: Yanshu4; D: Atlantic; S: seedling stage; A: budding stage. Thehorizontal coordinates indicate the data obtained after the comparison ofgroups, and the vertical coordinates indicate the number of up- anddown-regulated miRNAs. The red color represents up-regulated miRNAs, the bluecolor represents down-regulated miRNAs, and the numbers represent the number ofup- and down-regulated miRNAs. Figure 3. Results of clustering analysis ofdifferential miRNAs. HN: excess N; LN: no N; Y: Yanshu4; D: Atlantic; S: seedling;A: bud onset. (A)LN_YLSvsLN_DLS (B)LN_YLAvsLN_DLA (C)HN_YLSvsHN_DLS. [file 12870_2022_3866_MOESM2_ESM.zip › Appendix B Figure 3-N.tiff]

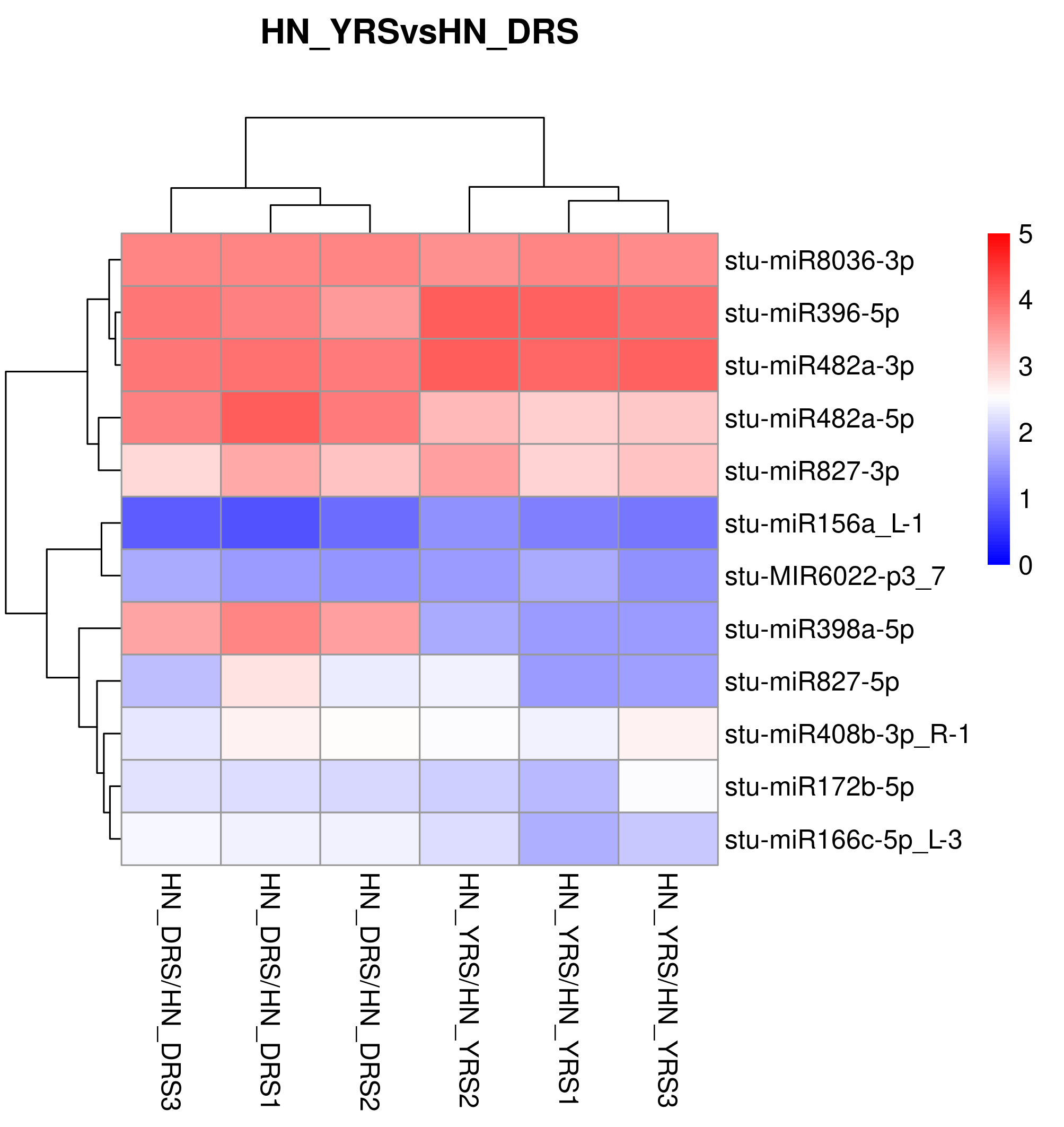

Supplement: Supplementary file 2 — Additional file 2: Figure 1. Analysis of variability among groups of differential miRNAsHN: excessive N application; LN: no N application; Y: Yanshu4; D: Atlantic;S: seedling stage; A: budding stage (A) LN_YLSvsLN_DLS (B) LN_YLAvsLN_DLA (C) HN_YLSvsHN_DLS. (D)HN_YLAvsHN_DLA (E) HN_DLSvsLN_DLS (F) HN_DLAvsLN_DLA. (G) HN_YLSvsLN_YLS (H)HN_YLAvsLN_YLA (I) LN_DLAvsLN_DLS. (J)HN_DLAvsHN_DLS (K)LN_YLAvsLN_YLS (L)HN_YLAvsHN_YLS.(M)LN_YRSvsLN_DRS (N)LN_YRAvsLN_DRA (O)HN_YRSvsHN_DRS. (P)HN_YRAvsHN_DRA (Q)HN_DRSvsLN_DRS(R)HN_DRAvsLN_DRA. (S)HN_YRSvsLN_YRS (T)HN_YRAvsLN_YRA (U)LN_DRAvsLN_DRS. (V)HN_DRAvsHN_DRS(W)LN_YRAvsLN_YRS (X)HN_YRAvsHN_YRS. Using the values of log2(fold change) as the horizontalcoordinate and -log10(p-value) as the vertical coordinate, volcanoplots were constructed for all miRNAs during differential expression analysis.The horizontal coordinate represents the fold change in the differentialexpression of miRNAs in different samples, and the vertical coordinaterepresents the statistical significance of the difference in the change inexpression levels of miRNAs. The red color represents up-regulatedsignificantly differentially expressed genes, the blue color representsdown-regulated significantly differentially expressed genes, and the grey dots representnon-significant differentially expressed genes. Figure 2. Comparison between groups of differential miRNAs. HN: excess N; LN:no N; Y: Yanshu4; D: Atlantic; S: seedling stage; A: budding stage. Thehorizontal coordinates indicate the data obtained after the comparison ofgroups, and the vertical coordinates indicate the number of up- anddown-regulated miRNAs. The red color represents up-regulated miRNAs, the bluecolor represents down-regulated miRNAs, and the numbers represent the number ofup- and down-regulated miRNAs. Figure 3. Results of clustering analysis ofdifferential miRNAs. HN: excess N; LN: no N; Y: Yanshu4; D: Atlantic; S: seedling;A: bud onset. (A)LN_YLSvsLN_DLS (B)LN_YLAvsLN_DLA (C)HN_YLSvsHN_DLS. [file 12870_2022_3866_MOESM2_ESM.zip › Appendix B Figure 3-O.tiff]

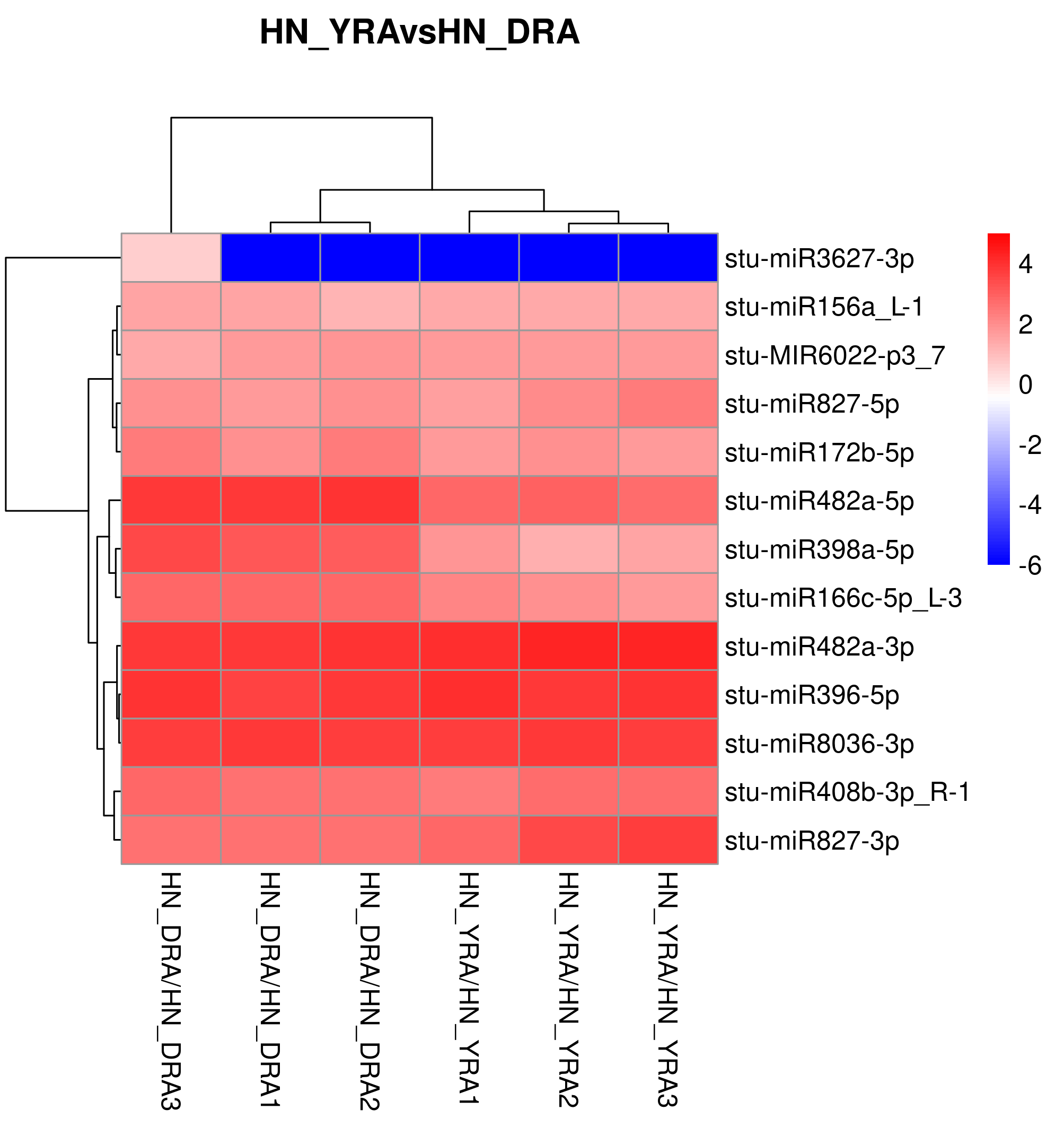

Supplement: Supplementary file 2 — Additional file 2: Figure 1. Analysis of variability among groups of differential miRNAsHN: excessive N application; LN: no N application; Y: Yanshu4; D: Atlantic;S: seedling stage; A: budding stage (A) LN_YLSvsLN_DLS (B) LN_YLAvsLN_DLA (C) HN_YLSvsHN_DLS. (D)HN_YLAvsHN_DLA (E) HN_DLSvsLN_DLS (F) HN_DLAvsLN_DLA. (G) HN_YLSvsLN_YLS (H)HN_YLAvsLN_YLA (I) LN_DLAvsLN_DLS. (J)HN_DLAvsHN_DLS (K)LN_YLAvsLN_YLS (L)HN_YLAvsHN_YLS.(M)LN_YRSvsLN_DRS (N)LN_YRAvsLN_DRA (O)HN_YRSvsHN_DRS. (P)HN_YRAvsHN_DRA (Q)HN_DRSvsLN_DRS(R)HN_DRAvsLN_DRA. (S)HN_YRSvsLN_YRS (T)HN_YRAvsLN_YRA (U)LN_DRAvsLN_DRS. (V)HN_DRAvsHN_DRS(W)LN_YRAvsLN_YRS (X)HN_YRAvsHN_YRS. Using the values of log2(fold change) as the horizontalcoordinate and -log10(p-value) as the vertical coordinate, volcanoplots were constructed for all miRNAs during differential expression analysis.The horizontal coordinate represents the fold change in the differentialexpression of miRNAs in different samples, and the vertical coordinaterepresents the statistical significance of the difference in the change inexpression levels of miRNAs. The red color represents up-regulatedsignificantly differentially expressed genes, the blue color representsdown-regulated significantly differentially expressed genes, and the grey dots representnon-significant differentially expressed genes. Figure 2. Comparison between groups of differential miRNAs. HN: excess N; LN:no N; Y: Yanshu4; D: Atlantic; S: seedling stage; A: budding stage. Thehorizontal coordinates indicate the data obtained after the comparison ofgroups, and the vertical coordinates indicate the number of up- anddown-regulated miRNAs. The red color represents up-regulated miRNAs, the bluecolor represents down-regulated miRNAs, and the numbers represent the number ofup- and down-regulated miRNAs. Figure 3. Results of clustering analysis ofdifferential miRNAs. HN: excess N; LN: no N; Y: Yanshu4; D: Atlantic; S: seedling;A: bud onset. (A)LN_YLSvsLN_DLS (B)LN_YLAvsLN_DLA (C)HN_YLSvsHN_DLS. [file 12870_2022_3866_MOESM2_ESM.zip › Appendix B Figure 3-P.tiff]

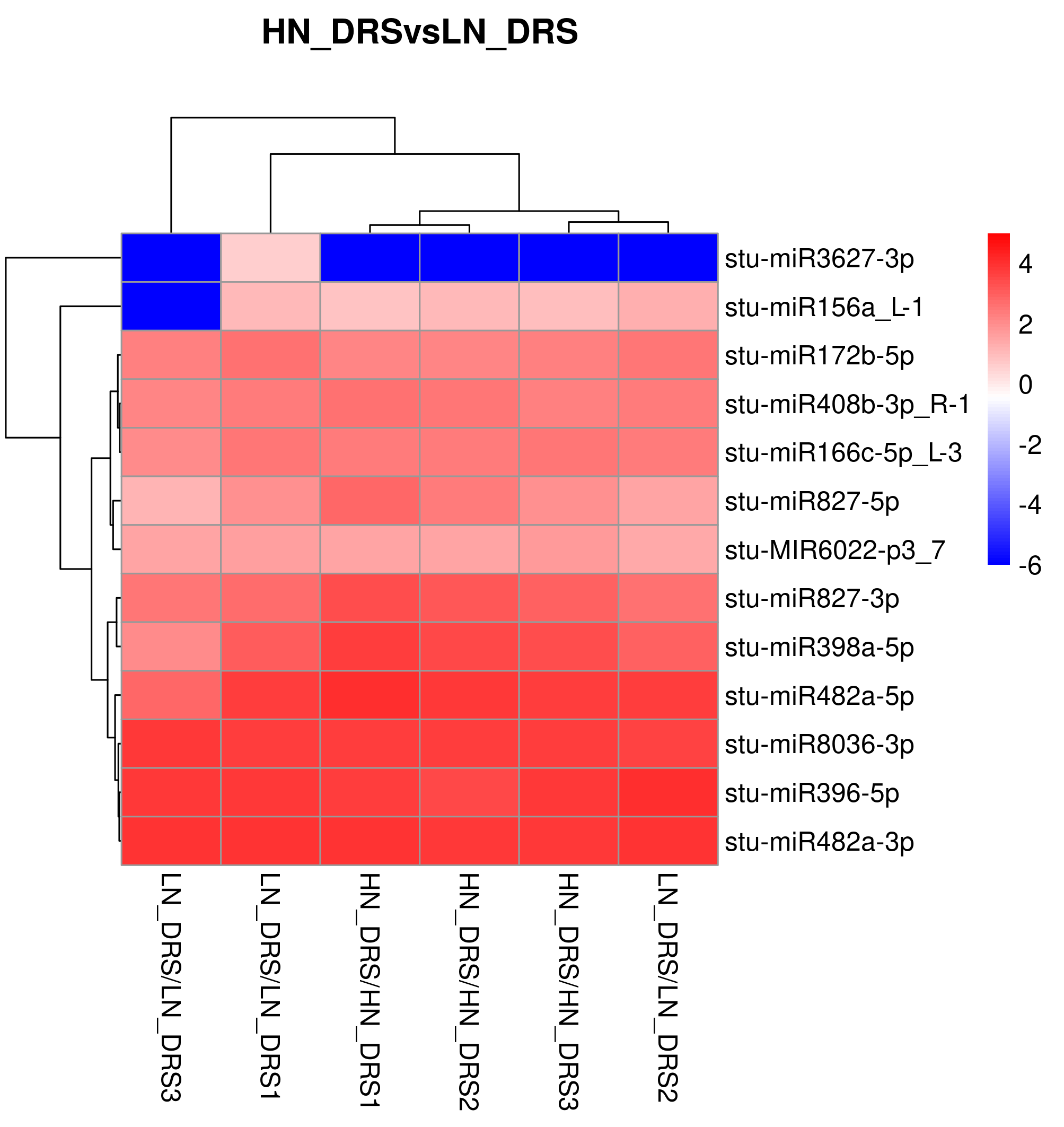

Supplement: Supplementary file 2 — Additional file 2: Figure 1. Analysis of variability among groups of differential miRNAsHN: excessive N application; LN: no N application; Y: Yanshu4; D: Atlantic;S: seedling stage; A: budding stage (A) LN_YLSvsLN_DLS (B) LN_YLAvsLN_DLA (C) HN_YLSvsHN_DLS. (D)HN_YLAvsHN_DLA (E) HN_DLSvsLN_DLS (F) HN_DLAvsLN_DLA. (G) HN_YLSvsLN_YLS (H)HN_YLAvsLN_YLA (I) LN_DLAvsLN_DLS. (J)HN_DLAvsHN_DLS (K)LN_YLAvsLN_YLS (L)HN_YLAvsHN_YLS.(M)LN_YRSvsLN_DRS (N)LN_YRAvsLN_DRA (O)HN_YRSvsHN_DRS. (P)HN_YRAvsHN_DRA (Q)HN_DRSvsLN_DRS(R)HN_DRAvsLN_DRA. (S)HN_YRSvsLN_YRS (T)HN_YRAvsLN_YRA (U)LN_DRAvsLN_DRS. (V)HN_DRAvsHN_DRS(W)LN_YRAvsLN_YRS (X)HN_YRAvsHN_YRS. Using the values of log2(fold change) as the horizontalcoordinate and -log10(p-value) as the vertical coordinate, volcanoplots were constructed for all miRNAs during differential expression analysis.The horizontal coordinate represents the fold change in the differentialexpression of miRNAs in different samples, and the vertical coordinaterepresents the statistical significance of the difference in the change inexpression levels of miRNAs. The red color represents up-regulatedsignificantly differentially expressed genes, the blue color representsdown-regulated significantly differentially expressed genes, and the grey dots representnon-significant differentially expressed genes. Figure 2. Comparison between groups of differential miRNAs. HN: excess N; LN:no N; Y: Yanshu4; D: Atlantic; S: seedling stage; A: budding stage. Thehorizontal coordinates indicate the data obtained after the comparison ofgroups, and the vertical coordinates indicate the number of up- anddown-regulated miRNAs. The red color represents up-regulated miRNAs, the bluecolor represents down-regulated miRNAs, and the numbers represent the number ofup- and down-regulated miRNAs. Figure 3. Results of clustering analysis ofdifferential miRNAs. HN: excess N; LN: no N; Y: Yanshu4; D: Atlantic; S: seedling;A: bud onset. (A)LN_YLSvsLN_DLS (B)LN_YLAvsLN_DLA (C)HN_YLSvsHN_DLS. [file 12870_2022_3866_MOESM2_ESM.zip › Appendix B Figure 3-Q.tiff]

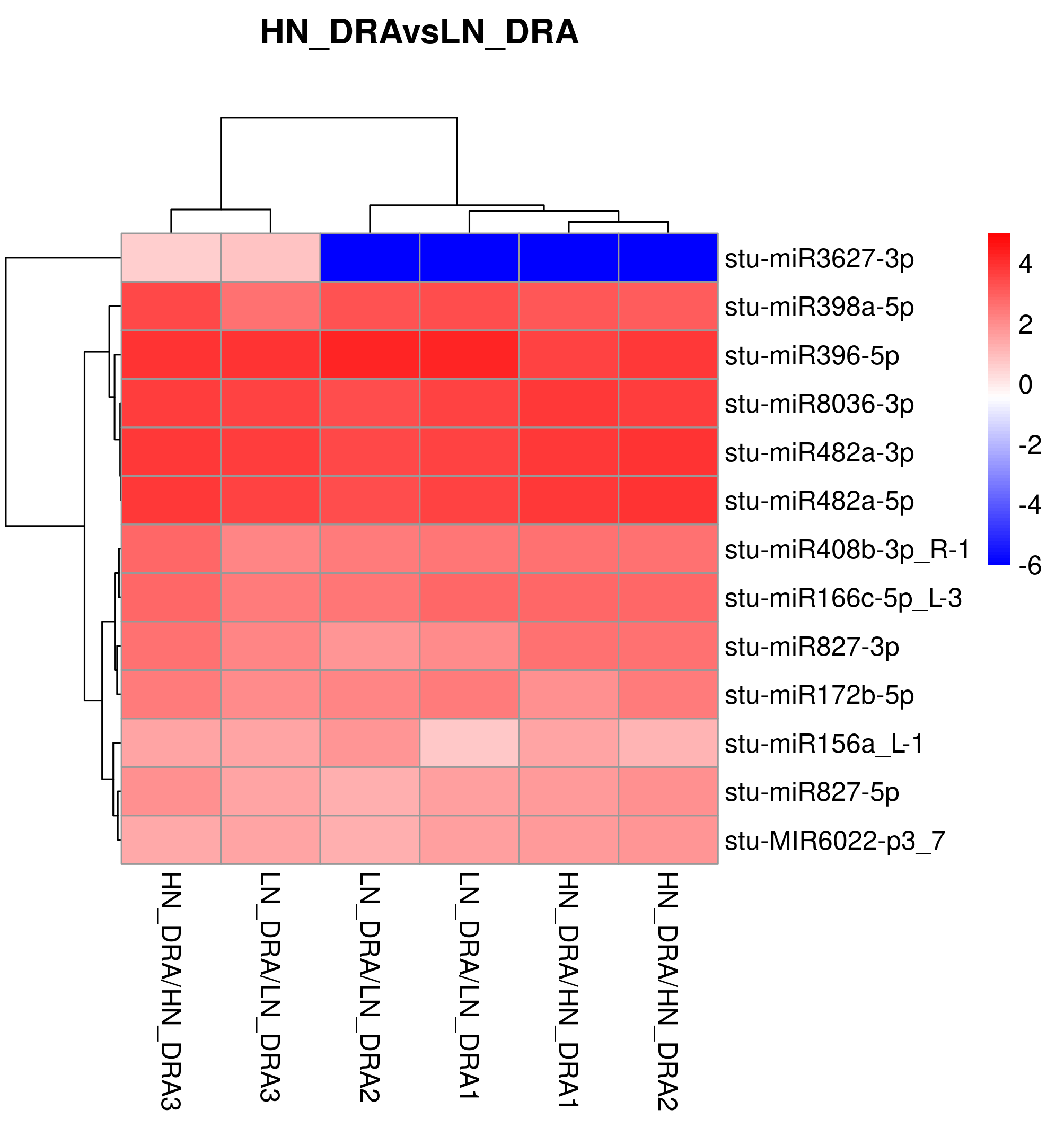

Supplement: Supplementary file 2 — Additional file 2: Figure 1. Analysis of variability among groups of differential miRNAsHN: excessive N application; LN: no N application; Y: Yanshu4; D: Atlantic;S: seedling stage; A: budding stage (A) LN_YLSvsLN_DLS (B) LN_YLAvsLN_DLA (C) HN_YLSvsHN_DLS. (D)HN_YLAvsHN_DLA (E) HN_DLSvsLN_DLS (F) HN_DLAvsLN_DLA. (G) HN_YLSvsLN_YLS (H)HN_YLAvsLN_YLA (I) LN_DLAvsLN_DLS. (J)HN_DLAvsHN_DLS (K)LN_YLAvsLN_YLS (L)HN_YLAvsHN_YLS.(M)LN_YRSvsLN_DRS (N)LN_YRAvsLN_DRA (O)HN_YRSvsHN_DRS. (P)HN_YRAvsHN_DRA (Q)HN_DRSvsLN_DRS(R)HN_DRAvsLN_DRA. (S)HN_YRSvsLN_YRS (T)HN_YRAvsLN_YRA (U)LN_DRAvsLN_DRS. (V)HN_DRAvsHN_DRS(W)LN_YRAvsLN_YRS (X)HN_YRAvsHN_YRS. Using the values of log2(fold change) as the horizontalcoordinate and -log10(p-value) as the vertical coordinate, volcanoplots were constructed for all miRNAs during differential expression analysis.The horizontal coordinate represents the fold change in the differentialexpression of miRNAs in different samples, and the vertical coordinaterepresents the statistical significance of the difference in the change inexpression levels of miRNAs. The red color represents up-regulatedsignificantly differentially expressed genes, the blue color representsdown-regulated significantly differentially expressed genes, and the grey dots representnon-significant differentially expressed genes. Figure 2. Comparison between groups of differential miRNAs. HN: excess N; LN:no N; Y: Yanshu4; D: Atlantic; S: seedling stage; A: budding stage. Thehorizontal coordinates indicate the data obtained after the comparison ofgroups, and the vertical coordinates indicate the number of up- anddown-regulated miRNAs. The red color represents up-regulated miRNAs, the bluecolor represents down-regulated miRNAs, and the numbers represent the number ofup- and down-regulated miRNAs. Figure 3. Results of clustering analysis ofdifferential miRNAs. HN: excess N; LN: no N; Y: Yanshu4; D: Atlantic; S: seedling;A: bud onset. (A)LN_YLSvsLN_DLS (B)LN_YLAvsLN_DLA (C)HN_YLSvsHN_DLS. [file 12870_2022_3866_MOESM2_ESM.zip › Appendix B Figure 3-R.tiff]

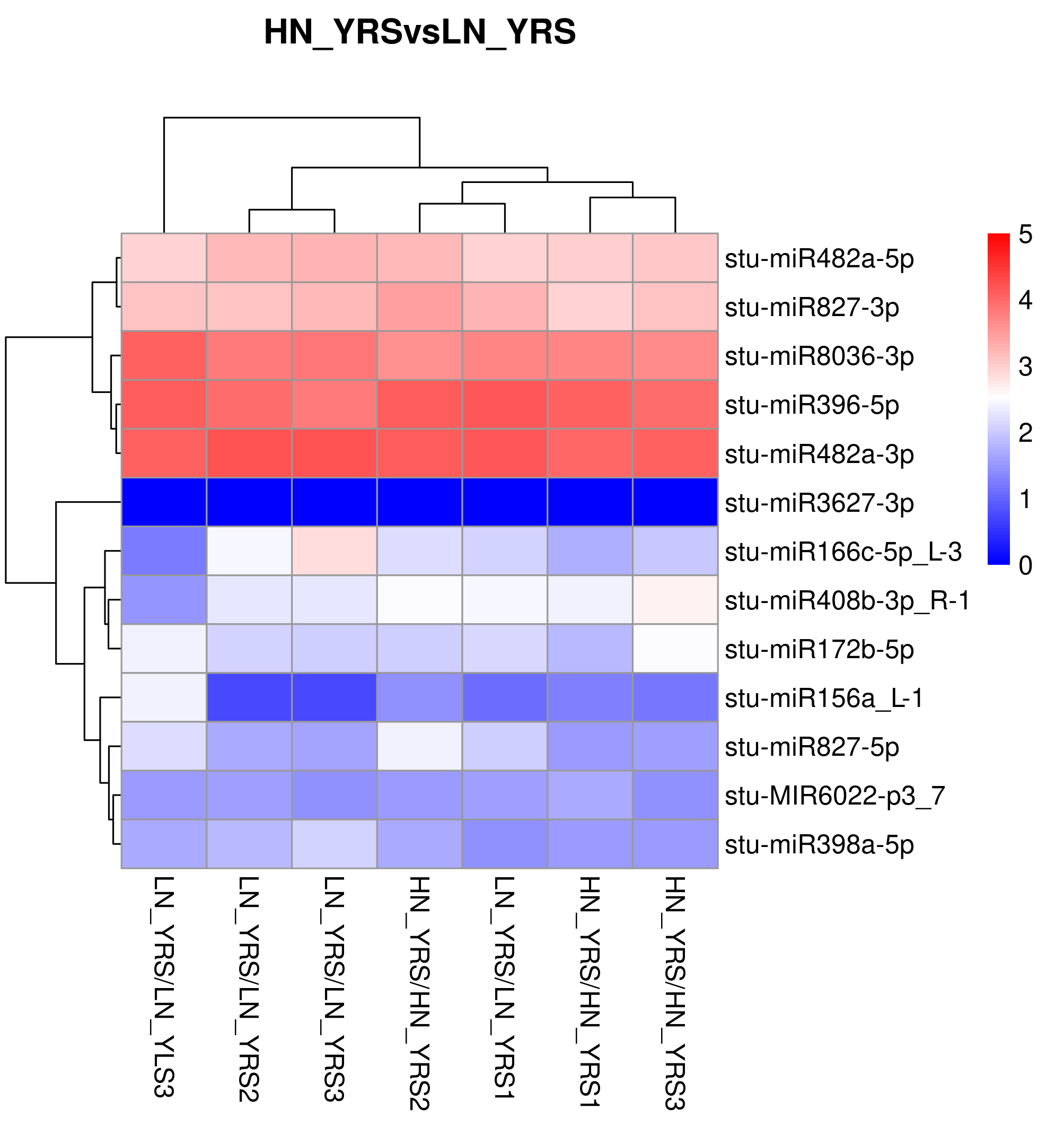

Supplement: Supplementary file 2 — Additional file 2: Figure 1. Analysis of variability among groups of differential miRNAsHN: excessive N application; LN: no N application; Y: Yanshu4; D: Atlantic;S: seedling stage; A: budding stage (A) LN_YLSvsLN_DLS (B) LN_YLAvsLN_DLA (C) HN_YLSvsHN_DLS. (D)HN_YLAvsHN_DLA (E) HN_DLSvsLN_DLS (F) HN_DLAvsLN_DLA. (G) HN_YLSvsLN_YLS (H)HN_YLAvsLN_YLA (I) LN_DLAvsLN_DLS. (J)HN_DLAvsHN_DLS (K)LN_YLAvsLN_YLS (L)HN_YLAvsHN_YLS.(M)LN_YRSvsLN_DRS (N)LN_YRAvsLN_DRA (O)HN_YRSvsHN_DRS. (P)HN_YRAvsHN_DRA (Q)HN_DRSvsLN_DRS(R)HN_DRAvsLN_DRA. (S)HN_YRSvsLN_YRS (T)HN_YRAvsLN_YRA (U)LN_DRAvsLN_DRS. (V)HN_DRAvsHN_DRS(W)LN_YRAvsLN_YRS (X)HN_YRAvsHN_YRS. Using the values of log2(fold change) as the horizontalcoordinate and -log10(p-value) as the vertical coordinate, volcanoplots were constructed for all miRNAs during differential expression analysis.The horizontal coordinate represents the fold change in the differentialexpression of miRNAs in different samples, and the vertical coordinaterepresents the statistical significance of the difference in the change inexpression levels of miRNAs. The red color represents up-regulatedsignificantly differentially expressed genes, the blue color representsdown-regulated significantly differentially expressed genes, and the grey dots representnon-significant differentially expressed genes. Figure 2. Comparison between groups of differential miRNAs. HN: excess N; LN:no N; Y: Yanshu4; D: Atlantic; S: seedling stage; A: budding stage. Thehorizontal coordinates indicate the data obtained after the comparison ofgroups, and the vertical coordinates indicate the number of up- anddown-regulated miRNAs. The red color represents up-regulated miRNAs, the bluecolor represents down-regulated miRNAs, and the numbers represent the number ofup- and down-regulated miRNAs. Figure 3. Results of clustering analysis ofdifferential miRNAs. HN: excess N; LN: no N; Y: Yanshu4; D: Atlantic; S: seedling;A: bud onset. (A)LN_YLSvsLN_DLS (B)LN_YLAvsLN_DLA (C)HN_YLSvsHN_DLS. [file 12870_2022_3866_MOESM2_ESM.zip › Appendix B Figure 3-S.tiff]

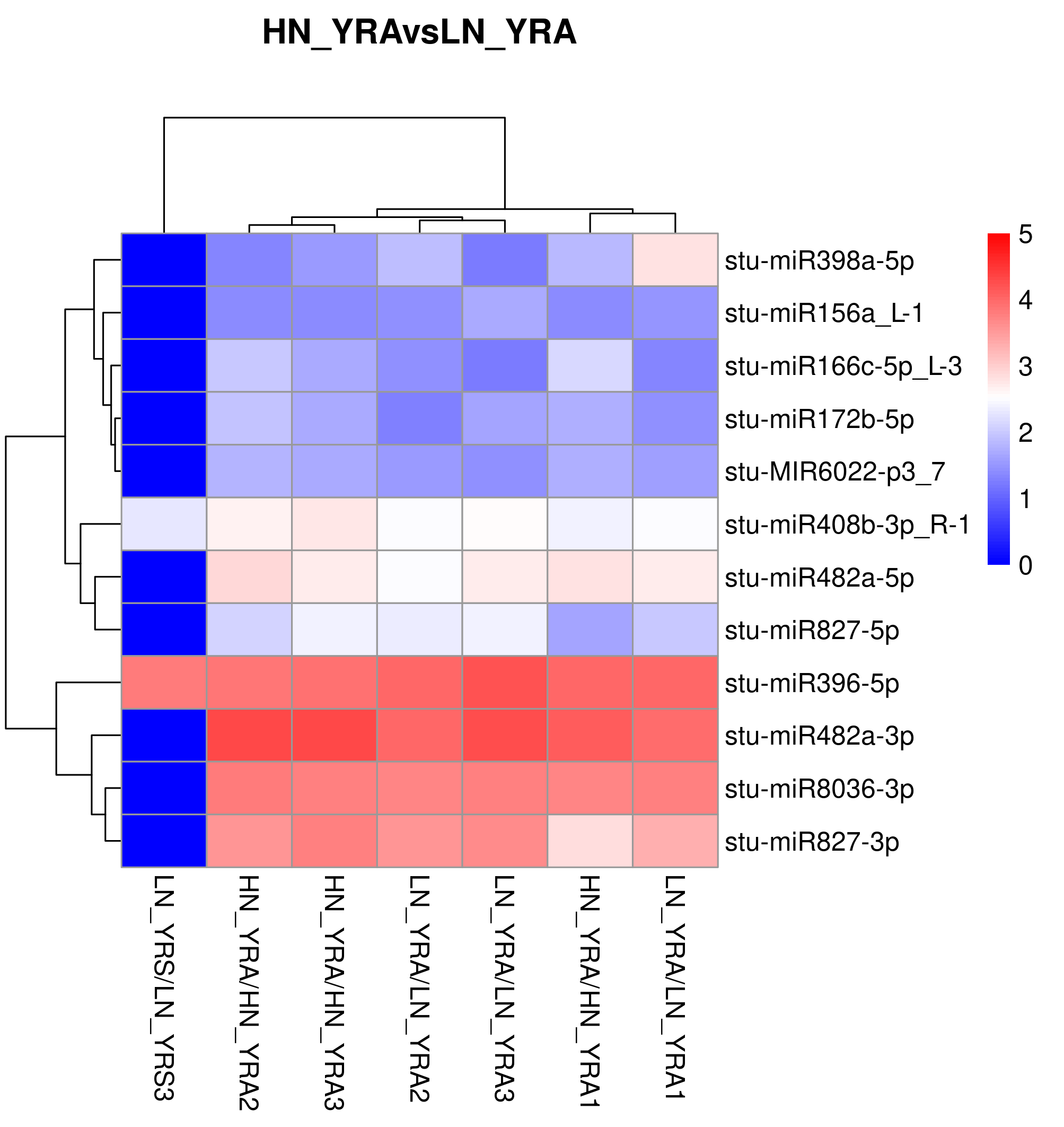

Supplement: Supplementary file 2 — Additional file 2: Figure 1. Analysis of variability among groups of differential miRNAsHN: excessive N application; LN: no N application; Y: Yanshu4; D: Atlantic;S: seedling stage; A: budding stage (A) LN_YLSvsLN_DLS (B) LN_YLAvsLN_DLA (C) HN_YLSvsHN_DLS. (D)HN_YLAvsHN_DLA (E) HN_DLSvsLN_DLS (F) HN_DLAvsLN_DLA. (G) HN_YLSvsLN_YLS (H)HN_YLAvsLN_YLA (I) LN_DLAvsLN_DLS. (J)HN_DLAvsHN_DLS (K)LN_YLAvsLN_YLS (L)HN_YLAvsHN_YLS.(M)LN_YRSvsLN_DRS (N)LN_YRAvsLN_DRA (O)HN_YRSvsHN_DRS. (P)HN_YRAvsHN_DRA (Q)HN_DRSvsLN_DRS(R)HN_DRAvsLN_DRA. (S)HN_YRSvsLN_YRS (T)HN_YRAvsLN_YRA (U)LN_DRAvsLN_DRS. (V)HN_DRAvsHN_DRS(W)LN_YRAvsLN_YRS (X)HN_YRAvsHN_YRS. Using the values of log2(fold change) as the horizontalcoordinate and -log10(p-value) as the vertical coordinate, volcanoplots were constructed for all miRNAs during differential expression analysis.The horizontal coordinate represents the fold change in the differentialexpression of miRNAs in different samples, and the vertical coordinaterepresents the statistical significance of the difference in the change inexpression levels of miRNAs. The red color represents up-regulatedsignificantly differentially expressed genes, the blue color representsdown-regulated significantly differentially expressed genes, and the grey dots representnon-significant differentially expressed genes. Figure 2. Comparison between groups of differential miRNAs. HN: excess N; LN:no N; Y: Yanshu4; D: Atlantic; S: seedling stage; A: budding stage. Thehorizontal coordinates indicate the data obtained after the comparison ofgroups, and the vertical coordinates indicate the number of up- anddown-regulated miRNAs. The red color represents up-regulated miRNAs, the bluecolor represents down-regulated miRNAs, and the numbers represent the number ofup- and down-regulated miRNAs. Figure 3. Results of clustering analysis ofdifferential miRNAs. HN: excess N; LN: no N; Y: Yanshu4; D: Atlantic; S: seedling;A: bud onset. (A)LN_YLSvsLN_DLS (B)LN_YLAvsLN_DLA (C)HN_YLSvsHN_DLS. [file 12870_2022_3866_MOESM2_ESM.zip › Appendix B Figure 3-T.tiff]

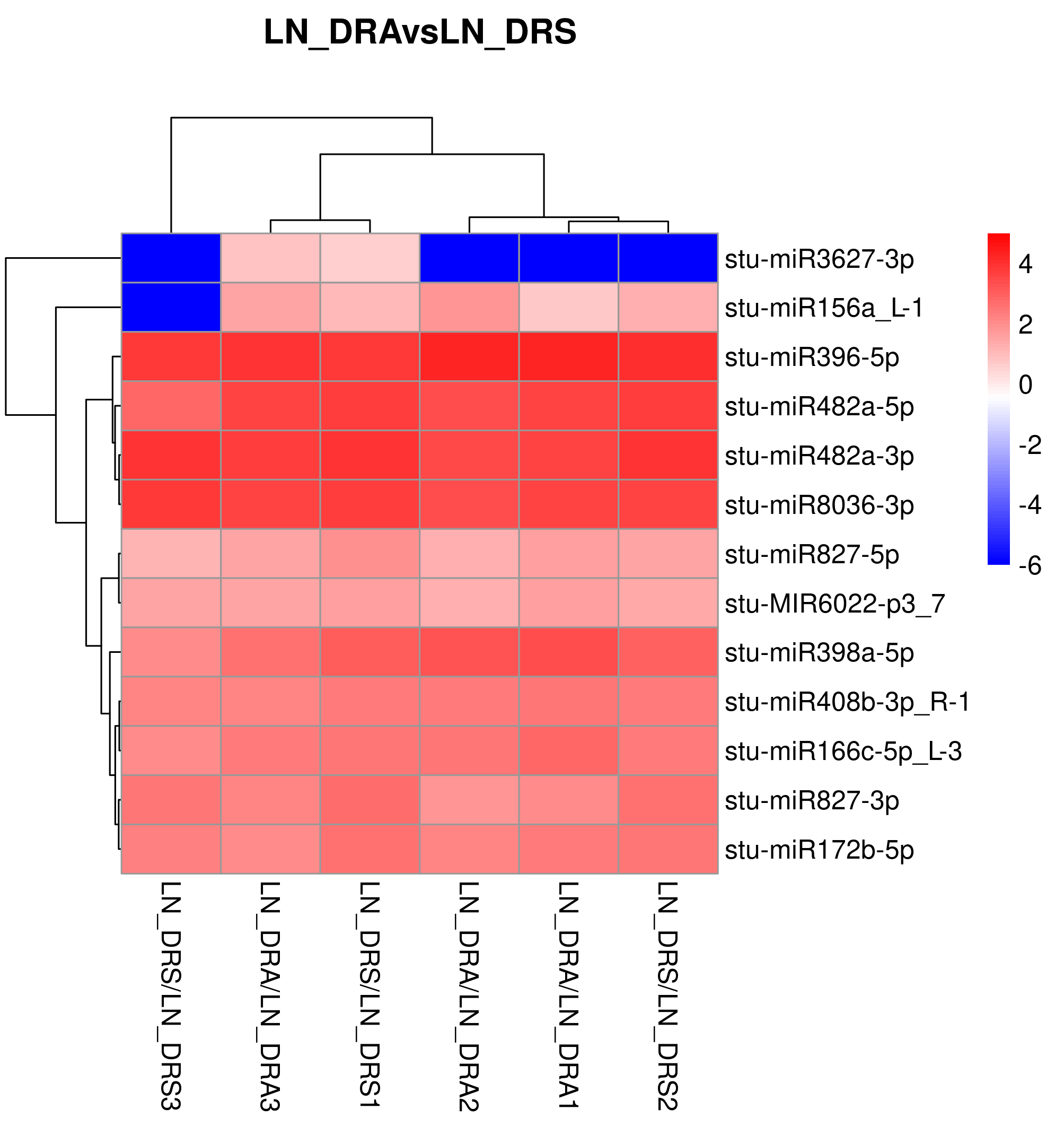

Supplement: Supplementary file 2 — Additional file 2: Figure 1. Analysis of variability among groups of differential miRNAsHN: excessive N application; LN: no N application; Y: Yanshu4; D: Atlantic;S: seedling stage; A: budding stage (A) LN_YLSvsLN_DLS (B) LN_YLAvsLN_DLA (C) HN_YLSvsHN_DLS. (D)HN_YLAvsHN_DLA (E) HN_DLSvsLN_DLS (F) HN_DLAvsLN_DLA. (G) HN_YLSvsLN_YLS (H)HN_YLAvsLN_YLA (I) LN_DLAvsLN_DLS. (J)HN_DLAvsHN_DLS (K)LN_YLAvsLN_YLS (L)HN_YLAvsHN_YLS.(M)LN_YRSvsLN_DRS (N)LN_YRAvsLN_DRA (O)HN_YRSvsHN_DRS. (P)HN_YRAvsHN_DRA (Q)HN_DRSvsLN_DRS(R)HN_DRAvsLN_DRA. (S)HN_YRSvsLN_YRS (T)HN_YRAvsLN_YRA (U)LN_DRAvsLN_DRS. (V)HN_DRAvsHN_DRS(W)LN_YRAvsLN_YRS (X)HN_YRAvsHN_YRS. Using the values of log2(fold change) as the horizontalcoordinate and -log10(p-value) as the vertical coordinate, volcanoplots were constructed for all miRNAs during differential expression analysis.The horizontal coordinate represents the fold change in the differentialexpression of miRNAs in different samples, and the vertical coordinaterepresents the statistical significance of the difference in the change inexpression levels of miRNAs. The red color represents up-regulatedsignificantly differentially expressed genes, the blue color representsdown-regulated significantly differentially expressed genes, and the grey dots representnon-significant differentially expressed genes. Figure 2. Comparison between groups of differential miRNAs. HN: excess N; LN:no N; Y: Yanshu4; D: Atlantic; S: seedling stage; A: budding stage. Thehorizontal coordinates indicate the data obtained after the comparison ofgroups, and the vertical coordinates indicate the number of up- anddown-regulated miRNAs. The red color represents up-regulated miRNAs, the bluecolor represents down-regulated miRNAs, and the numbers represent the number ofup- and down-regulated miRNAs. Figure 3. Results of clustering analysis ofdifferential miRNAs. HN: excess N; LN: no N; Y: Yanshu4; D: Atlantic; S: seedling;A: bud onset. (A)LN_YLSvsLN_DLS (B)LN_YLAvsLN_DLA (C)HN_YLSvsHN_DLS. [file 12870_2022_3866_MOESM2_ESM.zip › Appendix B Figure 3-U.tiff]

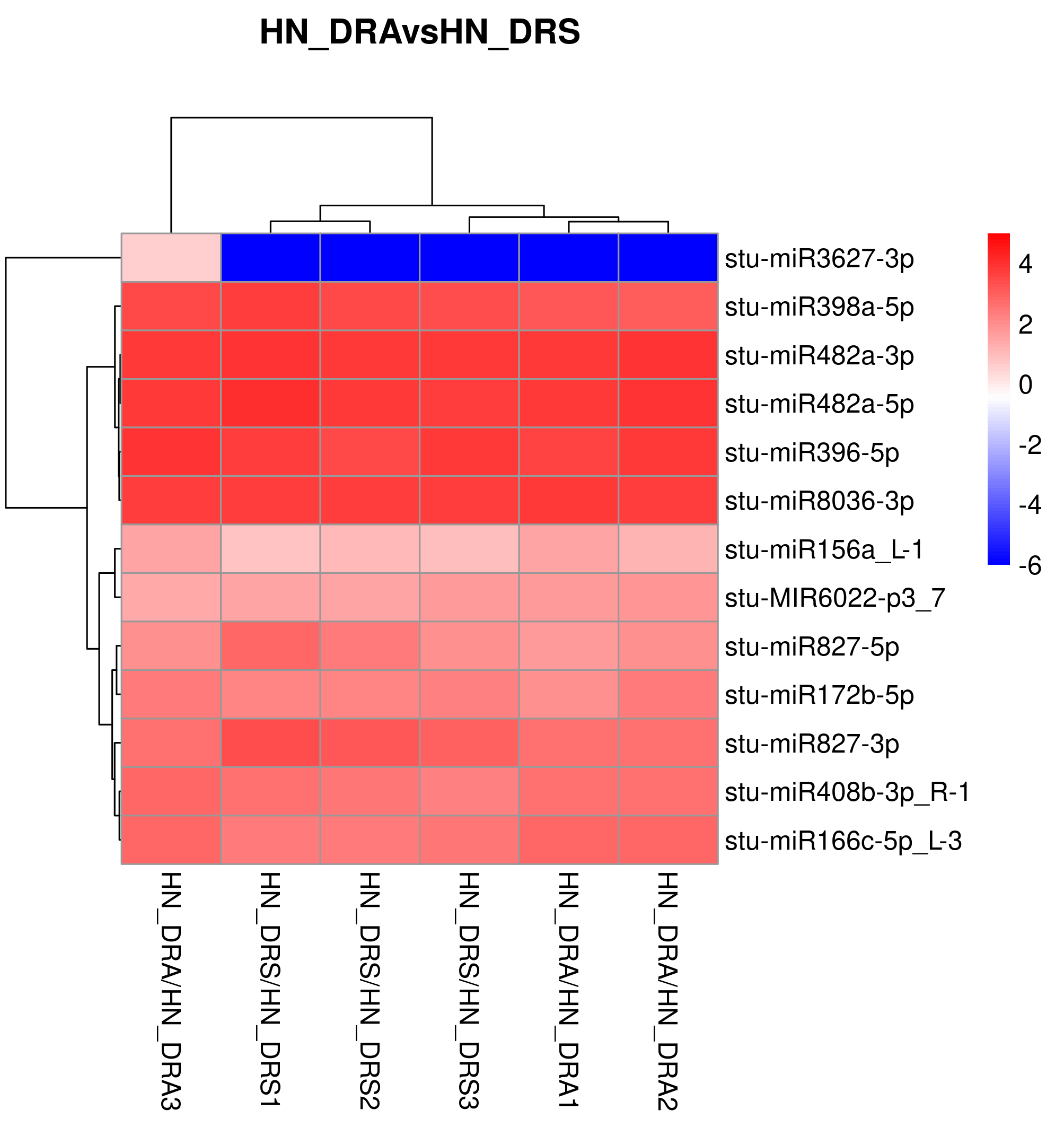

Supplement: Supplementary file 2 — Additional file 2: Figure 1. Analysis of variability among groups of differential miRNAsHN: excessive N application; LN: no N application; Y: Yanshu4; D: Atlantic;S: seedling stage; A: budding stage (A) LN_YLSvsLN_DLS (B) LN_YLAvsLN_DLA (C) HN_YLSvsHN_DLS. (D)HN_YLAvsHN_DLA (E) HN_DLSvsLN_DLS (F) HN_DLAvsLN_DLA. (G) HN_YLSvsLN_YLS (H)HN_YLAvsLN_YLA (I) LN_DLAvsLN_DLS. (J)HN_DLAvsHN_DLS (K)LN_YLAvsLN_YLS (L)HN_YLAvsHN_YLS.(M)LN_YRSvsLN_DRS (N)LN_YRAvsLN_DRA (O)HN_YRSvsHN_DRS. (P)HN_YRAvsHN_DRA (Q)HN_DRSvsLN_DRS(R)HN_DRAvsLN_DRA. (S)HN_YRSvsLN_YRS (T)HN_YRAvsLN_YRA (U)LN_DRAvsLN_DRS. (V)HN_DRAvsHN_DRS(W)LN_YRAvsLN_YRS (X)HN_YRAvsHN_YRS. Using the values of log2(fold change) as the horizontalcoordinate and -log10(p-value) as the vertical coordinate, volcanoplots were constructed for all miRNAs during differential expression analysis.The horizontal coordinate represents the fold change in the differentialexpression of miRNAs in different samples, and the vertical coordinaterepresents the statistical significance of the difference in the change inexpression levels of miRNAs. The red color represents up-regulatedsignificantly differentially expressed genes, the blue color representsdown-regulated significantly differentially expressed genes, and the grey dots representnon-significant differentially expressed genes. Figure 2. Comparison between groups of differential miRNAs. HN: excess N; LN:no N; Y: Yanshu4; D: Atlantic; S: seedling stage; A: budding stage. Thehorizontal coordinates indicate the data obtained after the comparison ofgroups, and the vertical coordinates indicate the number of up- anddown-regulated miRNAs. The red color represents up-regulated miRNAs, the bluecolor represents down-regulated miRNAs, and the numbers represent the number ofup- and down-regulated miRNAs. Figure 3. Results of clustering analysis ofdifferential miRNAs. HN: excess N; LN: no N; Y: Yanshu4; D: Atlantic; S: seedling;A: bud onset. (A)LN_YLSvsLN_DLS (B)LN_YLAvsLN_DLA (C)HN_YLSvsHN_DLS. [file 12870_2022_3866_MOESM2_ESM.zip › Appendix B Figure 3-V.tiff]

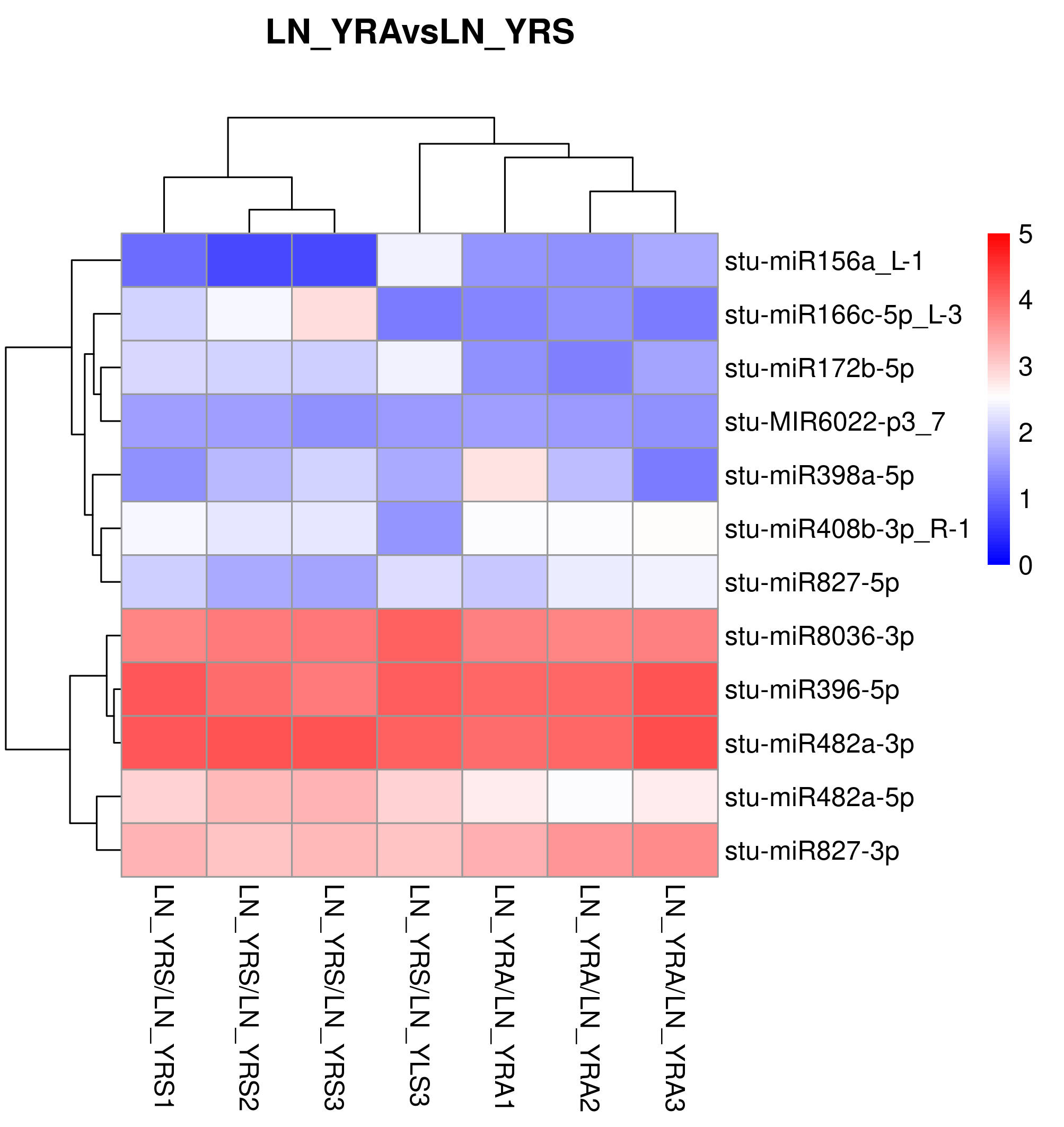

Supplement: Supplementary file 2 — Additional file 2: Figure 1. Analysis of variability among groups of differential miRNAsHN: excessive N application; LN: no N application; Y: Yanshu4; D: Atlantic;S: seedling stage; A: budding stage (A) LN_YLSvsLN_DLS (B) LN_YLAvsLN_DLA (C) HN_YLSvsHN_DLS. (D)HN_YLAvsHN_DLA (E) HN_DLSvsLN_DLS (F) HN_DLAvsLN_DLA. (G) HN_YLSvsLN_YLS (H)HN_YLAvsLN_YLA (I) LN_DLAvsLN_DLS. (J)HN_DLAvsHN_DLS (K)LN_YLAvsLN_YLS (L)HN_YLAvsHN_YLS.(M)LN_YRSvsLN_DRS (N)LN_YRAvsLN_DRA (O)HN_YRSvsHN_DRS. (P)HN_YRAvsHN_DRA (Q)HN_DRSvsLN_DRS(R)HN_DRAvsLN_DRA. (S)HN_YRSvsLN_YRS (T)HN_YRAvsLN_YRA (U)LN_DRAvsLN_DRS. (V)HN_DRAvsHN_DRS(W)LN_YRAvsLN_YRS (X)HN_YRAvsHN_YRS. Using the values of log2(fold change) as the horizontalcoordinate and -log10(p-value) as the vertical coordinate, volcanoplots were constructed for all miRNAs during differential expression analysis.The horizontal coordinate represents the fold change in the differentialexpression of miRNAs in different samples, and the vertical coordinaterepresents the statistical significance of the difference in the change inexpression levels of miRNAs. The red color represents up-regulatedsignificantly differentially expressed genes, the blue color representsdown-regulated significantly differentially expressed genes, and the grey dots representnon-significant differentially expressed genes. Figure 2. Comparison between groups of differential miRNAs. HN: excess N; LN:no N; Y: Yanshu4; D: Atlantic; S: seedling stage; A: budding stage. Thehorizontal coordinates indicate the data obtained after the comparison ofgroups, and the vertical coordinates indicate the number of up- anddown-regulated miRNAs. The red color represents up-regulated miRNAs, the bluecolor represents down-regulated miRNAs, and the numbers represent the number ofup- and down-regulated miRNAs. Figure 3. Results of clustering analysis ofdifferential miRNAs. HN: excess N; LN: no N; Y: Yanshu4; D: Atlantic; S: seedling;A: bud onset. (A)LN_YLSvsLN_DLS (B)LN_YLAvsLN_DLA (C)HN_YLSvsHN_DLS. [file 12870_2022_3866_MOESM2_ESM.zip › Appendix B Figure 3-W.tiff]

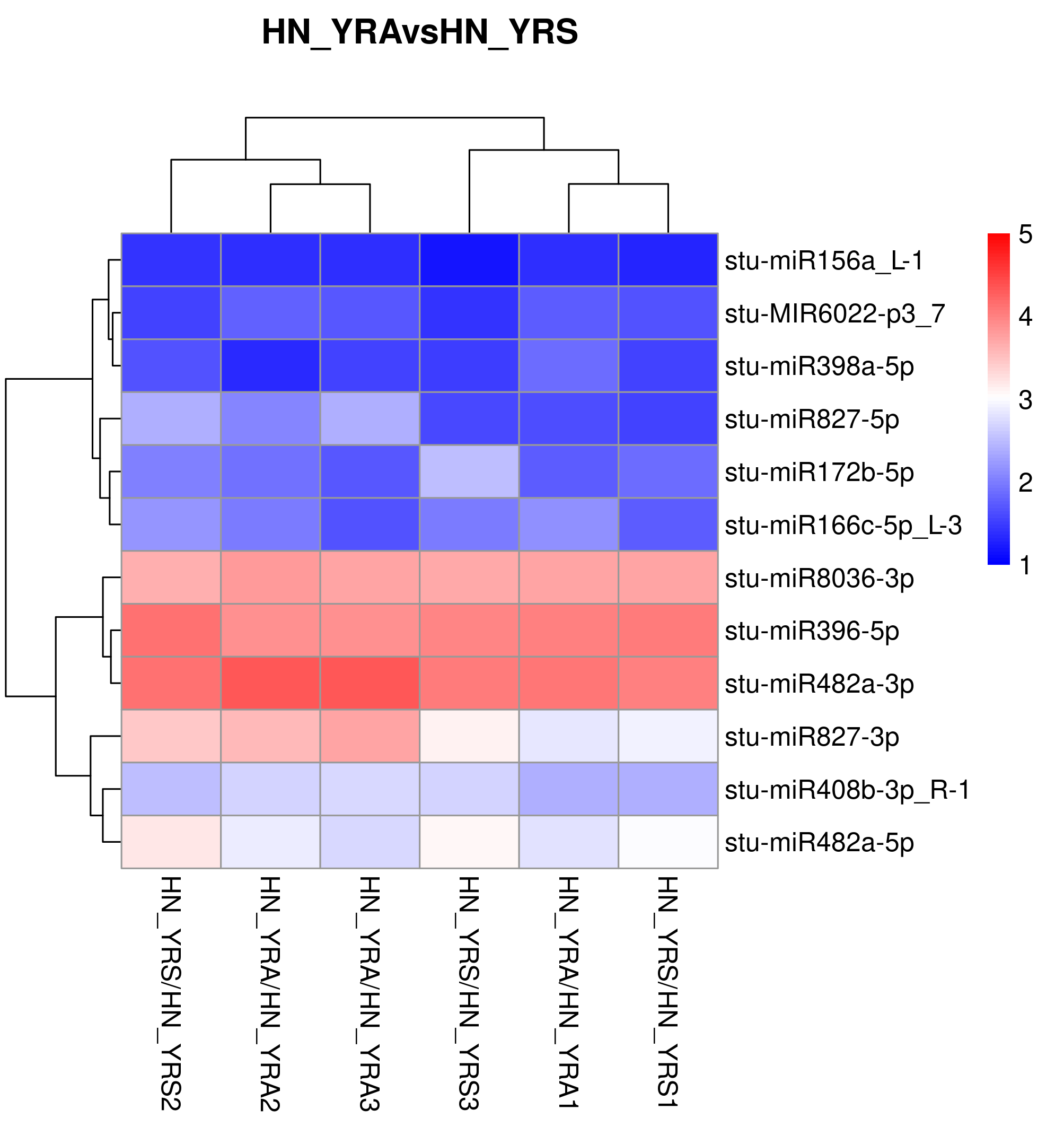

Supplement: Supplementary file 2 — Additional file 2: Figure 1. Analysis of variability among groups of differential miRNAsHN: excessive N application; LN: no N application; Y: Yanshu4; D: Atlantic;S: seedling stage; A: budding stage (A) LN_YLSvsLN_DLS (B) LN_YLAvsLN_DLA (C) HN_YLSvsHN_DLS. (D)HN_YLAvsHN_DLA (E) HN_DLSvsLN_DLS (F) HN_DLAvsLN_DLA. (G) HN_YLSvsLN_YLS (H)HN_YLAvsLN_YLA (I) LN_DLAvsLN_DLS. (J)HN_DLAvsHN_DLS (K)LN_YLAvsLN_YLS (L)HN_YLAvsHN_YLS.(M)LN_YRSvsLN_DRS (N)LN_YRAvsLN_DRA (O)HN_YRSvsHN_DRS. (P)HN_YRAvsHN_DRA (Q)HN_DRSvsLN_DRS(R)HN_DRAvsLN_DRA. (S)HN_YRSvsLN_YRS (T)HN_YRAvsLN_YRA (U)LN_DRAvsLN_DRS. (V)HN_DRAvsHN_DRS(W)LN_YRAvsLN_YRS (X)HN_YRAvsHN_YRS. Using the values of log2(fold change) as the horizontalcoordinate and -log10(p-value) as the vertical coordinate, volcanoplots were constructed for all miRNAs during differential expression analysis.The horizontal coordinate represents the fold change in the differentialexpression of miRNAs in different samples, and the vertical coordinaterepresents the statistical significance of the difference in the change inexpression levels of miRNAs. The red color represents up-regulatedsignificantly differentially expressed genes, the blue color representsdown-regulated significantly differentially expressed genes, and the grey dots representnon-significant differentially expressed genes. Figure 2. Comparison between groups of differential miRNAs. HN: excess N; LN:no N; Y: Yanshu4; D: Atlantic; S: seedling stage; A: budding stage. Thehorizontal coordinates indicate the data obtained after the comparison ofgroups, and the vertical coordinates indicate the number of up- anddown-regulated miRNAs. The red color represents up-regulated miRNAs, the bluecolor represents down-regulated miRNAs, and the numbers represent the number ofup- and down-regulated miRNAs. Figure 3. Results of clustering analysis ofdifferential miRNAs. HN: excess N; LN: no N; Y: Yanshu4; D: Atlantic; S: seedling;A: bud onset. (A)LN_YLSvsLN_DLS (B)LN_YLAvsLN_DLA (C)HN_YLSvsHN_DLS. [file 12870_2022_3866_MOESM2_ESM.zip › Appendix B Figure 3-X.tiff]

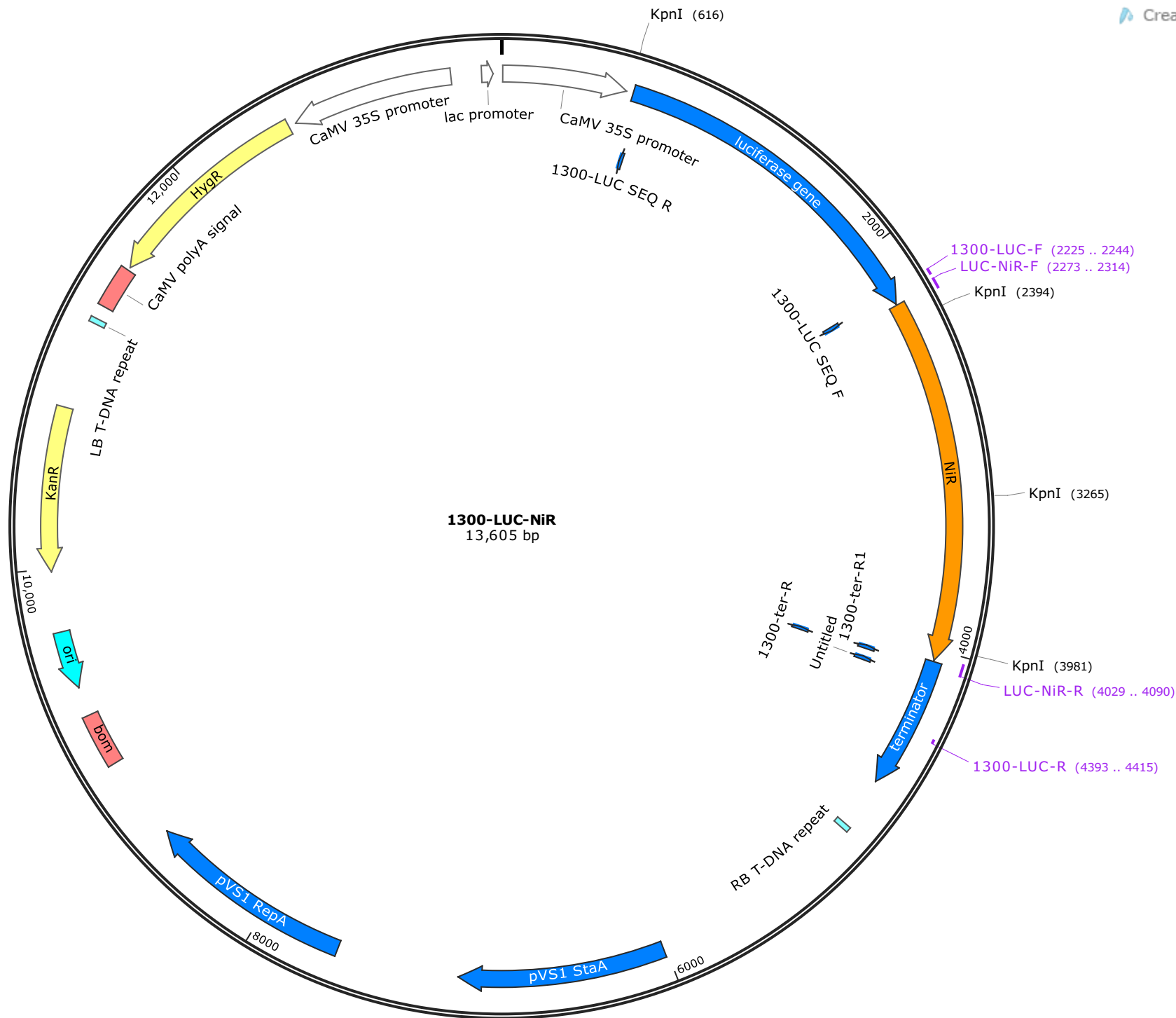

Supplement: Supplementary file 4 — Additional file 4: Figure 1. Construction of therecombinant plasmid pCAMBIA1300-LUC-NiR for the luciferase reporter vector. Figure 2. Construction of the recombinant plasmid pCAMBIA1300-35S-396-5p for theluciferase localization report vector. [file 12870_2022_3866_MOESM4_ESM.zip › Appendix D Figure 1.pdf]

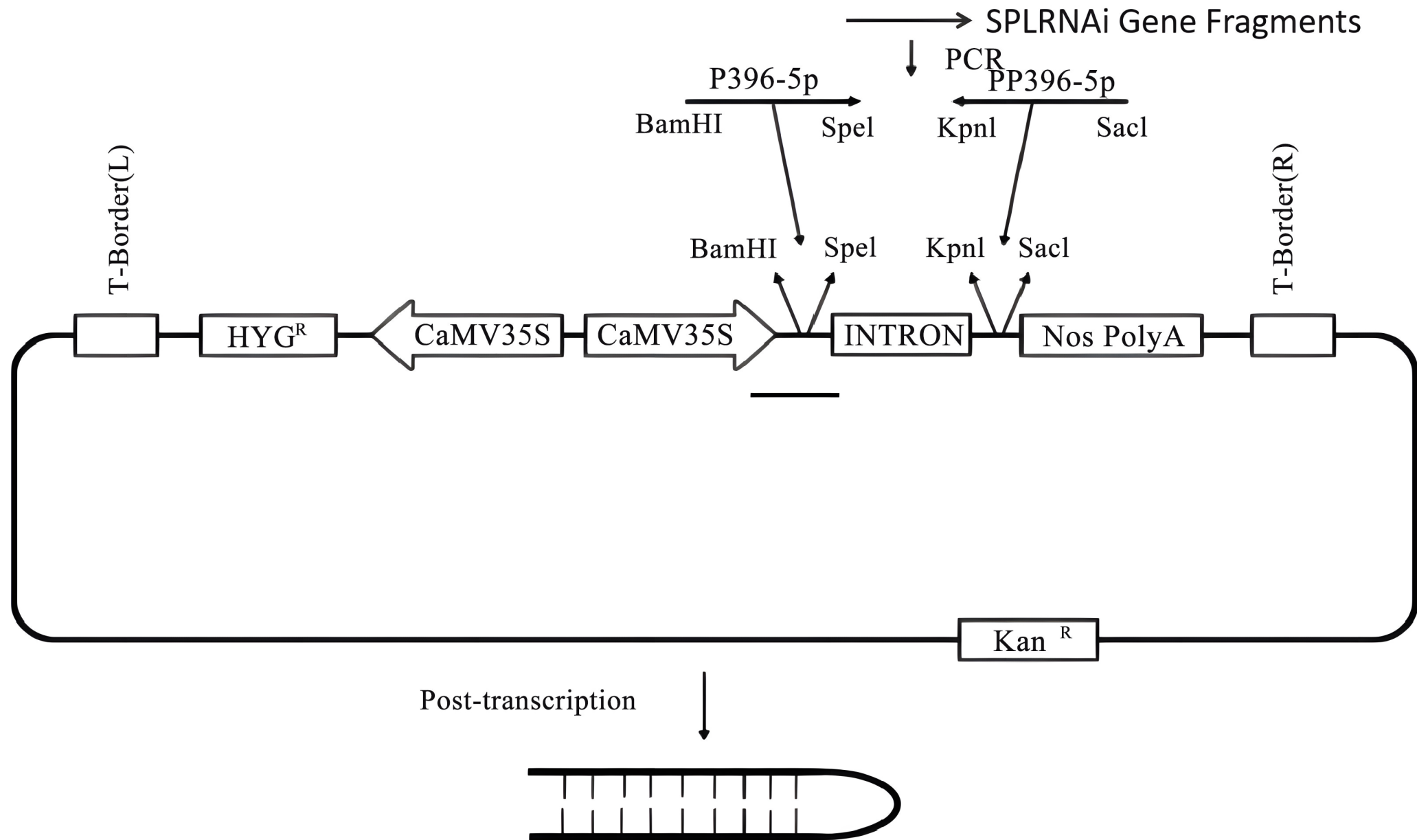

Supplement: Supplementary file 4 — Additional file 4: Figure 1. Construction of therecombinant plasmid pCAMBIA1300-LUC-NiR for the luciferase reporter vector. Figure 2. Construction of the recombinant plasmid pCAMBIA1300-35S-396-5p for theluciferase localization report vector. [file 12870_2022_3866_MOESM4_ESM.zip › Appendix D Figure 2.pdf]
