# Supplementary material for: Screening of differentially expressed microRNAs and target genes in two potato varieties under nitrogen stress
Source: BMC Plant Biol. 2022 Oct 8;22:478. doi: 10.1186/s12870-022-03866-5 (PMC9547441; doi:10.1186/s12870-022-03866-5)
Supplement: Supplementary file 3 — Additional file 3. [file 12870_2022_3866_MOESM3_ESM.docx]

Appendix C

#### >NiR

#### ATGACATCTTTTTCGGTTAAATTTTCAGCTACTTCACTTCCAAATTCTAATAGATTTTCCAAGCTTCATGCTACTCCACCGCAGACGGTGGCGGTACCGTCATCTGGGGCGGCGGAGATAGCTGCTGAGAGACTAGAGCCTAGAGTGGAGCAAAAAGATGGGTATTGGGTACTTAAGGAAAAGTTCAGACAAGGCATAAATCCAGCTGAAAAGGCGAAGATTGAAAAGGAACCTATGAAATTATTCACTGAAAATGGTATTGAAGATCTTGCTAAGATCTCGCTTGAAGAGATGGAGAAATCAAAGCTTACTAAGGAAGATATTGATATTCGCCTCAAGTGGCTTGGACTCTTCCATCGGAGAAAACACCACTATGGTCGATTCATGATGCGATTGAAGCTTCCAAATGGAGTAACGACGAGTGCTCAAACTCGATACTTAGCTAGTGTGATTAGGAAATATGGGAAAGATGGATGTGGTGATGTGACTACAAGGCAAAATTGGCAGATTCGTGGGGTTGTGTTACCTGATGTGCCTGAGATTCTAAAGGGACTTGATGAAGTTGGCTTGACAAGTCTGCAGAGTGGCATGGATAATGTTCGAAATCCGGTGGGGAACCCTCTTGCAGGGATTGATCCTCATGAAATTGTAGACACAAGACCTTACACTAATTTGCTGTCCCAATATGTCACCGCCAATTTTCTTGGCAATGTGGACGTGACTAACTTGCCAAGAAAGTGGAATGTATGTGTAATAGGGTCACACGATCTTTATGAGCATCCGCATATCAATGATCTTGCCTATATGCCTGCAACAAAAGATGGACGATTTGGATTCAACCTGCTTGTGGGTGGATTCTTCAGTCCGAAGCGATGTGCAGAGGCAATTCCTCTTGATGCATGGGTTCCAGCTGATGATGTTGTCCCTGTTTGCAAAGCAATATTAGAAGCTTATAGAGATCTTGGTACCCGAGGGAACAGGCAGAAAACAAGAATGATGTGGTTAATTGACGAACTTGGTGTTGAAGGATTCAGGGCAGAAGTTGTGAAGAGAATGCCCCAACAGAAGCTAGAGAGAGAATCTGCAGAGGATTTGGTCCAGAAACAATGGGAAAGGAGAGAGTACCTTGGCGTGCATCCGCAGAAACAGGAGGGTTACAGTTTTGTTGGTCTTCACATTCCCGTGGGTCGTGTCCAAGCAGATGACATGGACGAGCTAGCTCGTTTGGCAGATGAATATGGTTCAGGAGAGCTCCGTCTGACTGTTGAACAGAACATCATTATCCCCAACATCGAGAACTCAAAGATCGATGCATTACTCAATGAGCCTCTCCTAAAGAAGAGATTTTCTCCCGATCCACCTATTCTCATGAGAAATTTGGTGGCTTGTACTGGTAACCAATTCTGTGGGCAAGCAATAATCGAGACTAAAGCACGTTCAATGAAGATAACTGAGGAGGTTCAACGTCTAGTCTCCGTGACACAGCCAGTGAGGATGCACTGGACAGGTTGCCCAAATACATGTGGACAAGTTCAAGTTGCCGATATCGGATTCATGGGATGCCTGACTAGAAAGGAAGGCAAGACTGTGGAAGCTACTGATGTTTTCTTGGGTGGCAGAATAGGGAGCGACTCGCATTTAGGAGAAGTTTATAAGAAGTCTGTCCCCTGTGAAGATTTGGTACCAATAATCGTCGACTTACTAGTTAACAAGTTTGGTGCTGTTCCAAGAGAAAGAGAAGAAACAGAAGAGTAA
